# Directed biosynthesis of the designer peptidyl nucleoside antibiotics featuring a simplified azetidine-containing moiety

**DOI:** 10.1016/j.synbio.2026.08.010

**Published:** 2026-09-08

**Authors:** Rong Gong, Tianzhu Li, Liwei Zhou, Yuanyuan Liu, Tong Zhang, Yini Qin, Xinyue Zeng, Boyu Jin, Jiahong Wang, Zixin Deng, Xuemin Song, Wenqing Chen

**Affiliations:** a Department of Anesthesiology, Zhongnan Hospital of Wuhan University, School of Pharmaceutical Sciences, Wuhan University, Wuhan, 430071, China; b International Innovation Center for Forest Chemicals and Material, Nanjing Forestry University, Nanjing, 210037, China; c Key Laboratory of Combinatorial Biosynthesis and Drug Discovery, Ministry of Education, Wuhan University, Wuhan, 430071, China

**Keywords:** Nucleoside antibiotics, Chitin synthases, Directed biosynthesis, Azetidine, Inhibitory activity

## Abstract

Fungal and fungal-like pathogen infections pose escalating threats to the global one-health, thus underscoring the urgent need to reverse this trend by accelerating the development of novel antifungal drugs. Polyoxin (POL), a group of structurally related nucleoside antibiotics, is an eco-friendly fungicide targeting fungal cell wall biosynthesis. However, the chemical diversity and therapeutic potential of this antibiotic has remained underexplored. Here, we report the rational design of a series of POL analogs that feature a simplified azetidine-containing moiety, and we have further realized the directed biosynthesis of them using an industrial POL producer as cell factory. Notably, three analogs (POL-A_X_, POL-H_X_, and POL-K_X_), featuring a l-A2CA (l-azetidine-2-carboxylic acid) moiety, exhibit enhanced inhibitory activity against *Candida albicans* and even the oomycete plant pathogen *Phytophthora sojae*. Moreover, systematic molecular docking analyses reveal that the three POL analogs can be better accommodated to the active pocket of the chitin synthases, directly contributing to the improved inhibitory activity of the designer POL analogs. The study provides the basis for the rational access of next-generation agents targeting chitin biosynthesis.

## Graphical abstract

Polyoxin is an eco-friendly fungicide extensively used in agriculture. Here, the authors report directed biosynthesis of a series of polyoxin analogs featuring a simplified azetidine-containing moiety *via* synthetic biology strategies, and three of them show substantially enhanced inhibitory activity against related pathogens. This study establishes a robust synthetic biology platform for the rational development of next-generation drugs targeting chitin biosynthesis.

**Figure:**
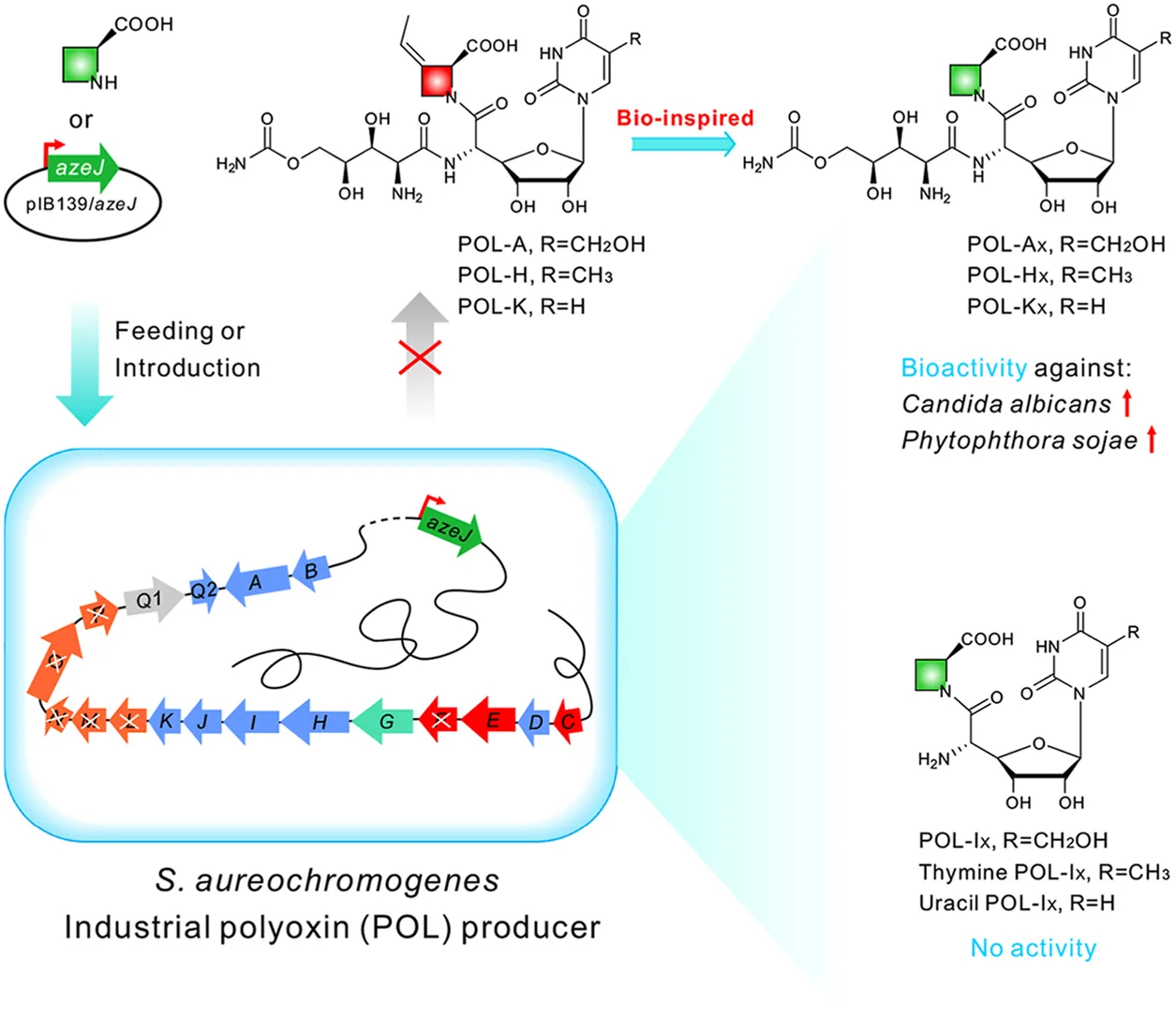

## 1 Introduction

Fungal infections lead to the pressing global challenges [1,2], with fungal and fungal-like (i.e. oomycete) pathogens posing escalating threats to terrestrial ecosystems and agriculture productivity [3]. These pathogens drive widespread species die-offs and even extinctions of wild species, and also imperil global food security by impairing crop yields and agricultural development [4,5]. A pivotal challenge in present development of antifungal drugs lies in achieving selective toxicity that can effectively eradicate fungal pathogens but without causing severe adverse effects for higher eukaryotes [6]. Although the discovery of candidate antifungal molecules has accelerated in the past decades [7], the emergence and spread of drug resistance has remained a continuous rise [8], thereby leading to the limitation of therapeutic options for humans [9]. Consequently, there is an urgent demand for novel agents capable of combating fungal diseases, while alleviating related resistance of antifungal drugs [10].

**Fig. 1:**
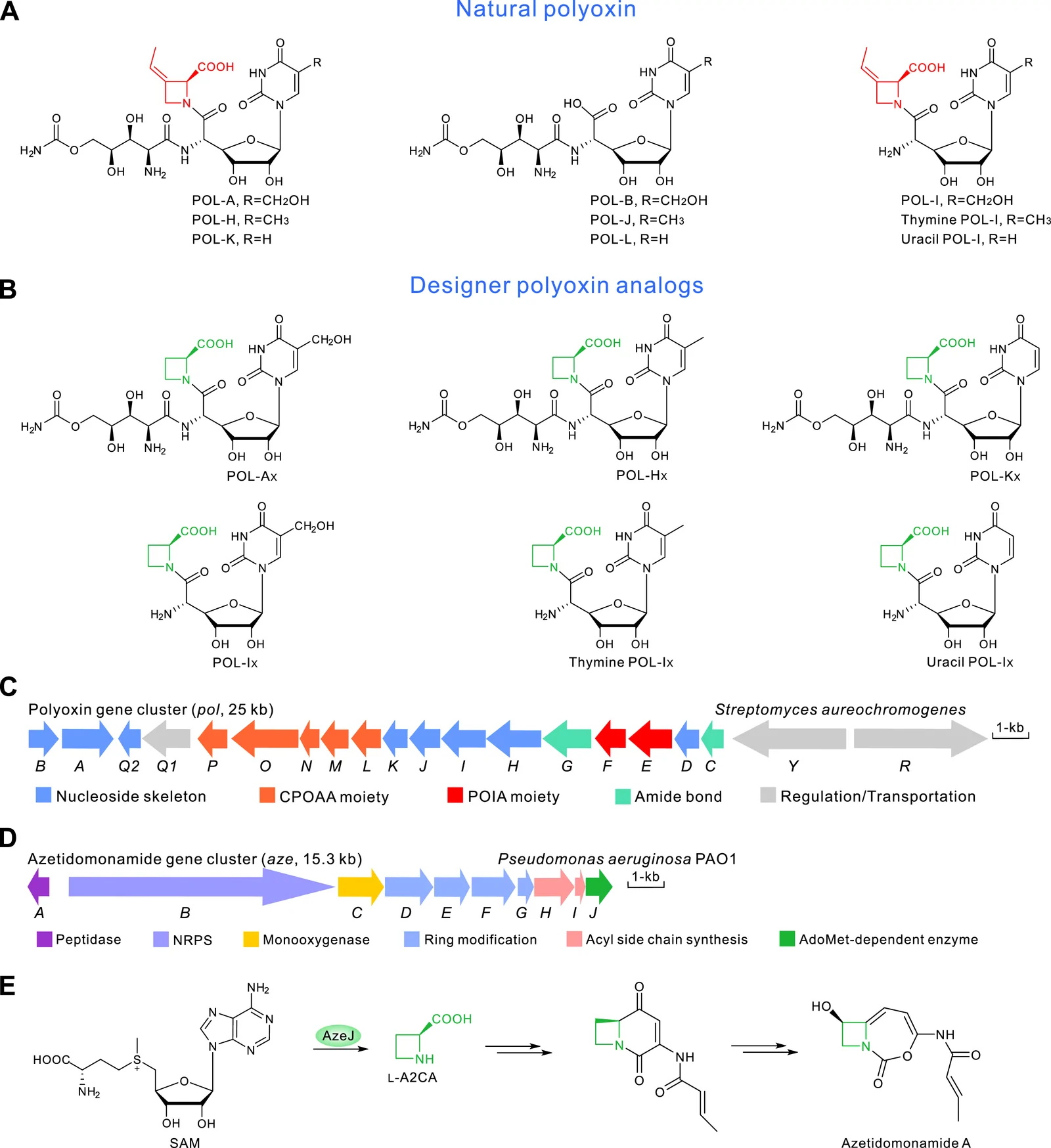
**Structures of relevant antibiotics and genetic organization of the *pol* and *aze* gene clusters.** (A) Structures of natural POL. (B) Structures of the designer POL analogs. (C) Genetic organization of *pol* gene cluster. (D) Genetic organization of *aze* gene cluster. (E) AzeJ is responsible for the biosynthesis of l-azetidine-2-carboxylic acid (l-A2CA) moiety of Azetidomonamide A. SAM, *S*-adenosylmethionine.

The current development of new antifungals is inherently unoptimistic, primarily due to the structural similarities between fungal and host cellular machinery, which increases the risk of off-target toxicity [11]. Of all representative antifungal agents [12,13], nucleoside antibiotics represent an agriculturally relevant group of microbial secondary metabolites [14]. These compounds exhibit potent antifungal activity but with relatively-minimal toxicity to humans, animals, or plants [15], thereof making them as attractive scaffolds for drug development [[16], [17], [18], [19]]. Polyoxin (POL) (Fig. 1A), produced by *Streptomyces aureochromogenes* and *Streptomyces cacaoi* var. *asoensis* [20], represents a group of structurally related peptidyl nucleoside antibiotics [21]. POL consists of a nucleoside skeleton and two non-proteinogenic amino acids designated as polyoximic acid (POIA) and carbamoylpolyoxamic acid (CPOAA) [22]. Mechanistically, POL mimics UDP-*N*-acetylglucosamine (UDP-GlcNAc), a direct substrate for chitin biosynthesis, accordingly, and it acts as powerful competitive inhibitor of chitin synthases (CHSs) [23]. Previous studies confirmed that POL could bind to the substrate-binding pocket of Chs2 in *Candida* *albicans* and Chs1 in *Saccharomyces cerevisiae* [[24], [25], [26]], thereby blocking substrate entry, preventing chain elongation, and exerting antifungal activity. Owing to this powerful inhibitory activity targeting fungal cell wall biosynthesis, POL has been extensively used as an eco-friendly fungicide in agriculture.

Over the last two decades, synthetic biology strategies have been displayed as powerful means to acceleratively expand nature’s chemical diversity [27], and substantial progress has accordingly been achieved in the generation of designer nucleoside antibiotics. Previously, several “unnatural” hybrid nucleoside antibiotics, including POL-N [28], polynik A [29], and Nikkoxin B-G [28,30], have been rationally generated *via* synthetic biology strategies [31]. Particularly, some of these hybrids exhibit stronger inhibitory activity than their natural counterparts, displaying the versatility and prowess of synthetic biology as a robust platform for diversifying and developing antifungal drug leads. Although the core biosynthetic pathway of POL has been largely elucidated in the past decades (Fig. 1C and Fig. S1) [[32], [33], [34]], chemical diversity and therapeutic potential of POL have remained to be further explored.

In this study, we formulate a series of designer POL analogs, which are featured by a simplified azetidine-containing moiety (Fig. 1B), with guidance of structural analysis of chitin synthase, and we further realize the directed synthesis of the designer antibiotics in an industrial POL producer with employment of rational strategies. Notably, we reveal that three analogs (POL-A_X_, POL-H_X_, and POL-K_X_) exhibit significantly enhanced inhibitory activity against the human-pathogenic *C*. *albicans* and the phytopathogenic *Phytophthora* *sojae* compared to their natural counterparts. This study opens the way for future development and rational access of more designer nucleoside antibiotics *via* synthetic biology approaches.

## 2 Results and discussion

### 2.1 Analysis of the *Ca*Chs2–POL complex leads to rational design of the POL analogs featuring a simplified azetidine-containing moiety

**Fig. 2:**
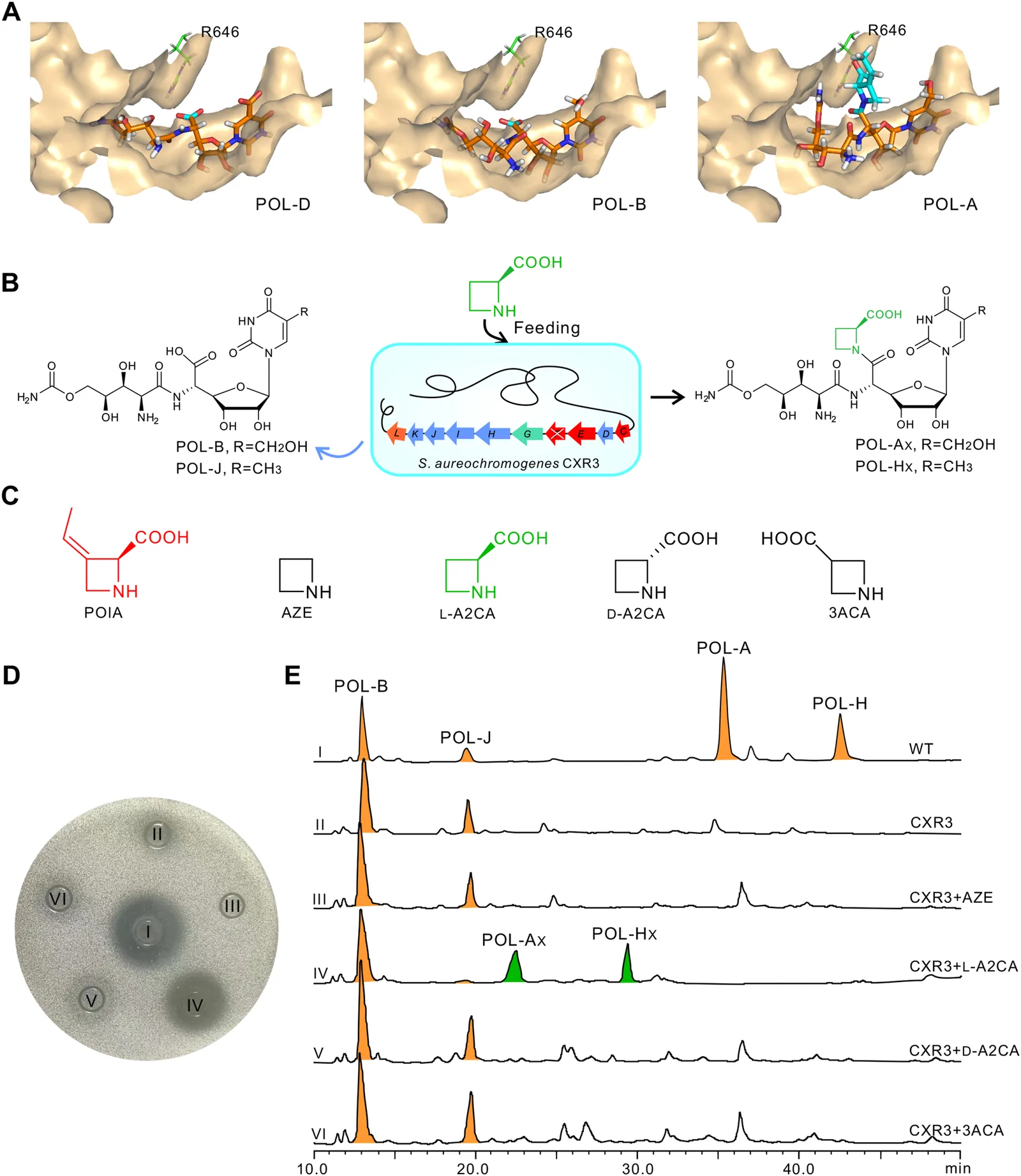
**Rational design and generation of POL-Ax and POL-H_X_ by mutasynthesis strategy.** (A) Molecular docking analysis of *Ca*Chs2 with POL-B or POL-A. The binding surface representation of *Ca*Chs2 with POL-D (left), POL-B (middle), and POL-A (right). (B) Schematic illustration of CXR3 fed with POIA analogs to generate POL-A analogs. (C) Structures of related azetidine-containing compounds. AZE, azetidine; l-A2CA, l-azetidine-2-carboxylic acid; d-A2CA, d-azetidine-2-carboxylic acid; 3ACA, 3-azetidinecarboxylic acid. (D) Bioassay analysis of the metabolites of CXR3 fed with POIA analogs. I, metabolites of the strain *S. aureochromogenes*; II, metabolites of the strain CXR3; III, metabolites of the strain CXR3 fed with AZE; IV, metabolites of the strain CXR3 fed with l-A2CA; V, metabolites of the strain CXR3 fed with d-A2CA; VI, metabolites of the strain CXR3 fed with 3ACA. (E) HPLC analysis (wavelength λ = 260 nm) of the target metabolites produced by the strain *S. aureochromogenes* WT, CXR3, and CXR3 individually fed with AZE, l-A2CA, d-A2CA, or 3ACA.

The complex structure *Ca*Chs2–POL-D (PDB: 7STO) of chitin synthase *Ca*Chs2 from *C*. *albicans* and POL-D (comprising CPOAA and the nucleoside skeleton with a 5-carboxyuracil base) was recently resolved, which provides critical insights into the molecular interactions of chitin synthase and POL [26], and we therefore performed further structure investigation of the *Ca*Chs2–POL-D complex. The results indicated that the CPOAA moiety and nucleoside skeleton of POL-D play pivotal roles in binding with the active sites of the chitin synthase. *S*. *aureochromogenes* produces POL-B (lacking the POIA moiety), POL-A (containing POIA and the nucleoside skeleton with a 5-hydroxymethyluracil base), and POL-H (containing POIA and the nucleoside skeleton with a 5-methyluracil base) as the principal components (Fig. 2). Notably, previous studies have revealed that POL-B exhibits favorable inhibitory activity against *C*. *albicans* compared to POL-A, despite their high structural similarity, indicating that the presence or absence of the POIA moiety critically influences bioactivity [28].

To further investigate the structural basis accounting for these differences, we performed molecular docking to compare the binding modes of *Ca*Chs2 with POL-A and POL-B. Our comparative docking analysis revealed that, compared to POL-B and POL-D, POL-A adopts a more twisted conformation within the *Ca*Chs2 active pocket (Fig. 2A). This conformational distortion is primarily attributed to the rigid, fused-ring architecture of the POIA moiety, which appears to impose steric and geometric restrictions that deviate from the optimal binding geometry observed for POL-B and POL-D. Intriguingly, while this rigidification does not abolish binding, it likely reduces the complementarity between POL-A and the active-site cavity, thereby providing a structural rationale for its diminished antifungal activity against *C*. *albicans* relative to POL-B. These observations suggest that the designer POL analogs with POIA substituted for other simplified azetidine-containing moiety is more likely to harbor reduce conformational strain and enhanced antifungal activity.

### 2.2 Disrupting POIA biosynthesis in *S. aureochromogenes* leads to the engineered production of POL-B

To realize the engineered synthesis of the designer POL-analogs, the biosynthesis of POIA should be disrupted. The genes, *polE* and *polF*, were recently reported to be required for the biosynthesis of POIA, and PolF, a new member of the emerging haem-oxygenase-like diiron oxidase/oxygenase (HDO) superfamily of enzymes, exhibits dual functionality, orchestrating the sequential desaturation and cyclization with l-isoleucine as the initial substrate to construct POIA [32]. Accordingly, we set out to delete this gene in chromosome to disrupt POIA biosynthesis (Fig. S2A and B), and the resultant CXR3 strain was then fermented for metabolites analysis. Bioassay showed that metabolites of the CXR3 strain almost have no inhibition on the indicator fungi *Trichosporon cutaneum* (Fig. S2C), and high-pressure liquid chromatography (HPLC) analysis indicated that the CXR3 sample can generate the target peaks corresponding to those of POL-B and POL-J (Fig. S2D). Further liquid chromatography high-resolution mass spectrometry (LC-HRMS) analysis exhibited that the target peaks of CXR3 is capable of giving rise to distinctive [M+H]^+^ ions at *m/z* 508.1512 (with major fragment ions at *m/z* 490.1417, 447.1355, and 366.1145) and *m/z* 492.1566 (with major fragment ions at *m/z* 474.1299, 431.1401, and 366.1139), which are fully consistent with the expected fragmentation patterns of POL-B and POL-J (Fig. S2E–H). These data demonstrate that deletion of the *polF* gene in *S. aureochromogenes* strain is a feasible way to realize engineered production of POL-B and POL-J, while abolishing POL-A and POL-H biosynthesis.

### 2.3 Directed biosynthesis of POL analogs featuring the simplified azetidine-containing moiety is achieved *via* a mutasynthesis approach

To investigate if related simplified azetidine-containing moiety can be incorporated into the biosynthetic pathway of POL, four azetidine-containing compounds, including azetidine (AZE), l-azetidine-2-carboxylic acid (l-A2CA), d-azetidine-2-carboxylic acid (d-A2CA), and 3-azetidinecarboxylic acid (3ACA), were individually fed into the CXR3 cultures (Fig. 2B and C) for metabolites analysis. Strikingly, bioassay indicated that only the cultures supplemented with l-A2CA restores the antifungal activity against *T. cutaneum* (Fig. 2D), implying that the target new compounds may be generated as a consequence. Further HPLC analysis indicated that the sample from CXR3, fed with l-A2CA, was capable of producing two new metabolites, designated as POL-A_X_ and POL-H_X_ (corresponding to POL-A and POL-H, Fig. 2E). LC-HRMS analysis showed that POL-A_X_ generated a characteristic [M+H]^+^ ion at *m/z* 591.1880, with MS/MS fragmentation ions at *m/z* 573.1304, 529.3221, 490.1221, 472.1354, 449.1375 and 400.9432, which are fully consistent with the fragmentation pattern of the predicted POL-A analog containing l-A2CA (Figs. S3 and S4A). Similarly, POL-H_X_ displayed a characteristic [M+H]^+^ ion at *m/z* 575.1931, with MS/MS fragmentation ions at *m/z* 557.1573, 513.9924, 474.0939, 456.0414, 449.0907 and 385.1327, which are well matched to the fragmentation pattern of the POL-H analog containing l-A2CA (Figs. S3 and S4B).

In addition, LC-HRMS analysis indicated that the CXR3 metabolites (with feeding of d-A2CA**)** can also generate new characteristic peaks of [M+H]^+^ ions, albeit with much lower-abundance, at *m/z* 591.1879 and *m/z* 575.1937, correspondingly consistent with the predicted analogs POL-A_Y_ and POL-H_Y_ (Figs. S3 and S4C, D). Likewise, CXR3, fed with 3ACA, can yield trace levels of two metabolites as well with individual [M+H]^+^ ions at *m/z* 591.1881 and *m/z* 575.1929, corresponding to those of the predicted analogs POL-A_Z_ and POL-H_Z_ (Figs. S3 and S4E, F). Taken together, these results indicate that l-A2CA is capable of being efficiently recognized and incorporated into the designer POL analogs, whereas d-A2CA and 3ACA can merely be incorporated and identified at trace levels.

To further expand chemical diversity of POL analogs with a simplified azetidine-containing moiety, we consequently inactivated the *polB* gene (encoding a C-5 methylase of the nucleoside skeleton) in CXR3 (Fig. S5A and B) [35], yielding the resultant strain CXR6. HPLC analysis indicated that the fermentation sample of this strain generates a new metabolite peak (Fig. S5C), and further LC-HRMS analysis revealed that it generates a distinctive [M+H]^+^ ion at *m/z* 478.1414, with major fragment ions at *m/z* 460.1667, 417.0833, 366.1667, and 288.0833, which are well conformed with the expected fragmentation pattern of POL-L. We then supplemented the CXR6 cultures with the four azetidine-containing compounds (AZE, l-A2CA, d-A2CA, and 3ACA). Similarly, bioassays indicated that the cultures only supplemented with l-A2CA regain the bioactivity against the indicator fungi *T. cutaneum* (Fig. S6A). HPLC analysis showed that a new metabolite peak (designated as POL-K_X_) is generated (Fig. S6B), and further LC-HRMS analysis exhibited that this metabolite can produce a characteristic [M+H]^+^ ion at *m/z* 561.1777, with MS/MS fragmentation ions at *m/z* 543.2169, 500.2399, 460.1425, 449.2496, 442.0845 and 371.1164, fully matched to the theoretical pattern of the predicted POL-K analog containing l-A2CA (Figs. S7 and S8A). Furthermore, LC-HRMS analysis also showed that trace levels of additional analogs POL-K_Y_ and POL-K_Z_ can be detected as well in the CXR6 cultures with corresponding supplementation of d-A2CA and 3ACA (Figs. S7 and S8A,B). Collectively, these results further confirm that the POL biosynthetic machinery harbors certain flexibility against POIA-related substrates, but with considerable preference to l-A2CA.

### 2.4 Production of the target azetidine-containing POL analogs is realized *via* introduction of l-A2CA pathway

**Fig. 3:**
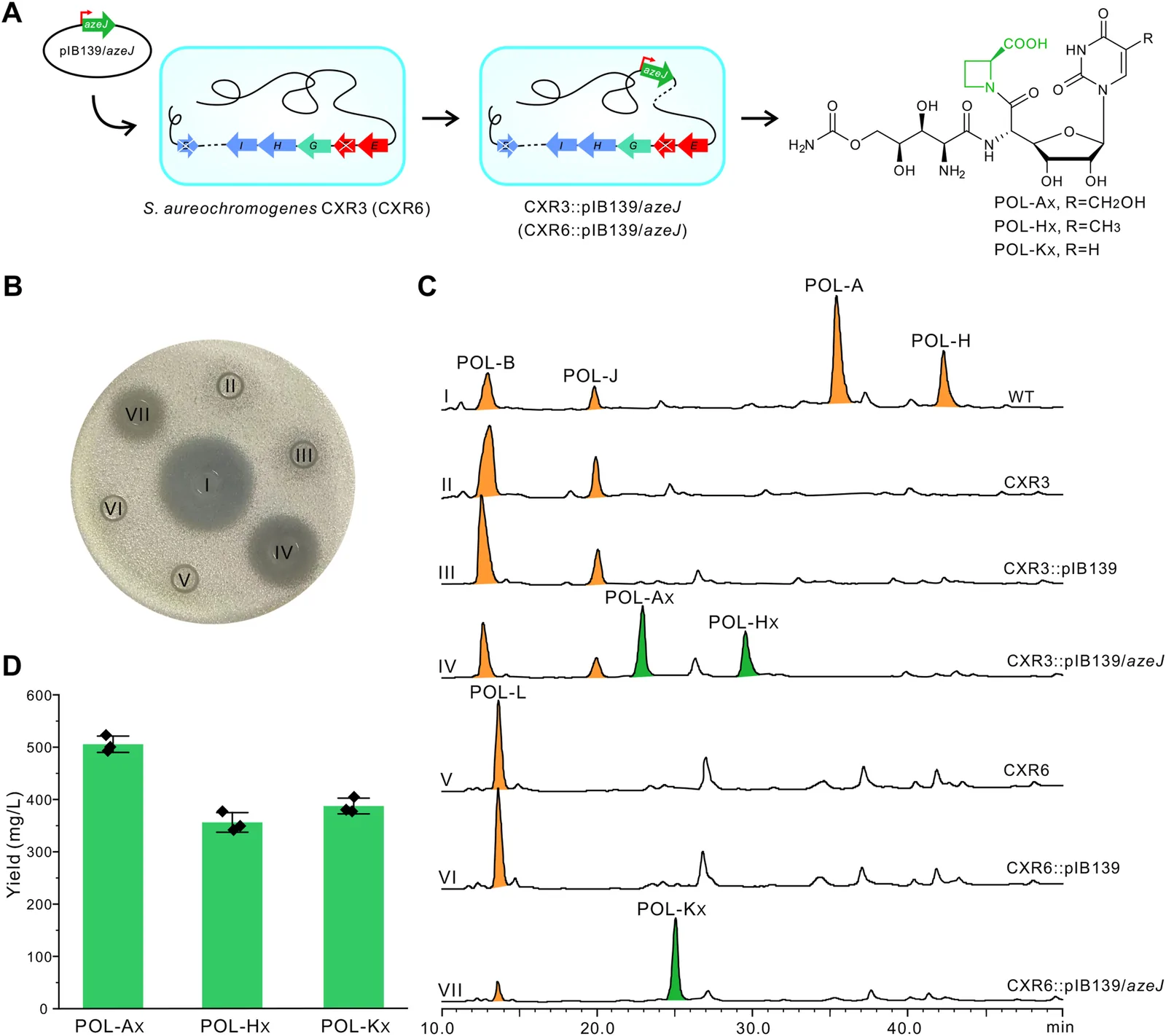
**Rational production of POL-Ax, POL-H_X_, and POL-Kx by related strains.** (A) Schematic illustration of the combinatorial biosynthesis strategy for the generation of POL-A_X_, POL-H_X_, and POL-K_X_. (B) Bioassay analysis of the metabolites of related strains using *T. cutaneum* as indicator strain. I, metabolites of the strain *S. aureochromogenes*; II, metabolites of the strain CXR3; III, metabolites of the recombinant CXR3:pIB139; IV, metabolites of the recombinant CXR3:pIB139/*azeJ*; V, metabolites of the strain CXR6; VI, metabolites of the recombinant CXR6:pIB139; VII, metabolites of the recombinant CXR6:pIB139/*azeJ*. (C) HPLC analysis of the target metabolites produced by the related strains. (D) The yield of POL-A_X_, POL-H_X_, and POL-K_X_ separately produced by the recombinants CXR3:pIB139/*azeJ* and CXR6:pIB139/*azeJ*.

To eliminate the demand for exogenous l-A2CA supplementation, we attempted to engineer the POL cell factory to produce the target POL analogs *via* introducing the l-A2CA biosynthetic pathway [36]. Recently, the *azeJ* gene (coding for an AdoMet-dependent enzyme) from *Pseudomonas aeruginosa* PAO1 was functionally determined for l-A2CA biosynthesis (Fig. 1D, E and Fig. S9) [37,38], and we then cloned the code-optimized *azeJ* (based on codon usage preferences of *Streptomyces*, Table S10) into the integrative plasmid pIB139 (driven by the promoter ermE*). The plasmid pIB139/*azeJ* was then conjugated into the CXR3 and CXR6 strains (Fig. 3A), and the empty vector pIB139 was also introduced into the same strain as negative control. Then, the resulting recombinants were subsequently fermented for further metabolites analysis. Bioassay indicated that metabolites of both recombinants CXR3:pIB139/*azeJ* and CXR6:pIB139/*azeJ* restores the antifungal activity against the indicator fungi *T. cutaneum* (Fig. 3B), and further HPLC analysis showed that three new metabolites are accordingly produced by the recombinants CXR3:pIB139/*azeJ* and CXR6:pIB139/*azeJ*, while the strains, involving *S. aureochromogenes*, CXR3, CXR3:pIB139, CXR6, and CXR6:pIB139, lack the capacity to produce the target compounds (Fig. 3C). Further LC-HRMS analysis showed that the sample of CXR3:pIB139/*azeJ* generates a characteristic [M+H]^+^ ion at *m/z* 591.1877, and a characteristic [M+H]^+^ ion at *m/z* 575.1932. Similarly, the recombinant strain CXR6:pIB139/*azeJ* is able to generate a characteristic [M+H]^+^ ion at *m/z* 561.1785. More than that, the major fragments of related metabolites, as indicated by LC-MS/MS analysis, are correspondingly consistent with the theoretical fragmentation patterns of the compound POL-A_X_, POL-H_X_, and POL-K_X_ (Fig. S10).

To unequivocally confirm the chemical structures of POL-A_X_, POL-H_X_, and POL-K_X_, we therefore purified and subjected these compounds for nuclear magnetic resonance (NMR) spectroscopy analysis (Figs. S11–S25). As expected, the ^1^H and ^13^C NMR spectra of the three analogs exhibited overall similarity, except for the C7 position signals at H_δ_ 4.25 (s, 2H) and C_δ_ (56.46 ppm) of compound POL-A_X_, signals at H_δ_ 1.77 (d, *J* = 5.1 Hz, 3H) and C_δ_ (11.45 ppm) of compound POL-H_X_. In addition, significant differences were also observed at the C5 position, signal at C_δ_ (113.48 ppm) of compound POL-A_X_, signal at C_δ_ (111.27 ppm) of compound POL-H_X_, while signals at H_δ_ 5.87 (d, *J* = 8.1 Hz, 1H) and C_δ_ (102.19 ppm) of compound POL-K_X_. These chemical shift patterns correspond to hydroxymethyl (POL-A_X_), methyl (POL-H_X_), and hydrogen (POL-K_X_) substituents at the C5 position, which also further confirmed by the two-dimensional (2D) NMR analysis. Further analysis showed that the yields of POL-A_X_ and POL-H_X_ produced by recombinant CXR3:pIB139/*azeJ* independently reach 505.56 mg L^−1^ and 356.13 mg L^−1^, while the recombinant CXR6:pIB139/*azeJ* is capable of accumulating POL-K_X_ with a yield of 386.08 mg L^−1^ (Fig. 3D). Additionally, the strain *S. aureochromogenes* produced POL-A and POL-H with a yield of 617.13 mg L^−1^ and 466.41 mg L^−1^, while POL-B and POL-J with a yield of 89.95 mg L^−1^ and 26.00 mg L^−1^. Taken together, these results demonstrate that the designer metabolites, POL-A_X_, POL-H_X_, and POL-K_X_, is efficiently produced in an engineered POL producer *via* a synthetic biology strategy.

We next set out to extend this strategy by incorporating (2*S*,4*R*)-4-methylazetidine-2-carboxylic acid (MAZ), a methylated derivative of l-A2CA, into the POL scaffold with an aim to further produce new designer POL analogs. The *vioG* gene, which codes for a protein catalyzing the methylation of l-A2CA to form MAZ during vioprolides biosynthesis (Fig. S26A) [39,40], was then cloned into the plasmid pIB139*/azeJ,* and the dual-expression plasmid pIB139/*azeJ*&*vioG* was introduced into the strain CXR6. Subsequently, the resulting recombinant CXR6:pIB139/*azeJ*&*vioG* was fermented for metabolite analysis. HPLC analysis indicated that the recombinant metabolites cannot generate any new peaks corresponding to the POL derivatives containing MAZ (Fig. S26B). However, we found that the recombinant holds the capability of MAZ production, as confirmed by LC-HRMS analysis (Fig. S27). These data demonstrate that the POL biosynthetic machinery cannot recognize MAZ as the potential substrate to biosynthesize the target compounds. We infer that the bulky methyl group at the C3 position of the azetidine ring may create severe steric hindrance, preventing the ligase responsible for the assembly of POIA and nucleoside skeleton in POL biosynthesis from recognizing MAZ as a competent substrate, which highlighting the substrate specificity of key enzyme in the POL biosynthetic pathway.

### 2.5 Expanding chemical diversity of POL-I analogs is achieved by further engineering of the CXR3/CXR6 strain

To further expand the structural diversity of azetidine-containing POL analogs, we conducted targeted-deletion of the genes *polLMNOP*, which encode enzymes responsible for the biosynthesis of CPOAA in the CXR3/CXR6 strain, correspondingly rendering the strains CPO1 and CPO2 (Figs. S28A,B and S29A,B), and the strains were subsequently fermented for metabolites analysis. HPLC analysis showed that the sample from the CPO1 strain is capable of producing the target metabolites POL-C and thymine POL-C, and CPO2 is able to biosynthesize uracil POL-C (Figs. S28C and S29C). The identities of related metabolites were further confirmed by LC-HRMS analysis (Figs. S28D-G and S29D,E).

**Fig. 4:**
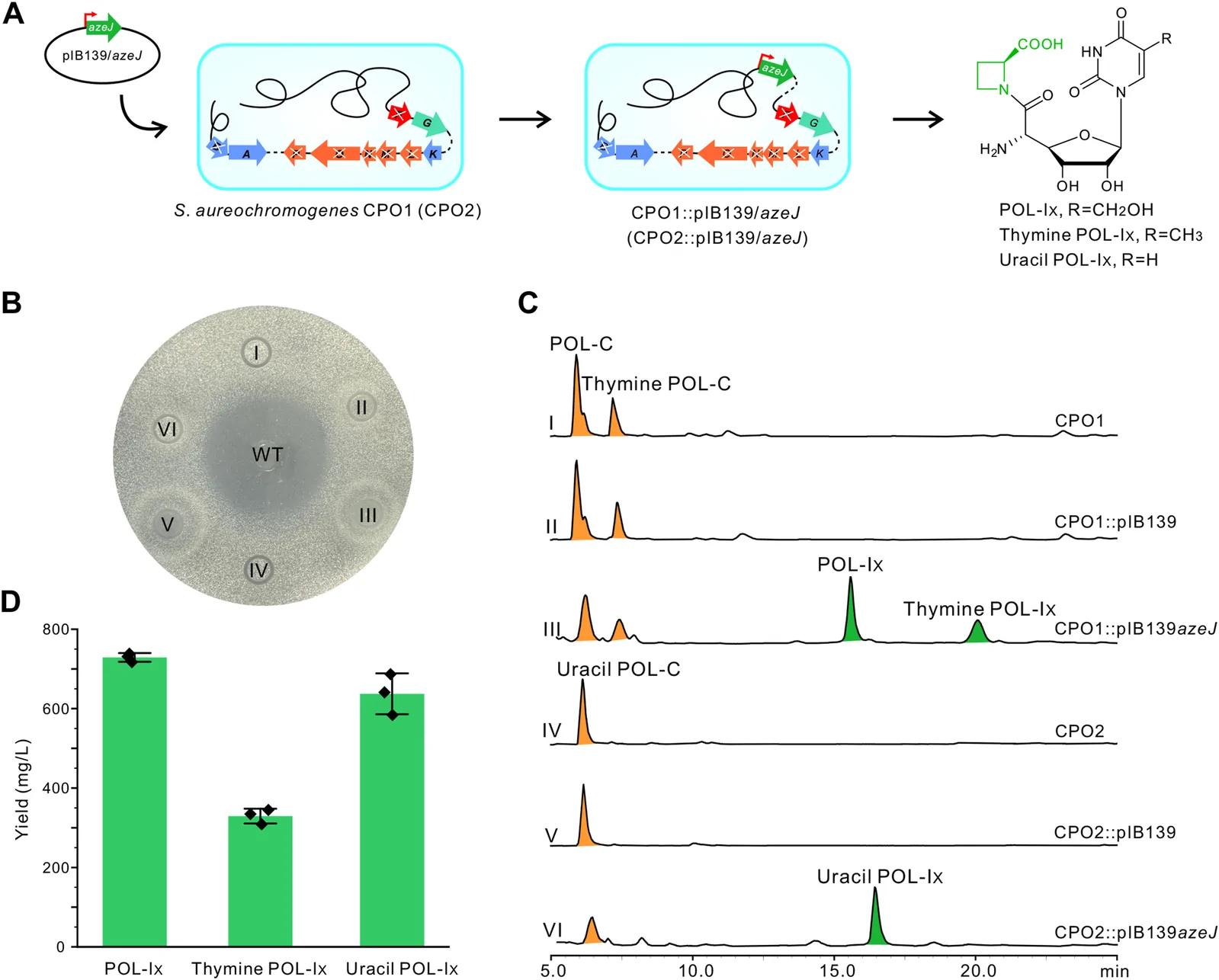
**Rational production of POL-Ix**, **thymine POL-Ix**, **and uracil POL-I_X_ by related strains.** (A) Schematic illustration of the generation of POL-I_X_, thymine POL-_X_, and uracil POL-_X_ by counterpart strains. (B) Bioassay analysis of the metabolites of CPO1:pIB139/*azeJ* and CPO2:pIB139/*azeJ* using *T. cutaneum* as indicator strain. WT, metabolites of the strain *S. aureochromogenes*; I, metabolites of the strain CPO1; II, metabolites of the recombinant CPO1:pIB139; III, metabolites of the recombinant CPO1:pIB139/*azeJ*; IV, metabolites of the strain CPO2; V, metabolites of the recombinant CPO2:pIB139; VI, metabolites of the recombinant CPO2:pIB139/*azeJ*. (C) HPLC analysis of the target metabolites produced by the related strains. As indicated, the recombinant CPO1:pIB139/*azeJ* produced POL-Ix and thymine POL-I_X_, then the recombinant CPO2:pIB139/*azeJ* generated the uracil POL-I_X_. (D) The yield of POL-I_X_, thymine POL-I_X_, and uracil POL-I_X_ separately produced by the recombinants CPO1:pIB139/*azeJ* and CPO2:pIB139/*azeJ*.

To endogenously provide the l-A2CA building block and explore whether the polyoxin biosynthetic machinery could accept this substrate for scaffold modification. Next, the plasmid pIB139/*azeJ* was individually introduced into the strains CPO1 and CPO2, generating the counterpart recombinants CPO1:pIB139/*azeJ* and CPO2:pIB139/*azeJ* (Fig. 4A), and these strains were fermented as well for metabolites analysis. Bioassays showed that metabolites of these recombinants are not conferred with capability of antifungal activity against *T. cutaneum* (Fig. 4B), and further HPLC analysis indicated that the fermentation sample of CPO1:pIB139/*azeJ* is able to generate new metabolites, named as POL-I_X_ and thymine POL-I_X_. Moreover, a new metabolite uracil POL-I_X_, as indicated by HPLC analysis, can also be detected from the sample of CPO2:pIB139/*azeJ* (Fig. 4C). Further LC-HRMS analysis showed that POL-I_X_ exhibits a characteristic [M+H]^+^ ion at *m/z* 401.1288 (with major fragments at *m/z* 382.6596, 258.9303, 240.9316, 223.9844 and 156.6463). Likewise, the identities of thymine POL-I_X_ and uracil POL-I_X_ were also determined by LC-HRMS analysis (Fig. S30).

To further confirmed the identities of the three compounds, they were further characterized by NMR spectroscopy analysis, and the results indicated that the signals of nucleoside skeleton were highly consistent with those of known POL-C related components [41]. The ^1^H and ^13^C NMR spectra of POL-I_X_, thymine POL-I_X_, and uracil POL-I_X_ were largely similar, with key differences observed at C5 and C7 positions. Specifically, POL-I_X_ displayed signals at H_δ_ 4.30 (m, 1H) and C_δ_ (56.47 ppm), indicative of a hydroxymethyl group at C7. In contrast, thymine POL-I_X_ showed signals at 1.83 (d, *J* = 1.2 Hz, 3H) and C_δ_ (11.39 ppm), corresponding to a methyl group. Furthermore, distinct signals were observed at the C5 position, a carbon signal at C_δ_ (113.29 ppm) for POL-I_X_, signal at C_δ_ (111.15 ppm) of thymine POL-I_X_, while signals at H_δ_ 5.86 (d, *J* = 8.1 Hz, 1H) and C_δ_ (102.08 ppm) of uracil POL-I_X_ (Figs. S31–45). Further analysis revealed that recombinants CPO1:pIB139/*azeJ* and CPO2:pIB139/*azeJ* individually produced POL-I_X_, thymine POL-I_X_, and uracil POL-I_X_ attain 729.01 mg L^−1^, 329.34 mg L^−1^, and 637.31 mg L^−1^ (Fig. 4D). The above results indicates that we have expanded the chemical diversity of POL by further engineering of the CXR3/CXR6 strain.

### 2.6 POL-A analogs indicate enhanced bioactivity against *Candida albicans* and *Phytophthora sojae*

As POL exhibits variable bioactivities against counterpart pathogens [28], and we therefore systematically test the designer POL analogs by determining their minimal inhibitory concentration (MIC) against six representative pathogens, including human-pathogenic fungi, plant-pathogenic fungi, and the oomycete pathogen *P*. *sojae*.

**Fig. 5:**
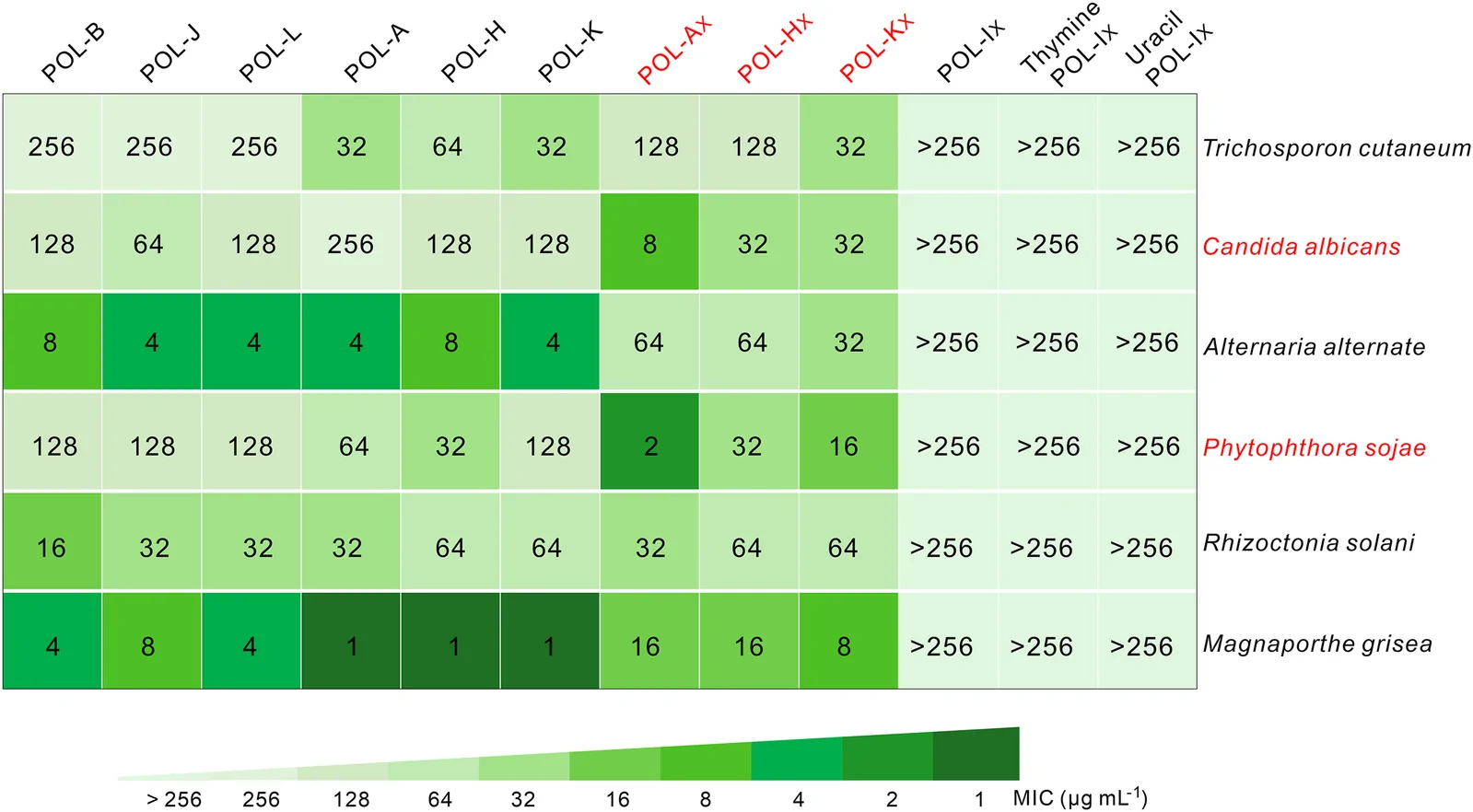
**MIC analysis of natural POLs and related POL analogs.***Trichosporon cutaneum*, *Candida albicans*, *Alternaria alternate*, *Phytophthora sojae*, *Rhizoctonia solani*, and *Magnaporthe grisea* were selected as the indicator strains. The different shades of green color represent MIC of natural POLs and POL analogs (twofold dilutions ranging from 1 to 256 μg mL^−1^). MIC, minimal inhibitory concentration.

As indicated by bioactivity assays, the novel POL analogs, relative to natural POL, display differential antifungal profiles. Of them, POL-A_X_, POL-H_X_, and POL-K_X_, showed relatively weaker inhibitory activity against *Alternaria alternate* and *Magnaporthe grisea*. Moreover, the three designer antibiotics displayed comparable inhibitory activity against *Rhizoctonia solani*. Notably, the three POL analogs (POL-A_X_, POL-H_X_, and POL-K_X_) exhibited significantly enhanced activity against *C*. *albicans* and *P*. *sojae* compared to their natural counterparts (Fig. 5). Altogether, these data demonstrate that incorporation of the simplified azetidine-containing moiety is an effective strategy to improve the antifungal efficacy of POL analogs toward *C*. *albicans* and *P*. *sojae*.

### 2.7 Proposed molecular basis for the enhanced inhibitory activity of POL-A analogs

To gain insight into the potential basis for the improved inhibitory activity of POL-A_X_ against *C. albicans*, we performed molecular docking of the chitin synthase *Ca*Chs2 with POL (Fig. S46). The results suggest that all compounds bind to the *Ca*Chs2 active pocket *via* the uridine moiety, which is similar to the binding mode of the natural substrate UDP-GlcNAc. Significantly, POL-I_X_, thymine POL-I_X_, and uracil POL-I_X_, which lack the CPOAA moiety, exhibit apparently reduced interactions with the conserved Q_643_R_644_XR_646_W_647_ motif, a region critical for UDP-GlcNAc binding, acceptor recognition, and catalysis, and thus POL-I and its analogs consequently abolish the antifungal activity.

**Fig. 6:**
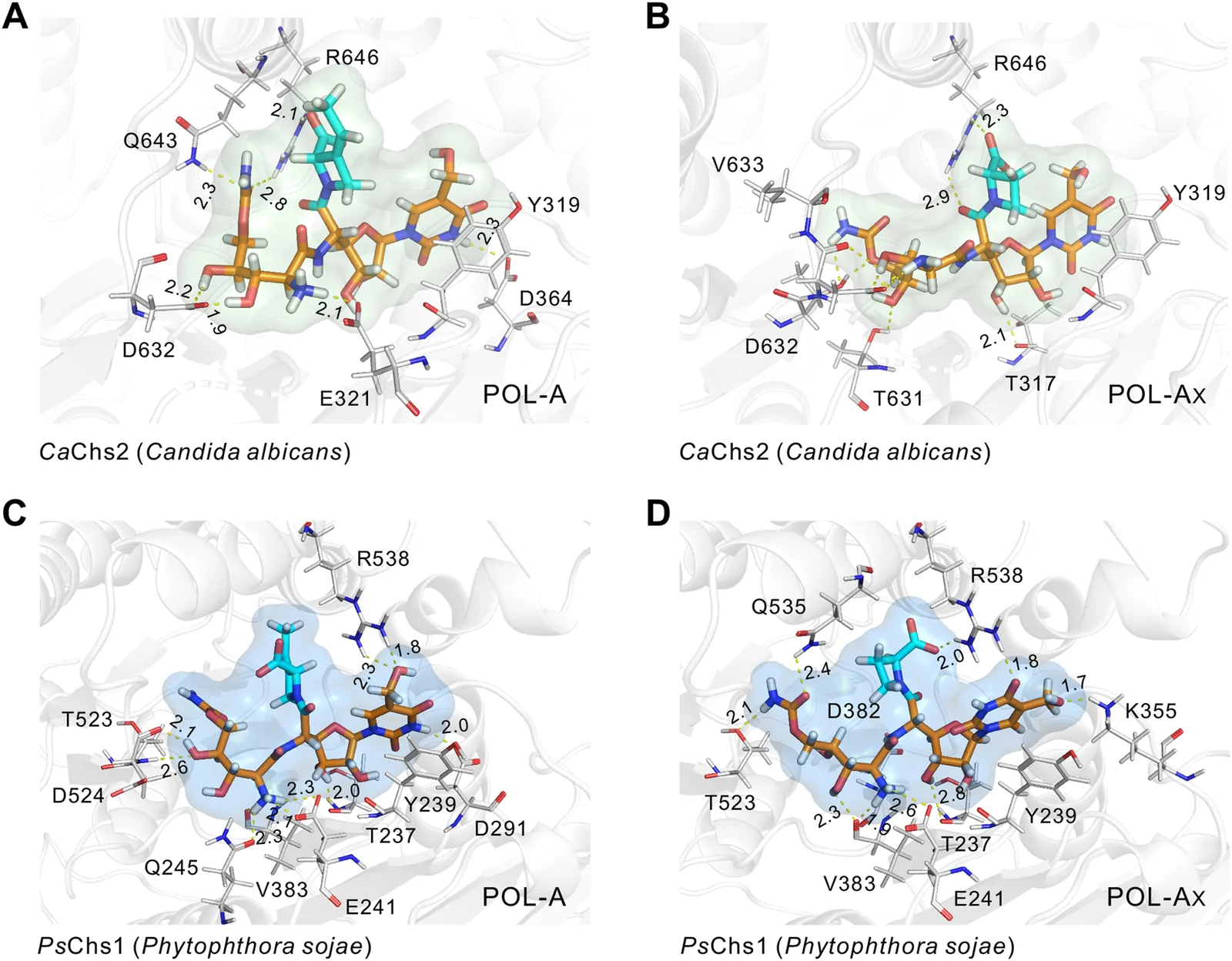
**Representation docking models of *Ca*Chs2 and *Ps*Chs1 with POL-A and POL-A_X_.** (A) Detailed interactions between *Ca*Chs2 and POL-A. (B) Detailed interactions between *Ca*Chs2 and POL-A_X_. (C) Detailed interactions between *Ps*Chs1 and POL-A. (D) Detailed interactions between *Ps*Chs1 and POL-A_X_. Compared to POL-A, POL-A_X_ adapt the irregular shape of the pocket at the greatest extent in *Ca*Chs2 and *Ps*Chs1. The hydrogen bonds are indicated by yellow dashed lines.

In contrast, the CPOAA moiety of POL-A, POL-B, and POL-A_X_ can form extensive interactions with the Q_643_R_644_XR_646_W_647_ motif. Moreover, comparative analysis of the binding modes of *Ca*Chs2 with POL-A and POL-A_X_ revealed that CPOAA occupies the location overlapped with the GlcNAc moiety of UDP-GlcNAc. Specifically, the residue R646 plays a key role by forming interactions with the carboxyl group of azetidine-related moieties in both POL-A and POL-A_X_. Remarkably, the residue Q643 can form a hydrogen bond with 8‴-carboxyl group of POL-A, and the twist conformation induced by its rigid exocyclic C=C bond partially compromises binding affinity. Comparatively, the CPOAA moiety of POL-A_X_ makes additional stabilization-contacts with the residues T631, D632, and V633 (Fig. 6A and B). Taken together, these predicted intermolecular interactions are consistent with the observed changes in inhibitory potency of the tested POL analogs.

Furthermore, *Ps*Chs1 (PDB: 7WJO, chitin synthase from *P. sojae*) with POL-A, POL-B, or POL-A_X_ [42], as indicated by molecular docking analysis, also employs similar model to that of UDP-GlcNAc (Fig. S47 and Fig. 6C and D), while POL-A_X_ can effectively adapt to the irregular space of the active pocket, which is consistent with the observations from *Ca*Chs2, thereof leading to the stronger interactions with key residues and accordingly enabling the enhanced bioactivity against *P. sojae*.

## 3 Conclusions

Although POLs are known to act as competitive inhibitors of chitin synthase by mimicking UDP-GlcNAc, the precise role of the POIA moiety has remained unclear. Our study demonstrates that the rigid POIA moiety imposes conformational strain that compromises binding complementarity in some chitin synthase, a previously unrecognized structural liability. Additionally, the successful incorporation of l-A2CA into the POL scaffold *via* synthetic biology strategies confirms that the biosynthetic machinery can accommodate simplified azetidine-containing moieties. Notably, POL-A_X_, POL-H_X_, and POL-K_X_ exhibit enhanced activity against both the human pathogen *C. albicans* and the oomycete *P. sojae*. Given that current polyoxin-based agrochemicals show limited activity against *Phytophthora* diseases, our azetidine-containing analogs could potentially fill this gap in crop protection.

In summary, we have formulated a series of POL analogs which are highlighted by a simplified azetidine-containing moiety, and we have further realized the engineered production of the six designer compounds *via* a combination of rational strategies. Remarkably, we have found that three designer POL analogs (POL-A_X_, POL-H_X_, and POL-K_X_) exhibit significantly improved inhibitory activity against *C. albicans* and *P. sojae*. Moreover, we have explored molecular docking and accounted for the molecular mechanisms underlying the enhanced bioactivity of related designer POL analogs. We anticipate that the present work will provides a promising and robust synthetic biology platform for future development of more related designer nucleoside antibiotics.

## 4 Methods

### 4.1 Enzymes, chemicals, and reagents

All restriction enzymes, DNA ligases, and polymerases used in this study were purchased from New England Biolabs. Chemicals and reagents were obtained from Sigma-Aldrich, Thermo Scientific, OMEGA, or J&K Scientific.

### 4.2 Strains and culture conditions

*E. coli* strains were cultured in Luria-Bertani (LB) medium (5.0 g L^−1^ yeast extract, 10.0 g L^−1^ tryptone, 5.0 g L^−1^ NaCl) at 37 °C. *Streptomyces* strains were grown on Mannitol-Soy (MS) agar plates (20.0 g L^−1^ soy flour, 20.0 g L^−1^ mannitol, 20.0 g L^−1^ agar, pH 7.0) at 30 °C. The indicator strain *T. cutaneum* and *C. albicans* were grown in potato dextrose agar (PDA) medium (200.0 g L^−1^ potato, 20.0 g L^−1^ sucrose, 10.0 g L^−1^ agar, pH 7.0) at 30 °C, then *A. alternate*, *P. sojae*, *P. sasakii*, and *M. grisea* were grown in yeast extract-malt extract (YEME) medium (3.0 g L^−1^ yeast extract, 5.0 g L^−1^ peptone, 3.0 g L^−1^ maltose syrup, 10.0 g L^−1^ glucose, 340.0 g L^−1^ sucrose) at 30 °C. Strains and plasmids used in this study are described in Supplementary Table S1, and primers are listed in Supplementary Table S2.

### 4.3 Construction of *S. aureochromogenes* related mutants

For construction *S. aureochromogenes* related mutants, a strategy similar to that described previously [21,43]. Taking CXR3 as an example, the polF-LF/R and polF-RF/R as primers and pOJ446 as the starter vector were used in this study (Table S2). For construction of the strain, the disruption vector pJTU4723 (Table S1) was constructed based on methods similar to that described previously by Chen et al. [28], and the vector was introduced into *S. aureochromogenes via* conjugation. Then, the *tsr* was replaced to construct the in-frame deletion strain CXR3, which further confirmed by sequencing analysis (Table S3).

### 4.4 Construction of CXR3::pIB139/*azeJ* and related strains

The code-optimized *azeJ* gene (Table S10) was cloned to the NdeI-EcoRI sites of pIB139 (driven by the promoter ermE*), and the plasmid pIB139/*azeJ* was confirmed by sequencing analysis. For construction of pIB139/*azeJ*&*vioG*, the *vioG* gene (with the promoter ermE*) was cloned to the EcoRI sites of pIB139/*azeJ,* and the plasmid pIB139/*azeJ*&*vioG* was confirmed by sequencing analysis. Then, the plasmid pIB139, pIB139/*azeJ*, and pIB139/*azeJ*&*vioG* were independently transformed into *E. coli* ET12567/pUZ8002 for the following conjugation into the CXR3 and related strains.

### 4.5 Metabolites analysis of related strains

For metabolites analysis, *S. aureochromogenes*, CXR3, CXR6, CPO1, CPO2, and related strains were inoculated into 5 mL of Tryptic-Soy-Broth (TSB) medium (30.0 g L^−1^ tryptone soya broth, pH 7.0) and cultured at 30 °C for 24 h. After that, the seed cultures (2%, V/V) were transferred to 70 mL fermentation medium (20.0 g L^−1^ soy powder, 15.0 g L^−1^ corn powder, 10.0 g L^−1^ glucose, 4.0 g L^−1^ yeast extract, 3.0 g L^−1^ CaCO_3_, 1.0 g L^−1^ KH_2_PO_4_ and 1.0 g L^−1^ NaCl) and fermented at 30 °C for 3 d. The fermentation broth was processed by adding oxalic acid till pH 3.0, then the culture was purified by Dowex 50 W × 8(H^+^), after concentration, the culture supernatant was filtered through 0.22 μm microporous membrane for HPLC or LC-HRMS analysis.

HPLC analysis was performed using Shimadzu LC-20AT equipped with a C18 column (Inertsil ODS-3, 5 μm, 4.6 × 250 mm) with an elution gradient of 10%–45% solvent B (water-acetonitrile = 6:4, 10 mM L^−1^ sodium 1-hexanesulfonate and 0.2% acetic acid): solvent A (10 mM L^−1^ sodium 1-heptanesulfonate and 0.2% acetic acid) over 30 min, and the status (at 30 min) was maintained till 50 min. After that, solvent B was dropped to 10% at 51 min, and then this status was kept for another 30 min. The elution was monitored at 260 nm at 0.5 mL min^−1^ with a DAD detector.

LC-HRMS analysis was carried out on a Thermo Fisher Scientific ESI-LTQ Orbitrap (Scientific Inc.) instrument controlled by Xcalibur in the positive mode. The parameters for HRMS analysis are listed as follows: a capillary temperature of 275 °C and a capillary voltage of 35 V, and the error in this work is 10 ppm. The elution gradient of 5% solvent B (methanol) was kept for 5 min at a flow rate of 0.3 mL min^−1^, and then 5%–50% solvent B: solvent A (0.1% aqueous formic acid) over 10 min, and the status (at 10 min) was maintained till 25 min, after that, solvent B was dropped to 5% at 35 min, and then this status was kept for another 5 min.

### 4.6 Compounds purification and yield determination

For the purification of target product, 1 L fermentation broths were processed as described above, then were separated using Shimadzu LC-20AT equipped with Zorba SB-C18 column (Agilent, 5 μm, 9.4 × 250 mm) with a linear gradient of 15% phase B (phase A, aqueous 0.2% TFA; phase B, MeOH) over 30 min at flow rate of 3 mL min^−1^, and the elution was monitored by an ADA detector at 260 nm. After that, further purified by C18 column (Inertsil ODS-3, 5 μm, 4.6 × 250 mm) with an elution gradient of 5% phase B (phase A, 0.2% aqueous trifluoroacetic acid; phase B, methanol) over 25 min at flow rate of 0.5 mL min^−1^.

For yield determination of the target compounds (POL-B, POL-J, POL-L, POL-A, POL-H, POL-K, POL-A_X_, POL-H_X_, POL-K_X_, POL-I_X_, thymine POL-I_X_, and uracil POL-I_X_), standard curves were constructed using the purified compounds at concentrations ranging from 50 to 2000 μg mL^−1^. Each recombinant strain was fermented in triplicate under the same conditions as described above. The yields were calculated by interpolating the peak areas of the corresponding compounds in the fermentation samples against the respective standard curves. All measurements were performed by HPLC with detection at 260 nm.

### 4.7 Bioassay and MIC analysis of the target antibiotics

For bioassay, the indicator strain *T*. *cutaneum* was cultured in TSB medium at 30 °C for 15 h, then mixed into molten PDA medium. Fermentation broth (15 μL) was added into the oxford cup placed on the agar surface were then kept at 4 °C for 2 h to allow the diffusion of metabolites. Subsequently, the bioassay plate was cultivated at 30 °C for 20 h to observe the antifungal zone.

For the determination of the minimal inhibitory concentration (MIC), twofold dilutions ranging from 1 to 256 μg mL^−1^ were used for the tester fungal strains. For yeast strains, 20 μL of each antibiotic solution was added into 180 μL of YEME medium in 96-well plates, and 5 × 10^−2^ yeast CFU were added. Growth was recorded after 20 h incubation at 30 °C. For filamentous fungi, 20 μL of antibiotic solutions were added to 200 μL PDA agar in 96-well plates. The plates were then kept at 4 °C for 2 h to allow the diffusion of antibiotics. The fungi were then inoculated on the surface of the agar and incubated at 30 °C for 20–48 h [30].

### 4.8 Molecular docking

Molecular docking calculations were performed using the CDOCKER (Dock Ligands) module within the BIOVIA Discovery Studio 4.1 (Accelrys) software package. CDOCKER is a grid-based molecular docking algorithm that employs the CHARMm force field and samples the conformational space of flexible ligands through high-temperature molecular dynamics combined with simulated annealing. The crystal structures of target proteins were retrieved from the RCSB PDB database. PDB ID: 7WJO corresponds to *Ps*Chs1 from *P*. *sojae* in complex with nikkomycin Z, which was used to analyze the binding modes of designer analogs to the oomycete target; PDB ID: 7STO corresponds to *Ca*Chs2 from *C. albicans* bound to POL-D, which was used to analyze the binding modes of designer analogs to the fungal target. Prior to docking, the crystal structures were preprocessed using the “Prepare Protein” tool in Discovery Studio. The preprocessing steps included removal of water molecules, addition of hydrogen atoms, assignment of the CHARMm force field, and optimization of the protonation states of amino acid side chains. The three-dimensional structures of ligands, including natural polyoxin components and the designed POL analogs, were constructed using Chem3D and subsequently subjected to energy minimization using the “Prepare Ligands” tool in Discovery Studio to generate stable three-dimensional conformations. The binding sites were identified using the “From Receptor Cavities” function within the “Define and Edit Binding Site” tool. The docking region was defined as a sphere with a radius of 10 Å centered at the geometric center of the active pocket (for 7STO: X: 129.784825, Y: 96.689404, Z: 95.313123; for 7WJO: X: −21.010922, Y: −11.554328, Z: 16.685355). CDOCKER docking was performed with the following parameter settings: Top Hits set to 10; Pose Cluster Radius set to 0.1 Å; Random Conformations set to 10; Dynamics Target Temperature set to 1000 K; Simulated Annealing enabled; all other parameters were kept at their default values. Following docking, the generated poses were ranked according to the -CDOCKER_ENERGY and -CDOCKER_INTERACTION_ENERGY scoring functions. The optimal binding pose for each ligand was selected as the one with the lowest energy score that also exhibited the closest spatial orientation and binding mode similarity to the co-crystallized native ligand within the active pocket, and was subsequently used for detailed interaction analysis.

## CRediT authorship contribution statement

**Rong Gong:** Writing – original draft, Visualization, Validation, Methodology, Investigation, Funding acquisition, Data curation, Conceptualization. **Tianzhu Li:** Writing – original draft, Visualization, Validation, Methodology, Investigation, Data curation, Conceptualization. **Liwei Zhou:** Software, Investigation, Formal analysis, Data curation. **Yuanyuan Liu:** Investigation. **Tong Zhang:** Investigation, Data curation. **Yini Qin:** Validation, Investigation. **Xinyue Zeng:** Investigation. **Boyu Jin:** Investigation. **Jiahong Wang:** Writing – review & editing. **Zixin Deng:** Writing – review & editing. **Xuemin Song:** Writing – review & editing, Supervision, Project administration. **Wenqing Chen:** Writing – review & editing, Writing – original draft, Supervision, Project administration, Funding acquisition.

## Declaration of competing interest

The authors declare that they have no known competing financial interests or personal relationships that could have appeared to influence the work reported in this paper. The author Zixin Deng is the Founding Editor for Synthetic and Systems Biotechnology and was not involved in the editorial review or the decision to publish this article.
