# Supplementary material for: Directed biosynthesis of the designer peptidyl nucleoside antibiotics featuring a simplified azetidine-containing moiety
Source: Synth Syst Biotechnol. 2026 Sep 8;18:85–95. doi: 10.1016/j.synbio.2026.08.010 (PMC13571976; doi:10.1016/j.synbio.2026.08.010)
Supplement: Supplementary file 1 [file mmc1.pdf]

Supporting Information

Directed biosynthesis of the designer peptidyl nucleoside antibiotics featuring a simplified azetidine-containing moiety

Rong Gong[1#], Tianzhu Li[1#], Liwei Zhou[1], Yuanyuan Liu[1], Tong Zhang[1], Yini Qin[1], Xinyue Zeng[1], Boyu Jin[1], Jiahong Wang[2], Zixin Deng[1,3], Xuemin Song[1]*, Wenqing Chen[1,3]*

[1]Department of Anesthesiology, Zhongnan Hospital of Wuhan University, School of Pharmaceutical Sciences, Wuhan University, Wuhan 430071, China.

[2]International Innovation Center for Forest Chemicals and Material, Nanjing Forestry University, Nanjing 210037, China.

[3]Key Laboratory of Combinatorial Biosynthesis and Drug Discovery, Ministry of Education, Wuhan University, Wuhan 430071, China.

[#]These authors contributed equally: Rong Gong, Tianzhu Li.

*E-mail: wqchen@whu.edu.cn, or xueminsong@whu.edu.cn

Table of contents

1. Supplementary Figures **5**

Figure S1. Overall biosynthetic pathway of polyoxin (POL). 5

Figure S2. Construction and metabolites analysis of the *S. aureochromogenes* CXR3 strain. 6

Figure S3. LC-HRMS analysis of the target metabolites produced by the strain CXR3 fed with related POIA analogs. 8

Figure S4. LC-HRMS/MS analysis of the target metabolites separately produced by the strain CXR3 fed with related POIA analogs. 9

Figure S5. Construction and metabolites analysis of the strain CXR6. 11

Figure S6. Bioassay and HPLC analysis of the target metabolites separately produced by the strain CXR6 fed with AZE, L-A2CA, D-A2CA or 3ACA. 12

Figure S7. LC-HRMS analysis of the target metabolites individually produced by the strain CXR6 fed with POIA analogs.. 13

Figure S8. LC-HRMS/MS analysis of the target metabolites correspondingly produced by the strain CXR6 fed with of L-A2CA, D-A2CA, or 3ACA. 14

Figure S9. The proposed biosynthetic pathway of azetidomonamides. 15

Figure S10. LC-HRMS analysis of the target metabolites independently produced by the recombinants CXR3::pIB139/*azeJ* and CXR6::pIB139/*azeJ*. 16

Figure S11. ^{1}H NMR spectrum of POL-A_X (D_2O, 400 MHz). 18

Figure S12. ^{13}C NMR spectrum of POL-A_X (D_2O, 100 MHz). 18

Figure S13. ^{1}H-^{1}H COSY spectrum of POL-A_X (D_2O, 400 MHz). 19

Figure S14. HSQC spectrum of POL-A_X (D_2O, 400 MHz). 19

Figure S15. HMBC spectrum of POL-A_X (D_2O, 400 MHz). 20

Figure S16. ^{1}H NMR spectrum of POL-H_X (D_2O, 400 MHz). 20

Figure S17. ^{13}C NMR spectrum of POL-H_X (D_2O, 100 MHz). 21

Figure S18. ^{1}H-^{1}H COSY spectrum of POL-H_X (D_2O, 400 MHz). 21

Figure S19. HSQC spectrum of POL-H_X (D_2O, 400 MHz). 22

Figure S20. HMBC spectrum of POL-H_X (D_2O, 400 MHz). 22

Figure S21. ^{1}H NMR spectrum of POL-K_X (D_2O, 600 MHz). 23

Figure S22. ^{13}C NMR spectrum of POL-K_X (D_2O, 150 MHz). 23

Figure S23. ^{1}H-^{1}H COSY spectrum of POL-K_X (D_2O, 600 MHz). 24

Figure S24. HSQC spectrum of POL-K_X (D_2O, 600 MHz)................................24
Figure S25. HMBC spectrum of POL-K_X (D_2O, 600 MHz)...............................25
Figure S26. HPLC analysis of the target metabolites produced by the recombinant CXR6::pIB139/*azeJ*&*vioG*...26
Figure S27. LC-HRMS analysis of the target metabolites produced by the recombinant CXR6::pIB139/*azeJ*&*vioG*...27
Figure S28. Construction and metabolites analysis of the strain CPO1. 28
Figure S29. Construction and metabolites analysis of the strain CPO2. 30
Figure S30. LC-HRMS analysis of the target metabolites individually produced by the recombinants CPO1::pIB139/*azeJ* and CPO2::pIB139/*azeJ*...................31
Figure S31. ^{1}H NMR spectrum of POL-I_X (D_2O, 600 MHz)..............................33
Figure S32. ^{13}C NMR spectrum of POL-I_X (D_2O, 150 MHz).............................33
Figure S33. ^{1}H-^{1}H COSY NMR spectrum of POL-I_X (D_2O, 600 MHz).............34
Figure S34. HSQC NMR spectrum of POL-I_X (D_2O, 600 MHz). 34
Figure S35. HMBC NMR spectrum of POL-I_X (D_2O, 600 MHz). 35
Figure S36. ^{1}H NMR spectrum of thymine POL-I_X (D_2O, 600 MHz)................35
Figure S37. ^{13}C NMR spectrum of thymine POL-I_X (D_2O, 150 MHz). 36
Figure S38.^{1}H-^{1}H COSY NMR spectrum of thymine POL-I_X (D_2O, 600 MHz).36
Figure S39. HSQC NMR spectrum of thymine POL-I_X (D_2O, 600 MHz). 37
Figure S40. HMBC NMR spectrum of thymine POL-I_X (D_2O, 600 MHz). 37
Figure S41. ^{1}H NMR spectrum of uracil POL-I_X (D_2O, 600 MHz). 38
Figure S42. ^{13}C NMR spectrum of uracil POL-I_X (D_2O, 150 MHz). 38
Figure S43. ^{1}H-^{1}H COSY spectrum of uracil POL-I_X (D_2O, 600 MHz).............39
Figure S44. HSQC spectrum of uracil POL-I_X (D_2O, 600 MHz). 39
Figure S45. HMBC spectrum of uracil POL-I_X (D_2O, 600 MHz). 40
Figure S46. Molecular docking of related POL analogs with the chitin synthase *Ca*Chs2...41
Figure S47. Molecular docking of related POL analogs with the chitin synthase *Ps*Chs1. 42
2. Supplementary Tables 43
Table S1. Strains and plasmids used in this study...43
Table S2. Primers used in this study. 45
Table S3. Validation of the mutant strains by sequencing analysis. 46
Table S4. NMR data of POL-A_X (D_2O, 400 MHz)...47

Table S5. NMR data of POL-H_X (D_2O, 400 MHz). 48
Table S6. NMR data of POL-K_X (D_2O, 600 MHz). 49
Table S7. NMR data of POL-I_X (D_2O, 600 MHz). 50
Table S8. NMR data of thymine POL-I_X (D_2O, 600 MHz). 51
Table S9. NMR data of uracil POL-I_X (D_2O, 600 MHz). 52
Table S10. Codon-optimized sequence of *azeJ*. 53
Table S11. Quantitative docking scores. 54
3. Supplementary References..55

1. Supplementary Figures

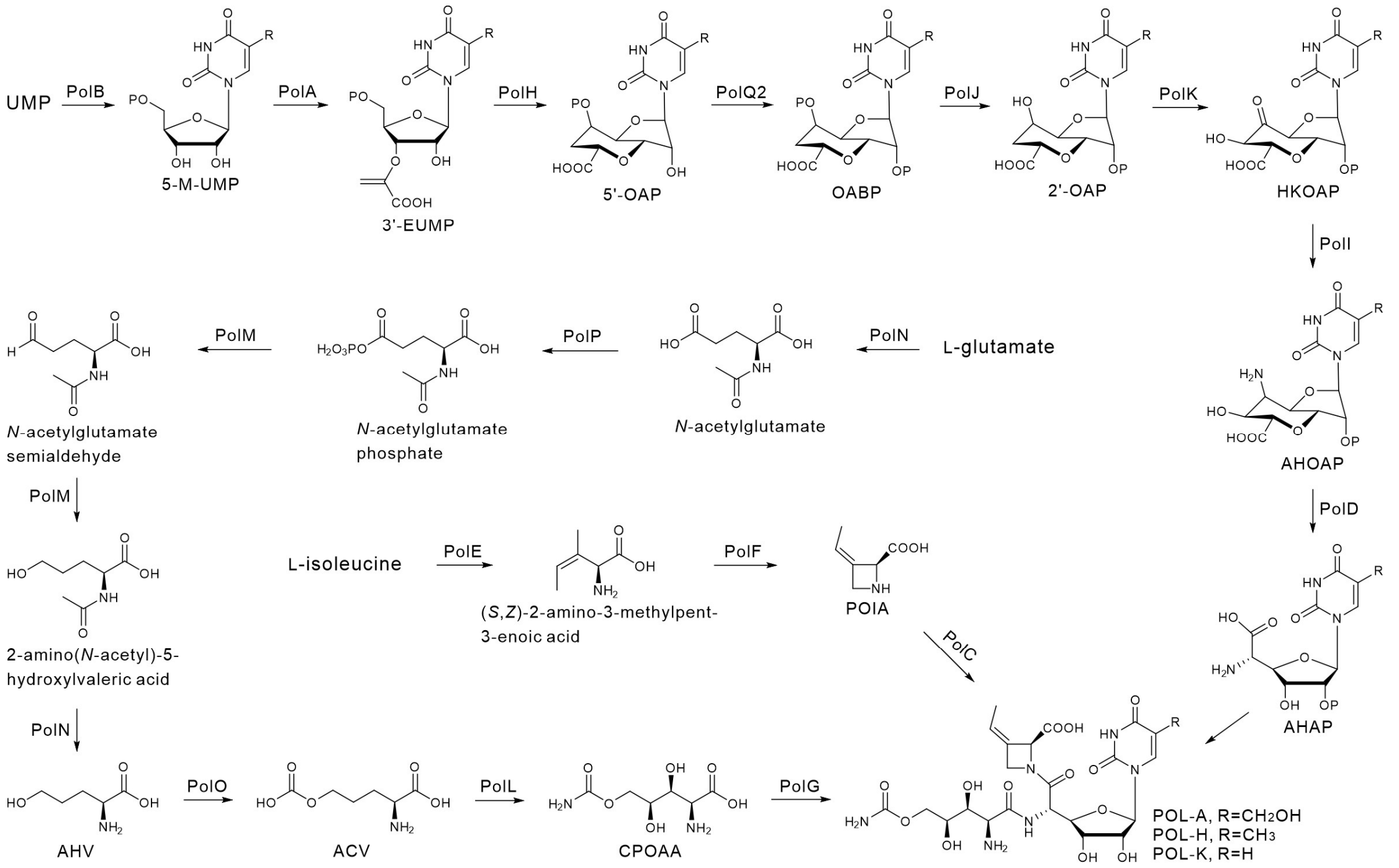

Figure S1. Overall biosynthetic pathway of polyoxin (POL). As indicated in the overall biosynthetic pathway of POL[1], the genes *polABDHIJKQ2* were confirmed for the nucleoside skeleton formation[2-5], the *polLMNOP* were defined for CPOAA biosynthesis[6], the *polEF* were responsible for POIA biosynthesis[7], and *polG* was characterized for the assembly of AHAP and CPOAA[8]. Notably, the gene *polC* was proposed to be responsible for the assembly of AHAP and POIA. UMP, uridine 5′-monophosphate; 5-M UMP, 5-methyl UMP; 3′-EUMP, 3′-enolpyruvyl UMP; 5′-OAP, octosyl acid 5′-phosphate; OABP, octosyl acid 2′,5′-bisphosphate; 2′-OAP, octosyl acid 2′-phosphate; HKOAP, 6′-hydroxy-5′-keto-octosyl acid 2′-phosphate; AHOAP, (4′*S*, 5′*S*, 6′*S*, 7′*R*)-5′-amino-6′-hydroxyoctosyl acid 2′-phosphate; AHAP, amino-hexuronic acid 2′-phosphate; AHV, α-amino-δ-hydroxyvaleric acid; ACV, α-amino-δ-carbamoylhydroxyvaleric acid; CPOAA, carbamoylpolyoxamic acid; POIA, polyoximic acid; POL-A, polyoxin A; POL-H, polyoxin H; POL-K, polyoxin K.

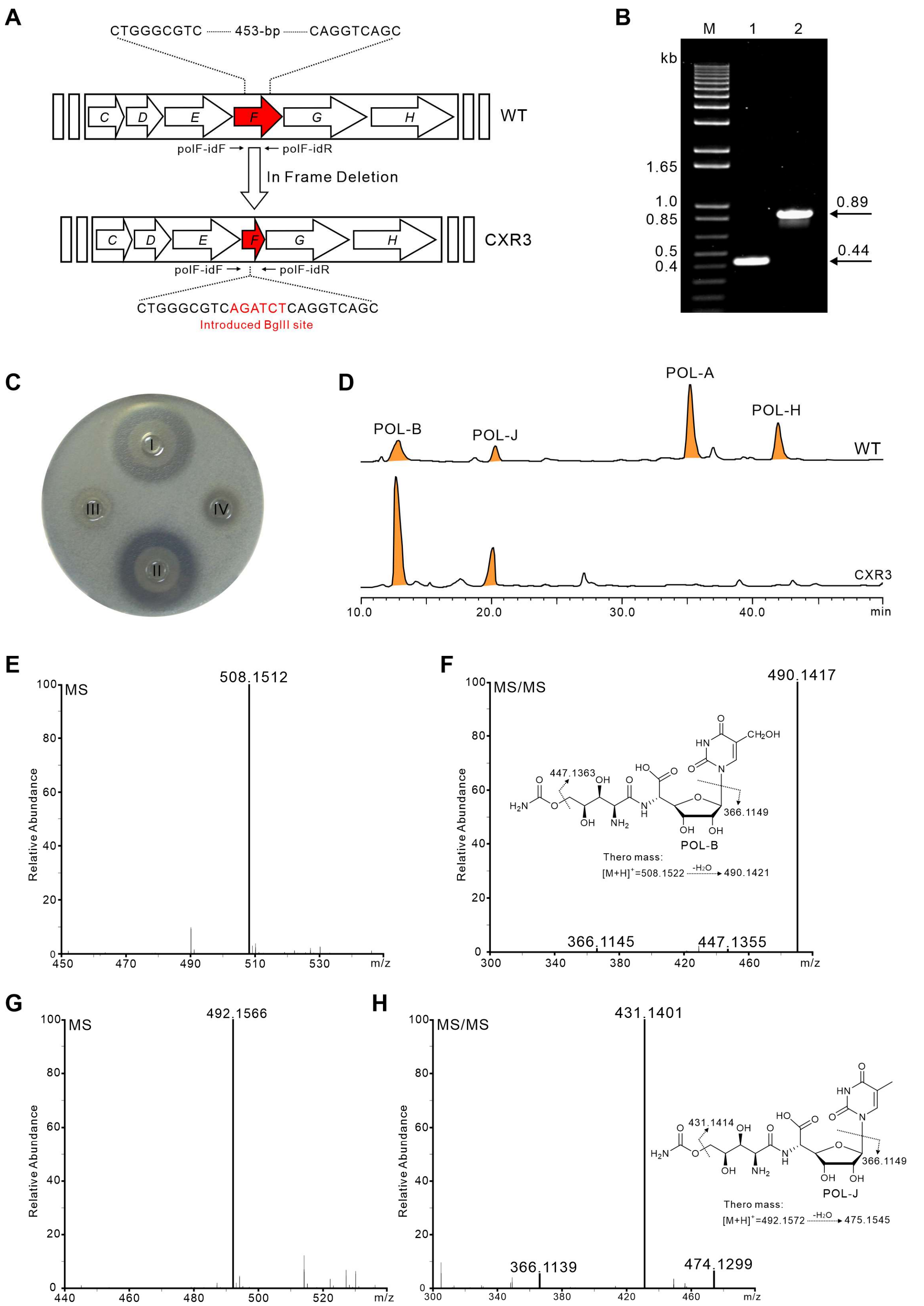

Figure S2. Construction and metabolites analysis of the *S. aureochromogenes* CXR3 strain. (A) Schematic illustration for the construction of the CXR3 strain. (B) PCR identification of the CXR3 strain. M, 1 kb ladder; 1, PCR product was amplified using genomic DNA of *S. aureochromogenes* as template. The expected size of the PCR

product was 0.89 kb; 2, PCR product was amplified using genomic DNA of the CXR3 strain as template. The expected size of the PCR product was 0.44 kb. (C) Bioassay analysis of the metabolites produced by related strains using *Trichosporon cutaneum* as indicator strain. I and II represent the target metabolites produced by the strain *S. aureochromogenes*; III and IV indicate the target metabolites produced by the strain CXR3. (D) HPLC analysis (wavelength λ = 260 nm) of the metabolites produced by related strains. WT, metabolites of the *S. aureochromogenes*; CXR3, metabolites of the CXR3 strain. (E) LC-HRMS analysis of the target metabolite POL-B produced by the CXR3 strain. (F) LC-HRMS/MS analysis of the target metabolite POL-B produced by the CXR3 strain. The theoretical fragmentation pattern of POL-B is included as a control. (G) LC-HRMS analysis of the target metabolite POL-J produced by the CXR3 strain. (H) LC-HRMS/MS analysis of the target metabolite POL-J produced by the CXR3 strain. The theoretical fragmentation pattern of POL-J is included as a control.

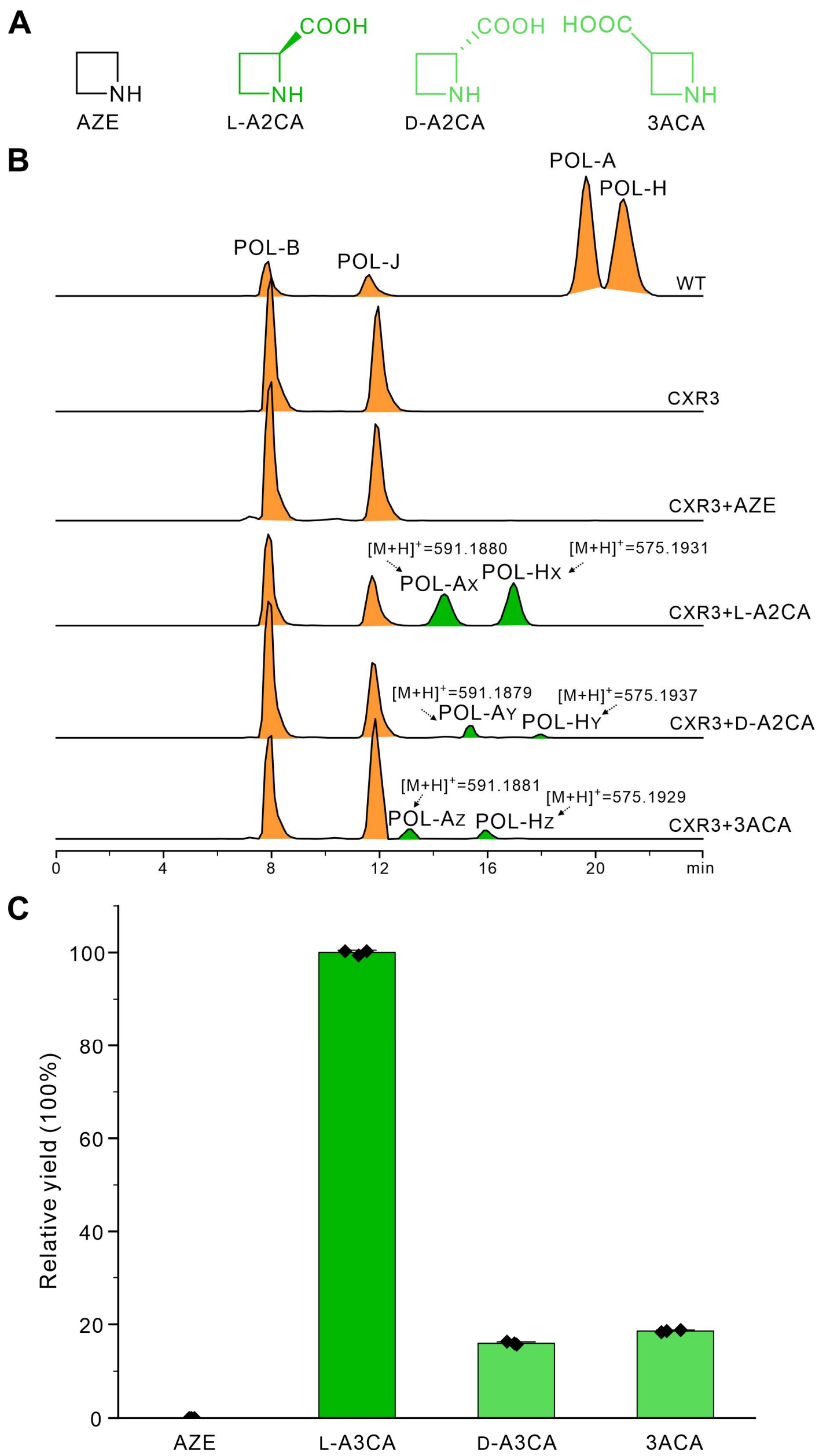

Figure S3. LC-HRMS analysis of the target metabolites produced by the strain CXR3 fed with related POIA analogs. (A) Chemical structures of related POIA analogs. AZE, azetidine; L-A2CA, L-azetidine-2-carboxylic acid; D-A2CA, D-azetidine-2-carboxylic acid; 3ACA, 3-azetidinecarboxylic acid. (B) LC-HRMS analysis of the target metabolites individually produced by the strain CXR3 fed with AZE, L-A2CA, D-A2CA, or 3ACA. (C) Relative yield of metabolites derived from different POIA analogs determined by LC-HRMS peak area. L-A2CA exhibited highly efficient incorporation, whereas D-A2CA and 3ACA were incorporated only at low levels, and AZE showed no detectable product.

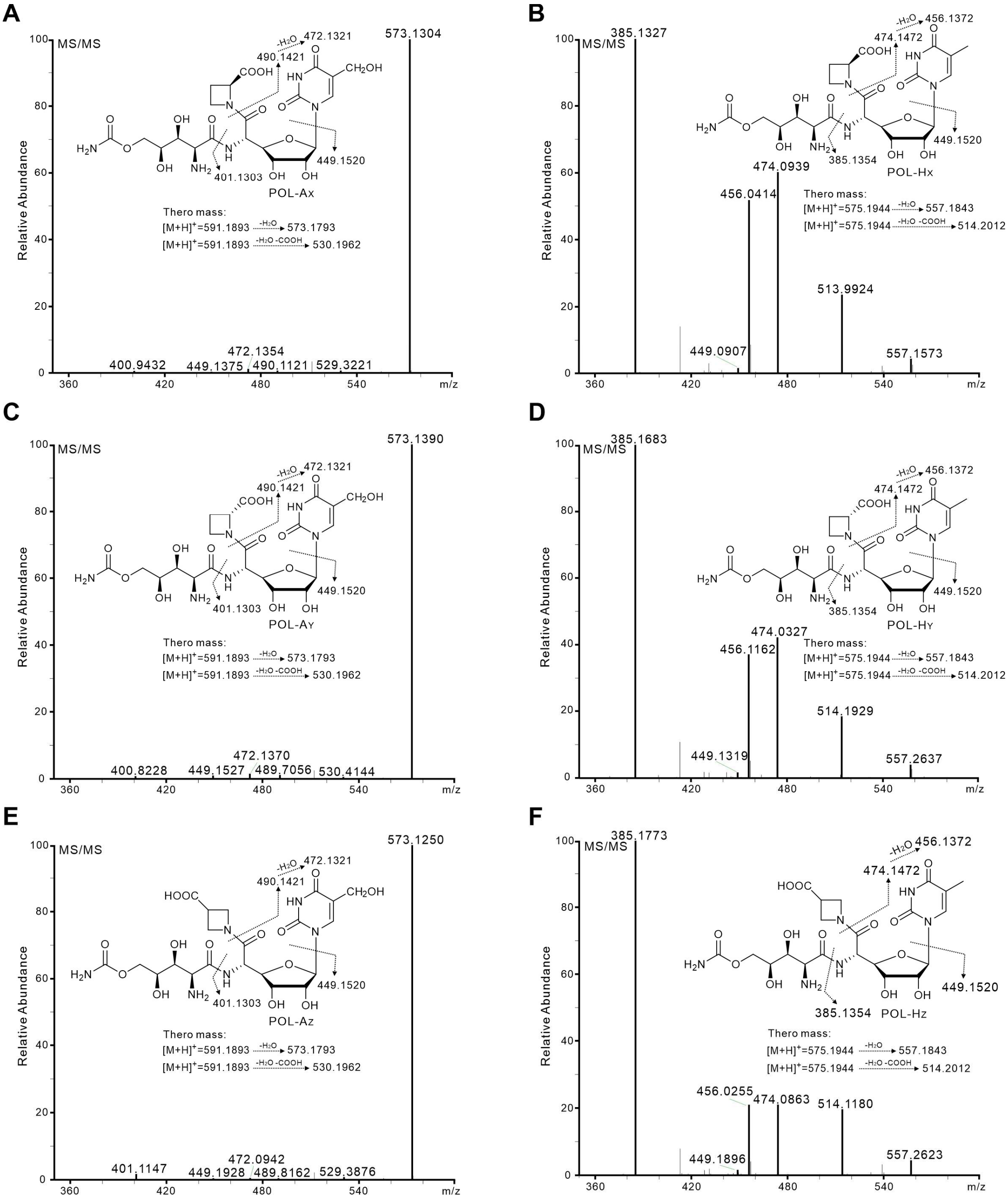

Figure S4. LC-HRMS/MS analysis of the target metabolites separately produced by the strain CXR3 fed with related POIA analogs. (A) LC-HRMS/MS analysis of the target metabolite POL-A$_X$ produced by the strain CXR3 fed with L-A2CA. The theoretical fragmentation pattern of POL-Ax is included as a control. (B) LC-HRMS/MS analysis of the target metabolite POL-H$_X$ produced by the strain CXR3 fed with L-A2CA. The theoretical fragmentation pattern of POL-Hx is included as a control. (C) LC-HRMS/MS analysis of the target metabolite POL-A$_Y$ produced by the strain CXR3 fed with D-A2CA. The theoretical fragmentation pattern of POL-A$_Y$ is included

as a control. (D) LC-HRMS/MS analysis of the target metabolite POL-H_Y produced by the strain CXR3 fed with D-A2CA. The theoretical fragmentation pattern of POL-H_Y is included as a control. (E) LC-HRMS/MS analysis of the target metabolite POL-A_Z produced by the strain CXR3 fed with 3ACA. The theoretical fragmentation pattern of POL-A_Z is included as a control. (F) LC-HRMS/MS analysis of the target metabolite POL-H_Z produced by the strain CXR3 fed with 3ACA. The theoretical fragmentation pattern of POL-H_Z is included as a control.

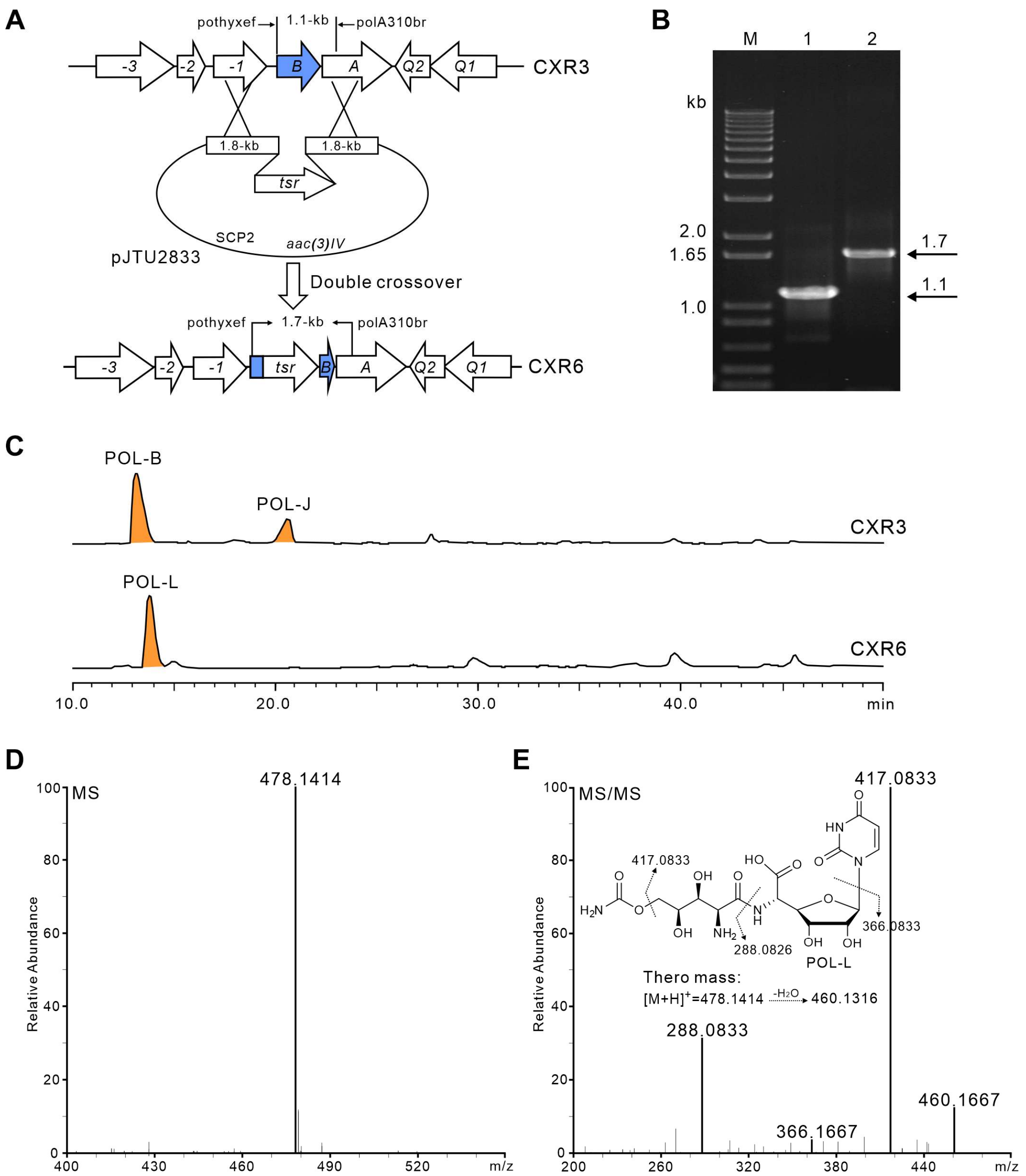

Figure S5. Construction and metabolites analysis of the CXR6 strain. (A) Schematic illustration for the construction of the CXR6 strain. (B) PCR identification of CXR6. M, 1 kb ladder; 1, PCR product was amplified using genomic DNA of CXR3 as template. The expected size of the PCR product was 1.1 kb; 2, PCR product was amplified using genomic DNA of CXR6 as template. The expected size of the PCR product was 1.7 kb. (C) HPLC analysis of the metabolites produced by related strains. CXR3, metabolites of the CXR3 strain; CXR6, metabolites of the CXR6 strain. (D) LC-HRMS analysis of the target metabolite POL-L produced by the CXR6 strain. (E)

LC-HRMS/MS analysis of the target metabolite POL-L produced by the CXR6 strain. The theoretical fragmentation pattern of POL-L is included as a control.

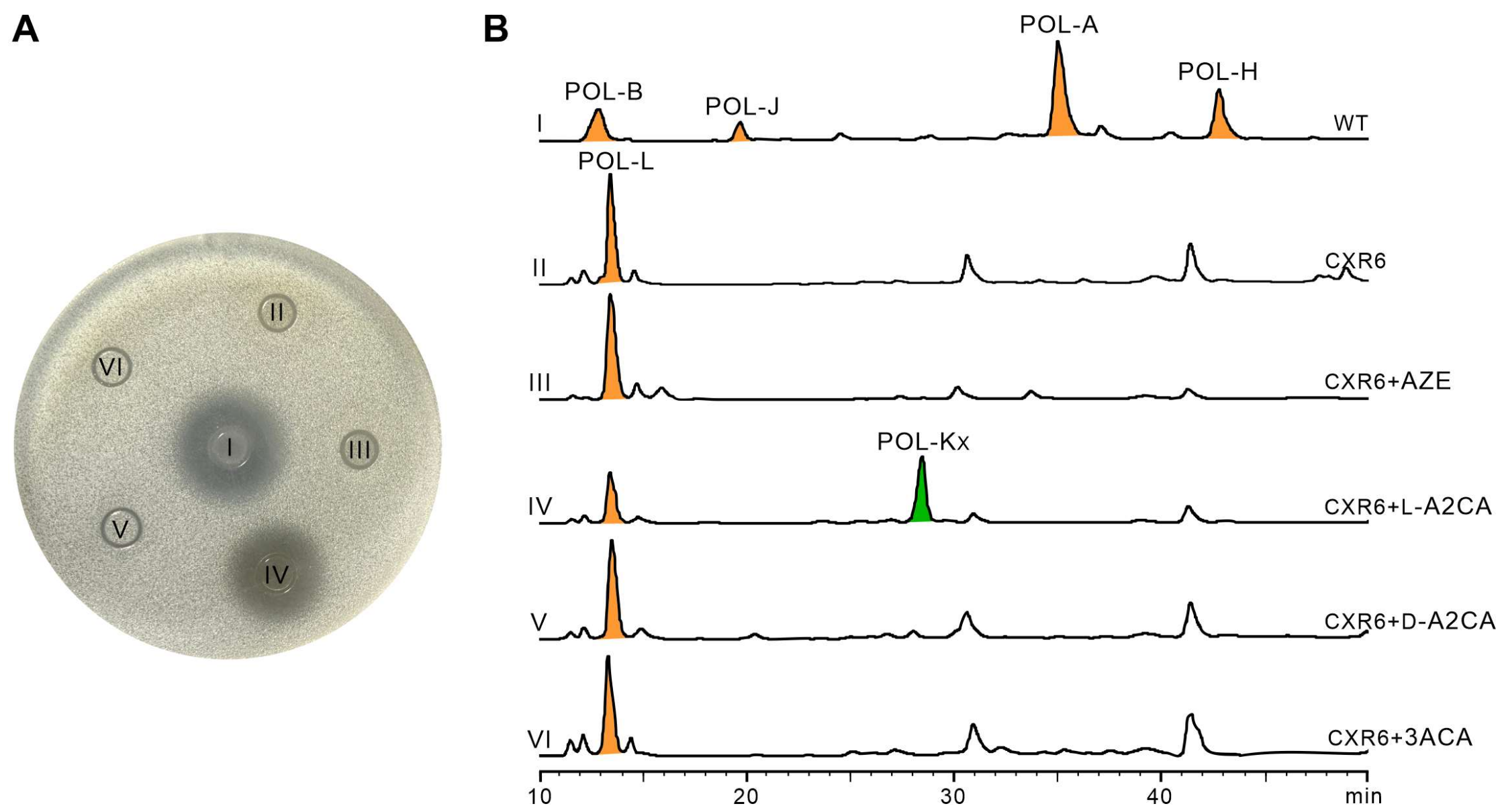

Figure S6. Bioassay and HPLC analysis of the target metabolites separately produced by the strain CXR6 fed with AZE, L-A2CA, D-A2CA or 3ACA. (A) Bioassay analysis of the metabolites separately produced by the strain CXR6 fed with AZE, L-A2CA, D-A2CA or 3ACA. *T. cutaneum* was used as indicator strain. I, metabolites produced by the strain *S. aureochromogenes*; II, metabolites produced by the strain CXR6; III, metabolites produced by the strain CXR6 fed with AZE; IV, metabolites produced by the strain CXR6 fed with L-A2CA; V, metabolites produced by the strain CXR6 fed with D-A2CA; VI, metabolites produced by the strain CXR6 fed with 3ACA. (B) HPLC analysis of the target metabolites separately produced by the strain CXR6 fed with AZE, L-A2CA, D-A2CA and 3ACA. The denotations of them correspond to those shown in panel A.

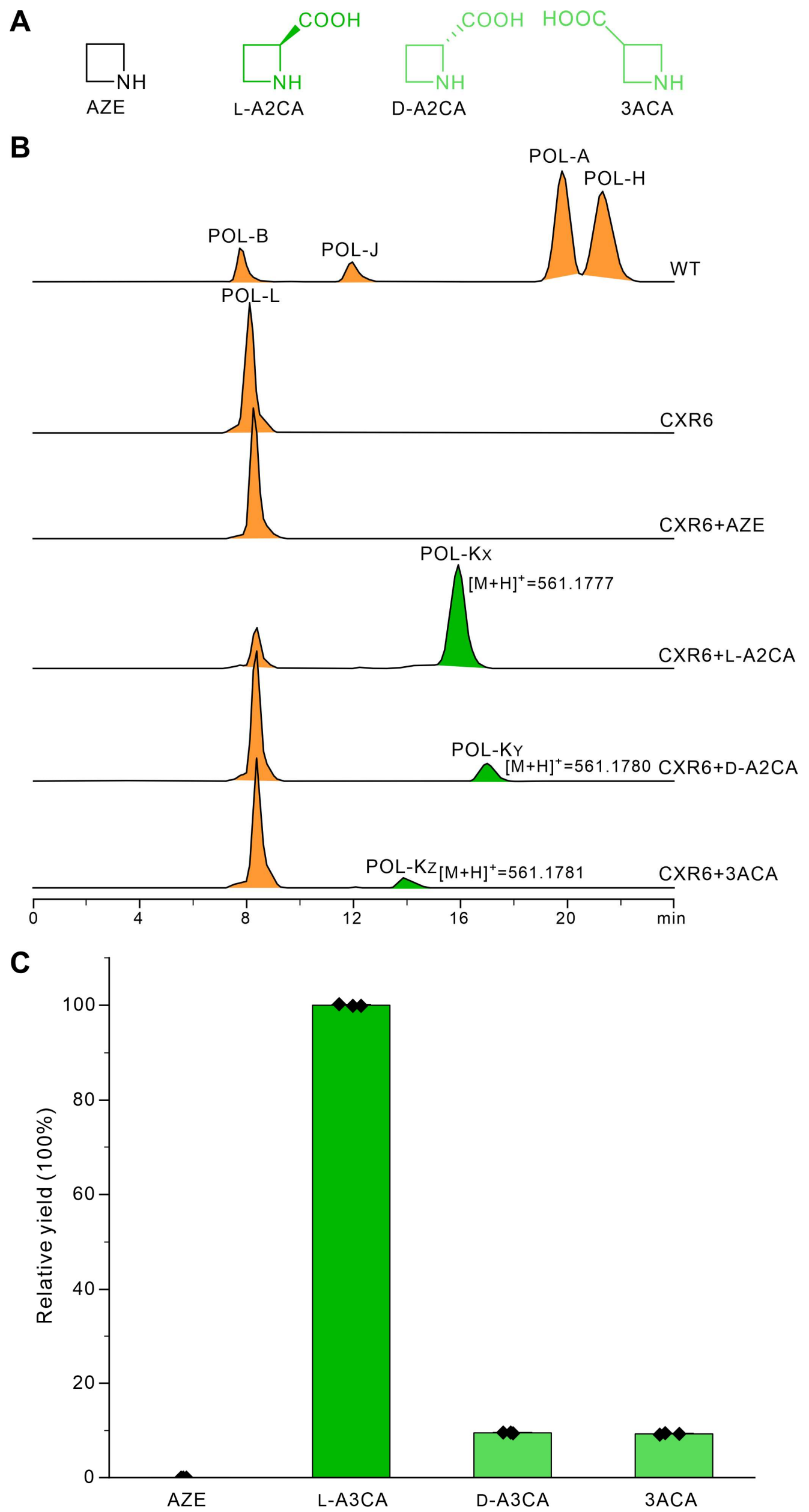

Figure S7. LC-HRMS analysis of the target metabolites individually produced by the strain CXR6 fed with POIA analogs. (A) Chemical structures of related POIA analogs. AZE, azetidine; L-A2CA, L-azetidine-2-carboxylic acid; D-A2CA, D-azetidine-2-carboxylic acid; 3ACA, 3-azetidinecarboxylic acid. (B) LC-HRMS analysis of the target metabolites individually produced by the strain CXR6 fed with AZE, L-A2CA, D-A2CA, or 3ACA. (C) Relative yield of metabolites derived from different POIA analogs determined by LC-HRMS peak area. L-A2CA exhibited highly efficient incorporation, whereas D-A2CA and 3ACA were incorporated only at low levels, and AZE showed no detectable product.

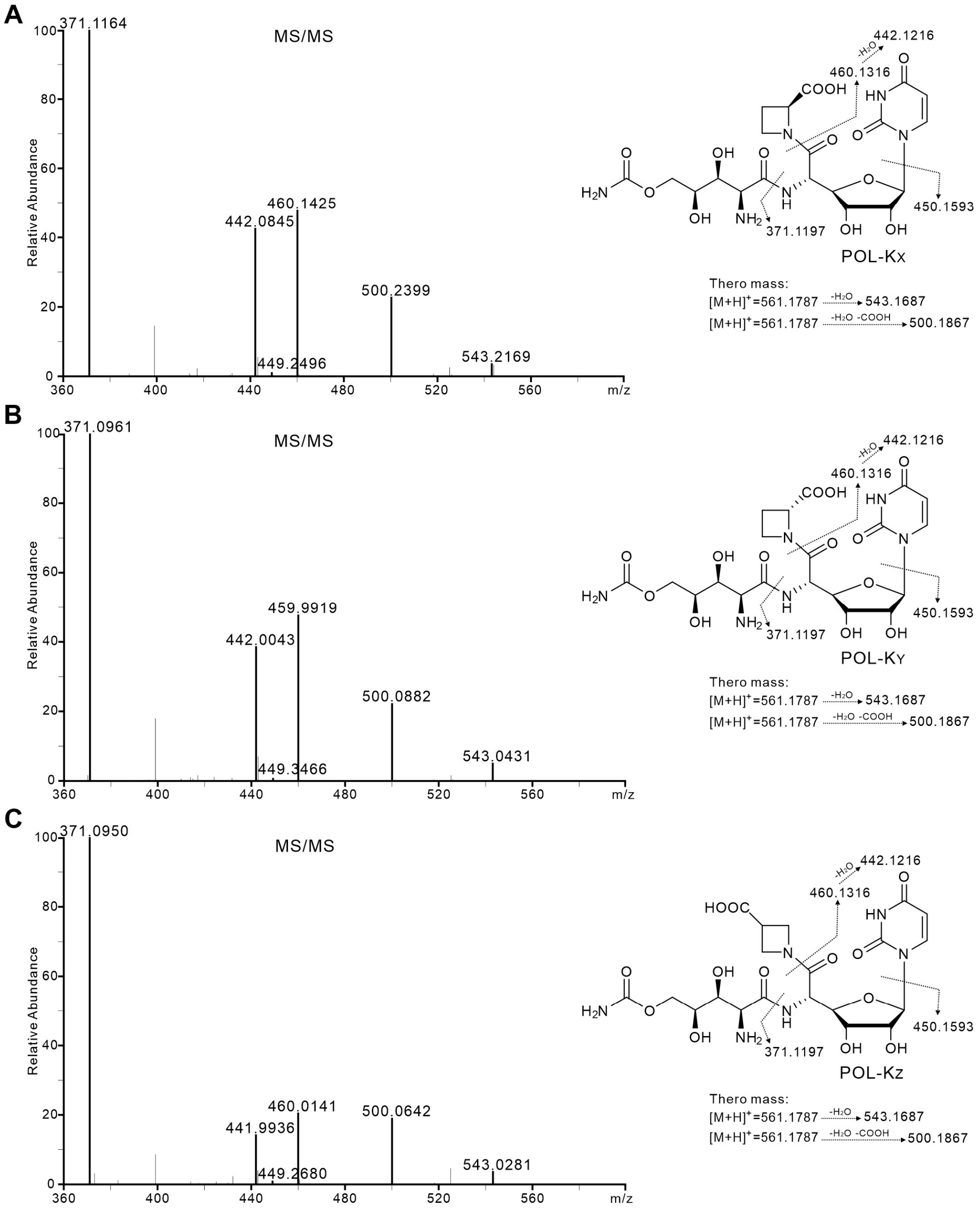

Figure S8. LC-HRMS/MS analysis of the target metabolites correspondingly produced by the strain CXR6 fed with of L-A2CA, D-A2CA, or 3ACA. (A) LC-HRMS/MS analysis of the target metabolite POL-KX produced by the strain CXR6 fed with L-A2CA. The theoretical fragmentation pattern of POL-KX is included as a control. (B) LC-HRMS/MS analysis of the target metabolite POL-KY produced by the strain CXR6 fed with D-A2CA. The theoretical fragmentation pattern of POL-KY is included as a control. (C) LC-HRMS/MS analysis of POL-KZ produced by the strain CXR6 fed with 3ACA**.** The theoretical fragmentation pattern of POL-KZ is included as a control.

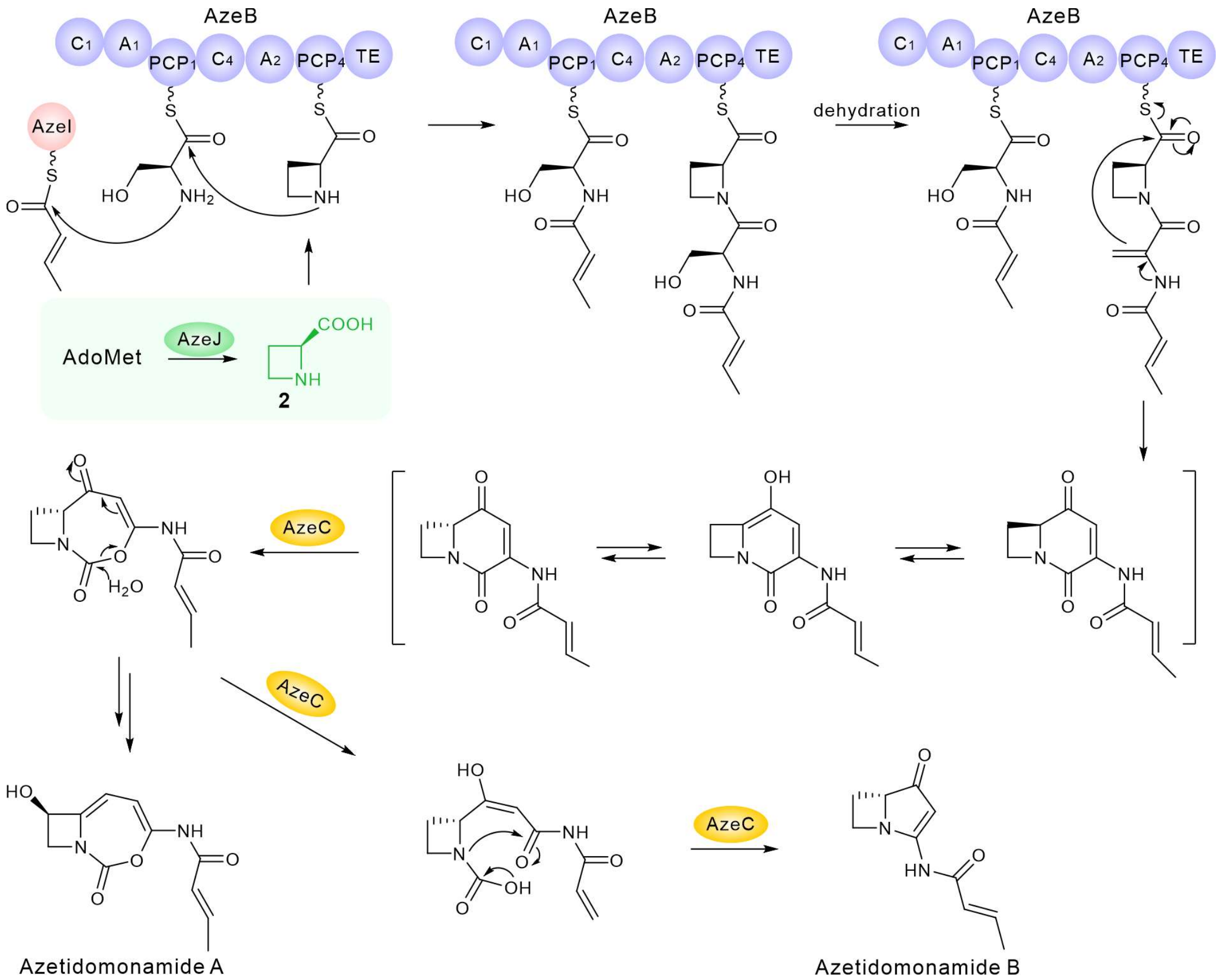

Figure S9. The proposed biosynthetic pathway of azetidomonamides. The AzeJ protein (Accession no. NP_252025, from *Pseudomonas aeruginosa* PAO1) is responsible for the biosynthesis of L-azetidine-2-carboxylic acid (L-A2CA)[9, 10]. AdoMet, *S*-adenosylmethionine.

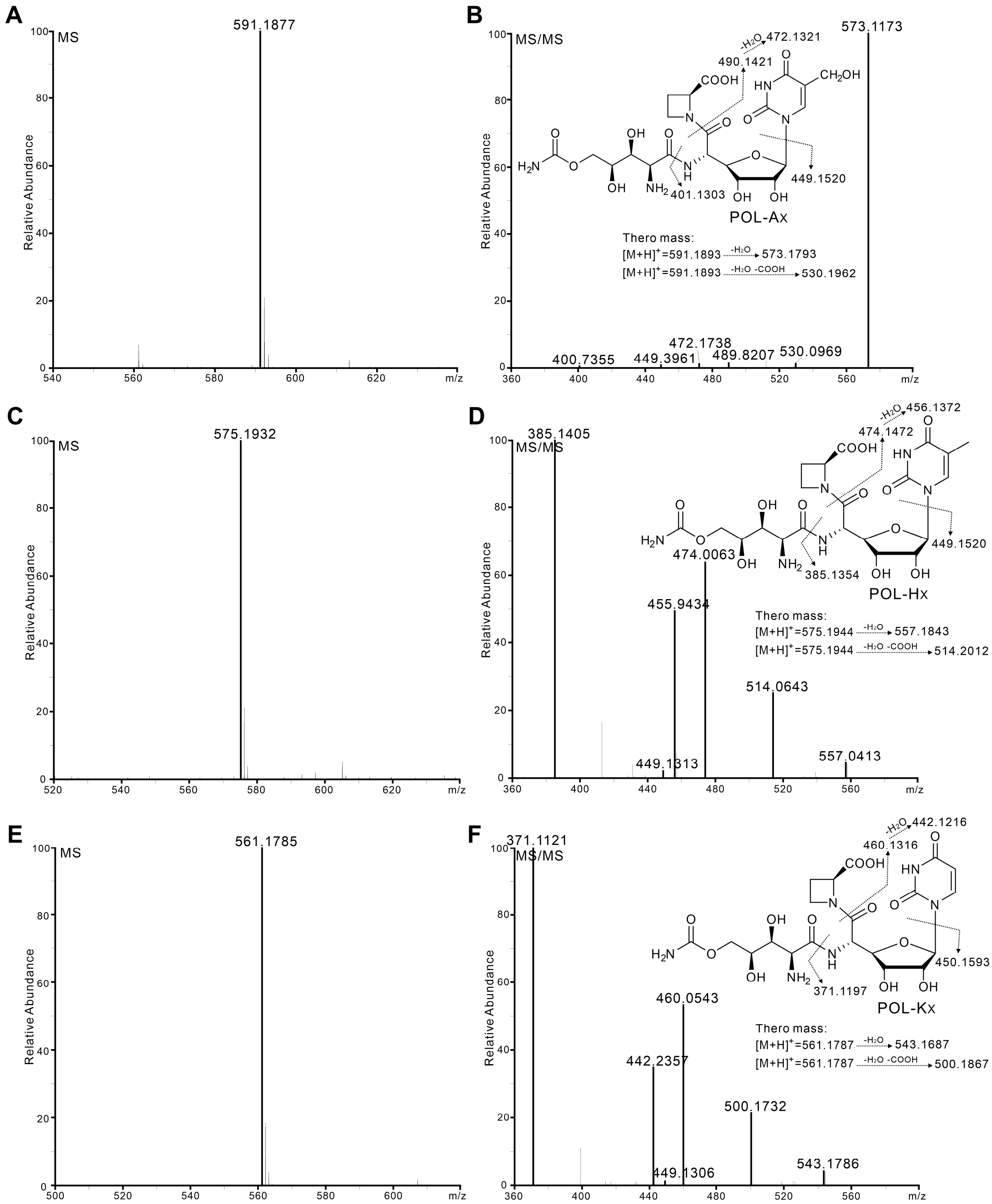

Figure S10. LC-HRMS analysis of the target metabolites independently produced by the recombinants CXR3::pIB139/*azeJ* and CXR6::pIB139/*azeJ*. (A) LC-HRMS analysis of the target metabolite POL-A_X produced by the recombinant CXR3::pIB139/*azeJ*. (B) LC-HRMS/MS analysis of the target metabolite POL-A_X produced by the recombinant CXR3::pIB139/*azeJ*. The theoretical fragmentation pattern of POL-A_X is included as a control. (C) LC-HRMS analysis of the target metabolite POL-H_X produced by the recombinant CXR3::pIB139/*azeJ*. (D) LC-HRMS/MS analysis of the target metabolite POL-H_X produced by the recombinant

CXR3::pIB139/*azeJ*. The theoretical fragmentation pattern of POL-H_X is included as a control. (E) LC-HRMS analysis of POL-K_X produced by the recombinant CXR6::pIB139/*azeJ*. (F) LC-HRMS/MS analysis of POL-K_X produced by the recombinant CXR6::pIB139/*azeJ*. The theoretical fragmentation pattern of POL-K_X is included as a control.

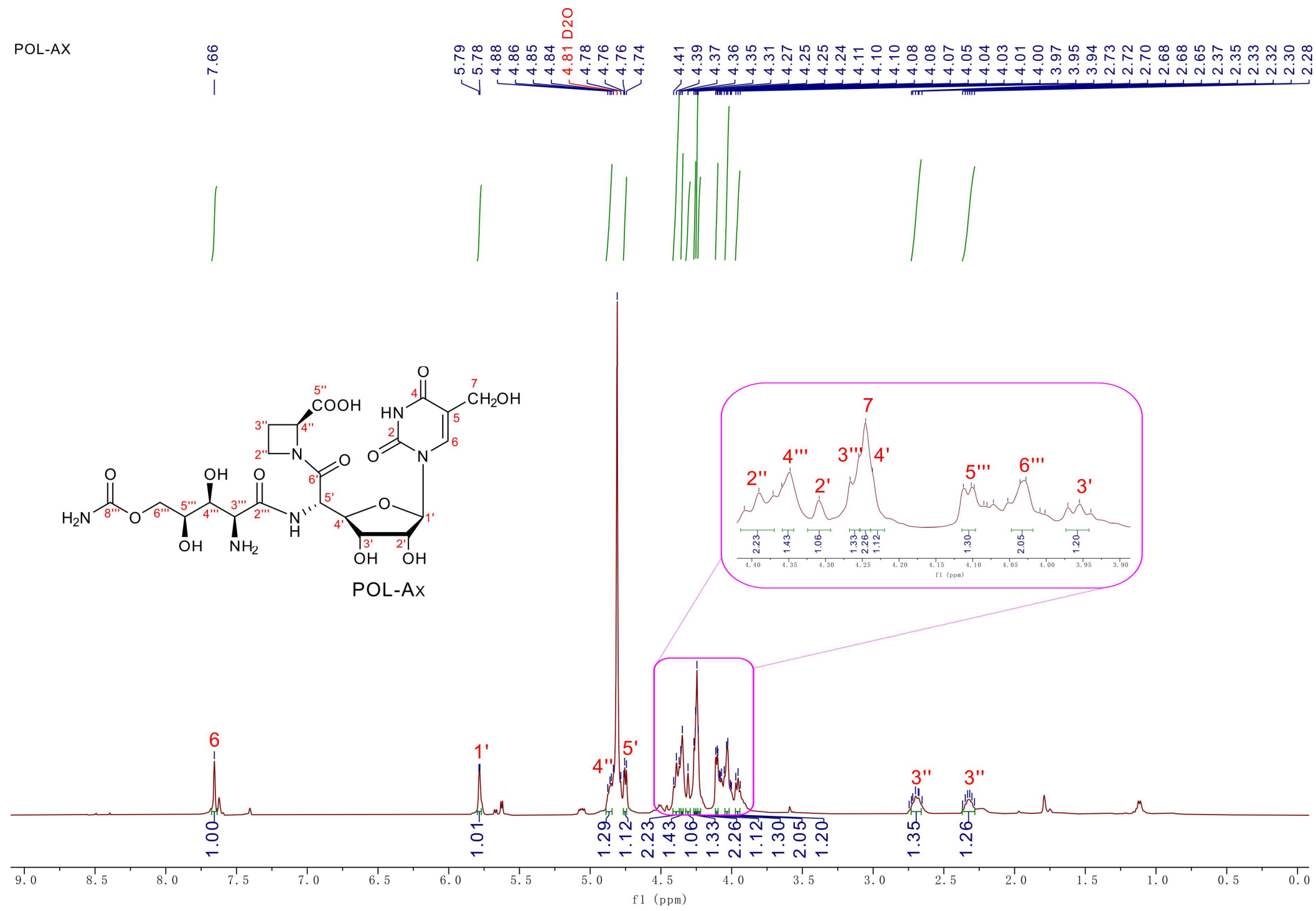

Figure S11. ^{1}**H NMR spectrum of POL-Ax (D**$_2$**O, 400 MHz).**

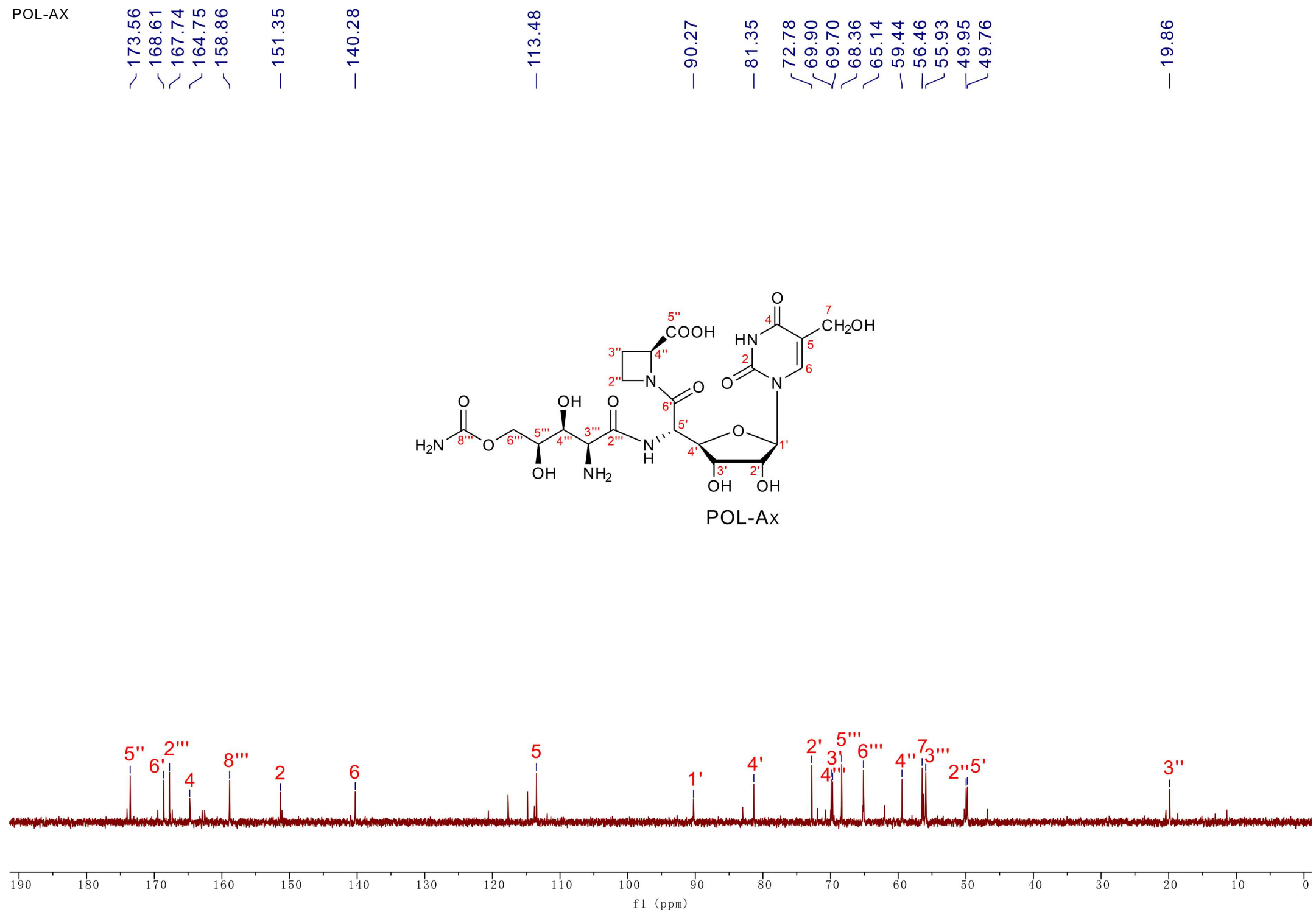

Figure S12. ^{13}**C NMR spectrum of POL-Ax (D**$_2$**O, 100 MHz).**

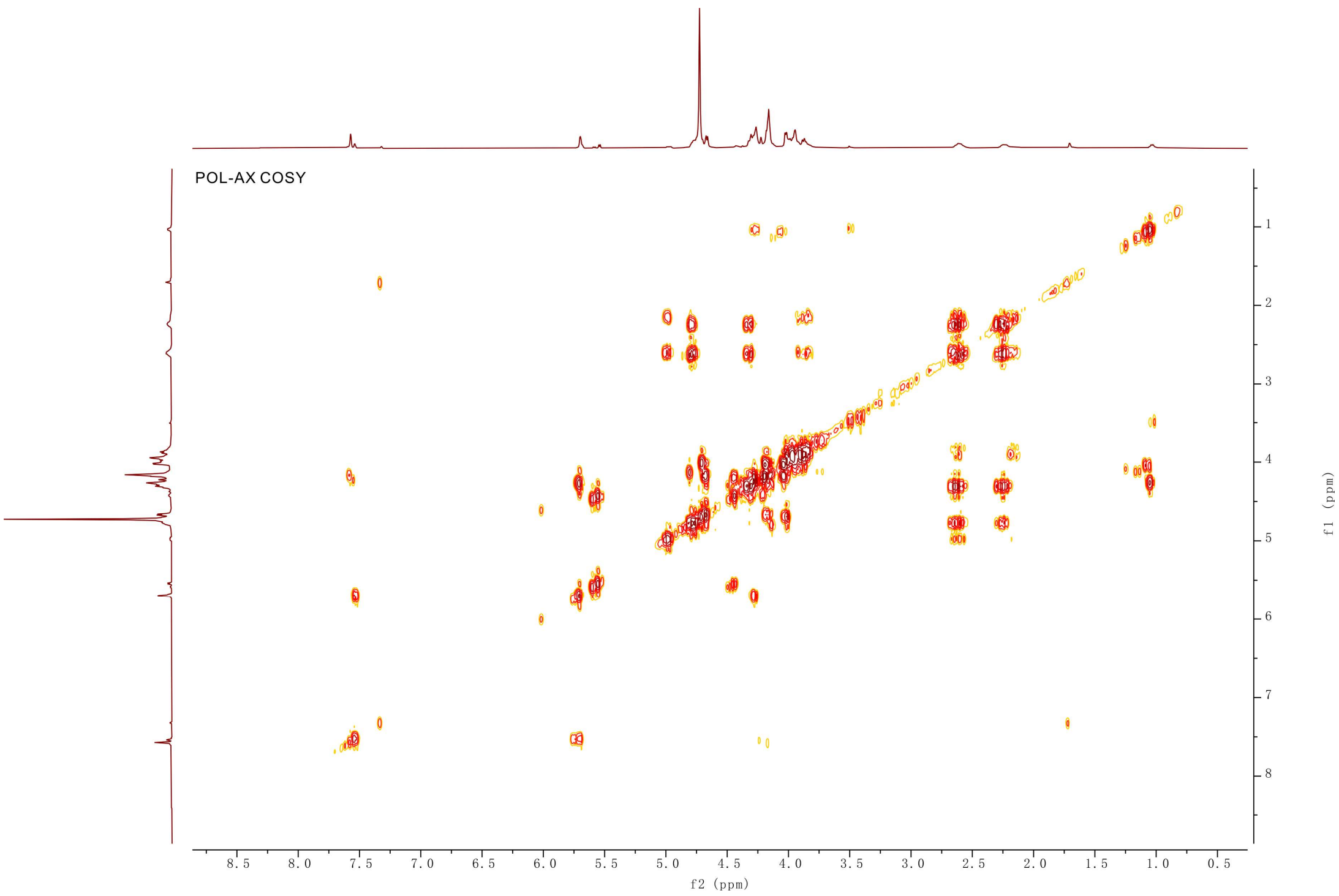

Figure S13. ^{1}H-^{1}H **COSY spectrum of POL-Ax (D_2O, 400 MHz).**

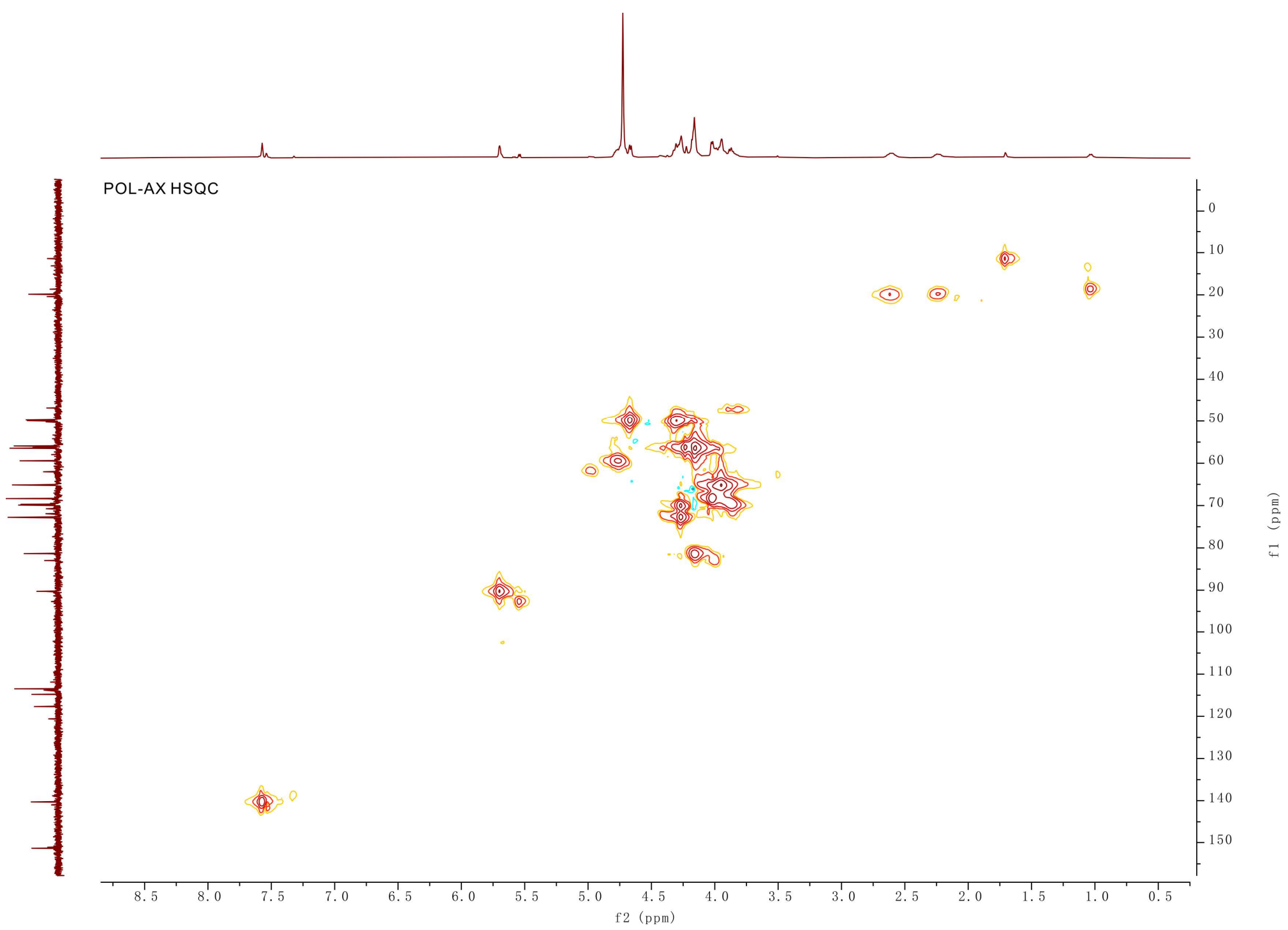

Figure S14. HSQC spectrum of POL-Ax (D_2O, 400 MHz).

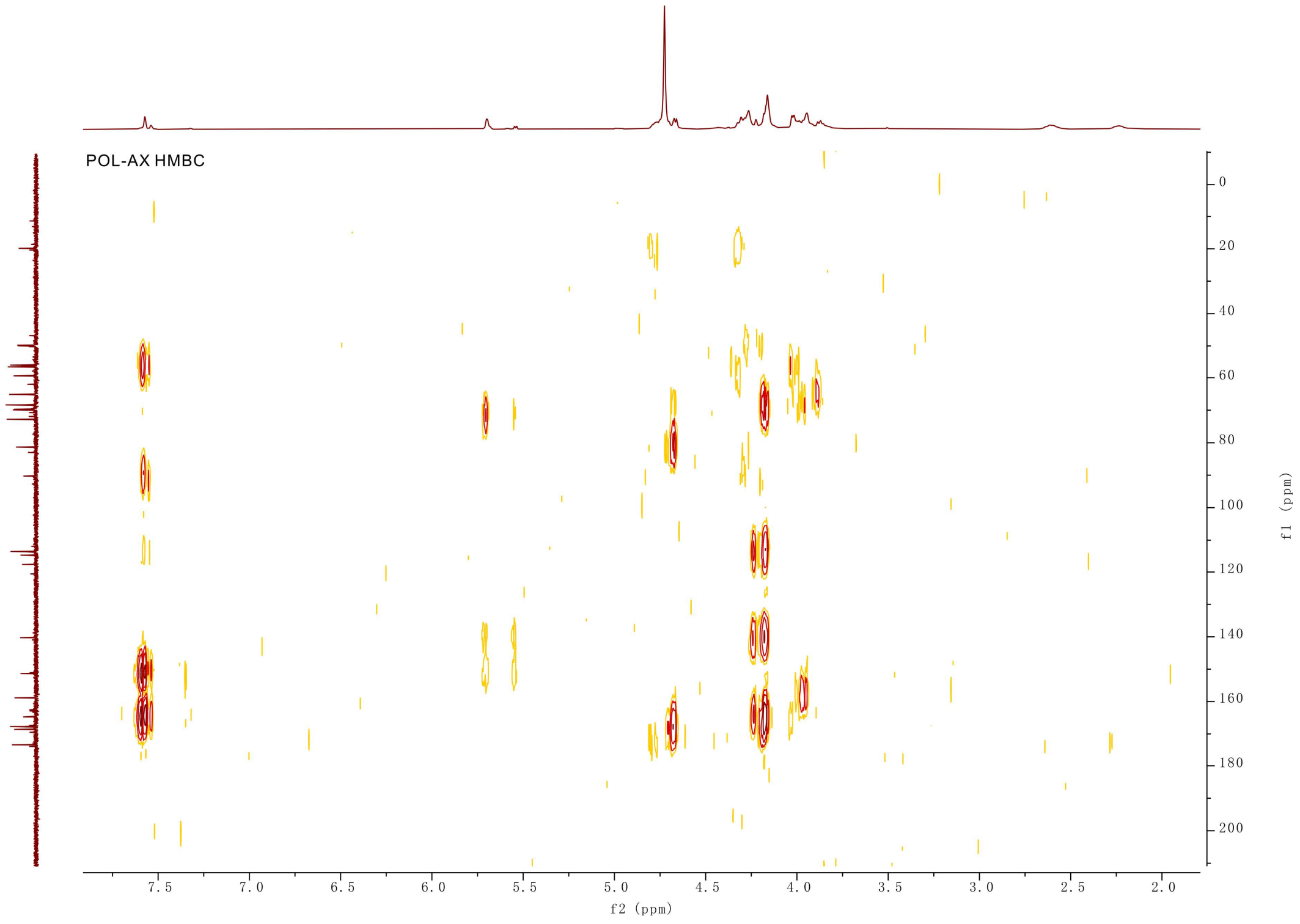

Figure S15. HMBC spectrum of POL-Ax (D_2O, 400 MHz).

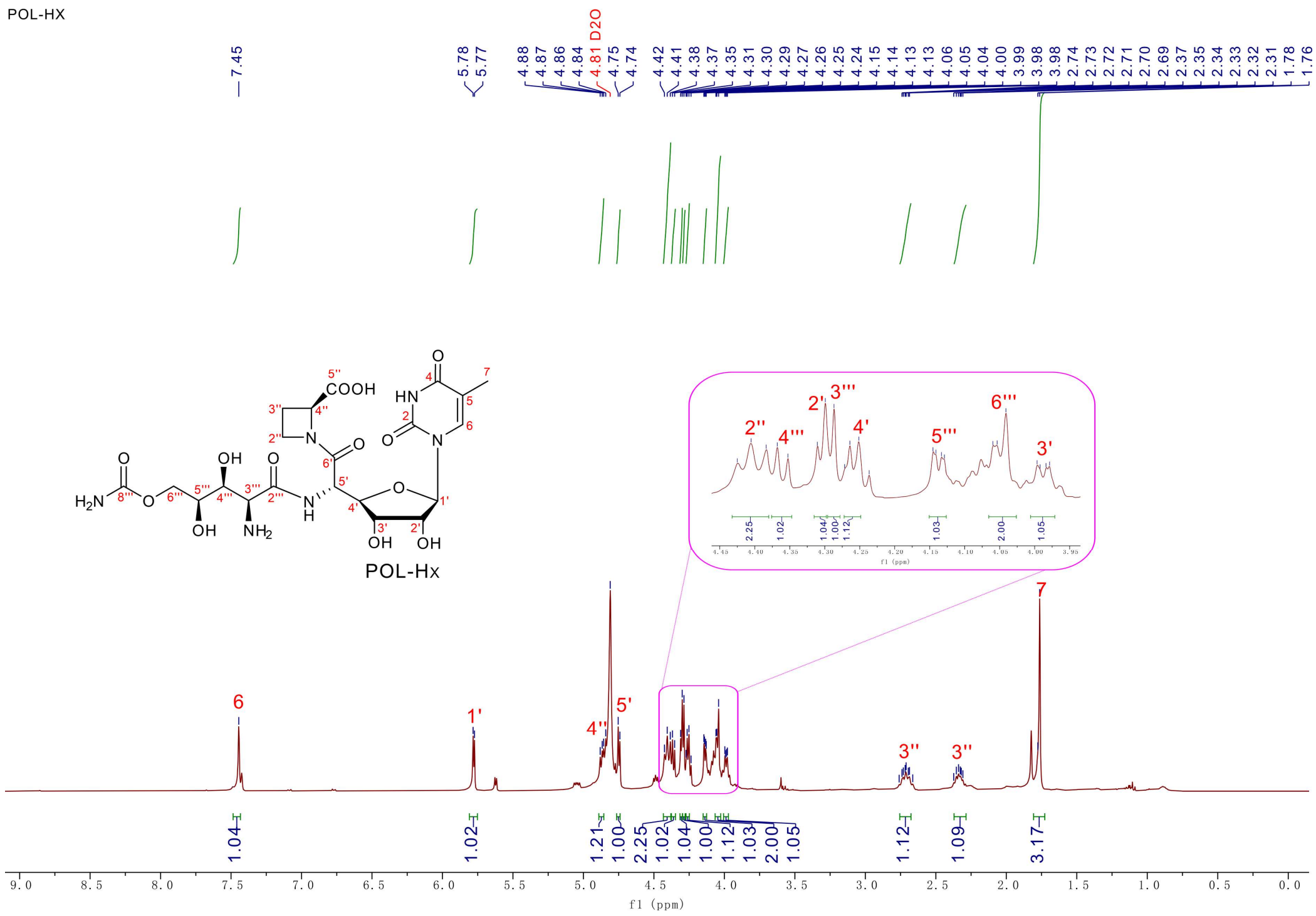

Figure S16. ^{1}H NMR spectrum of POL-Hx (D_2O, 400 MHz).

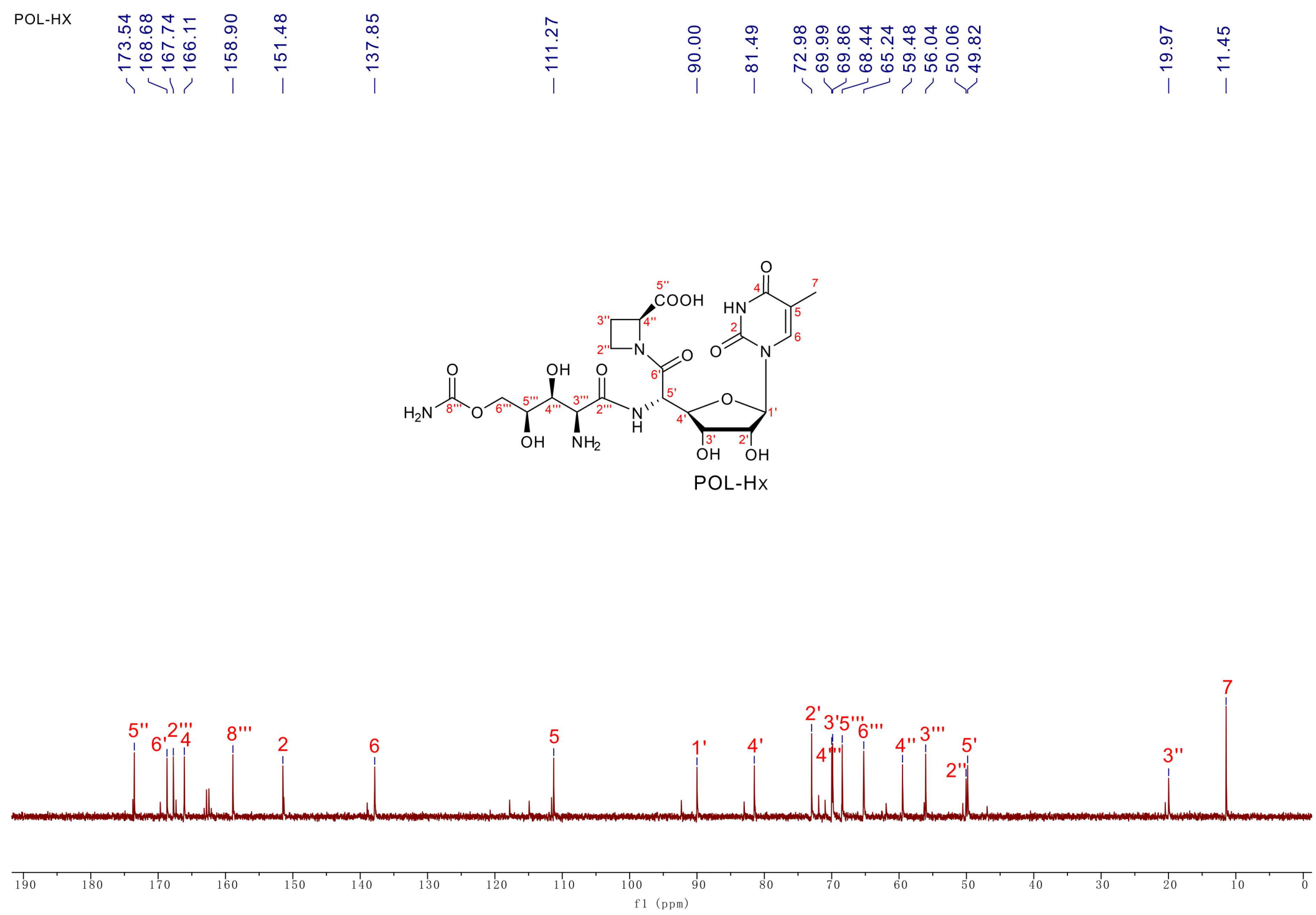

Figure S17. ^{13}C NMR spectrum of POL-Hx (D_2O, 100 MHz).

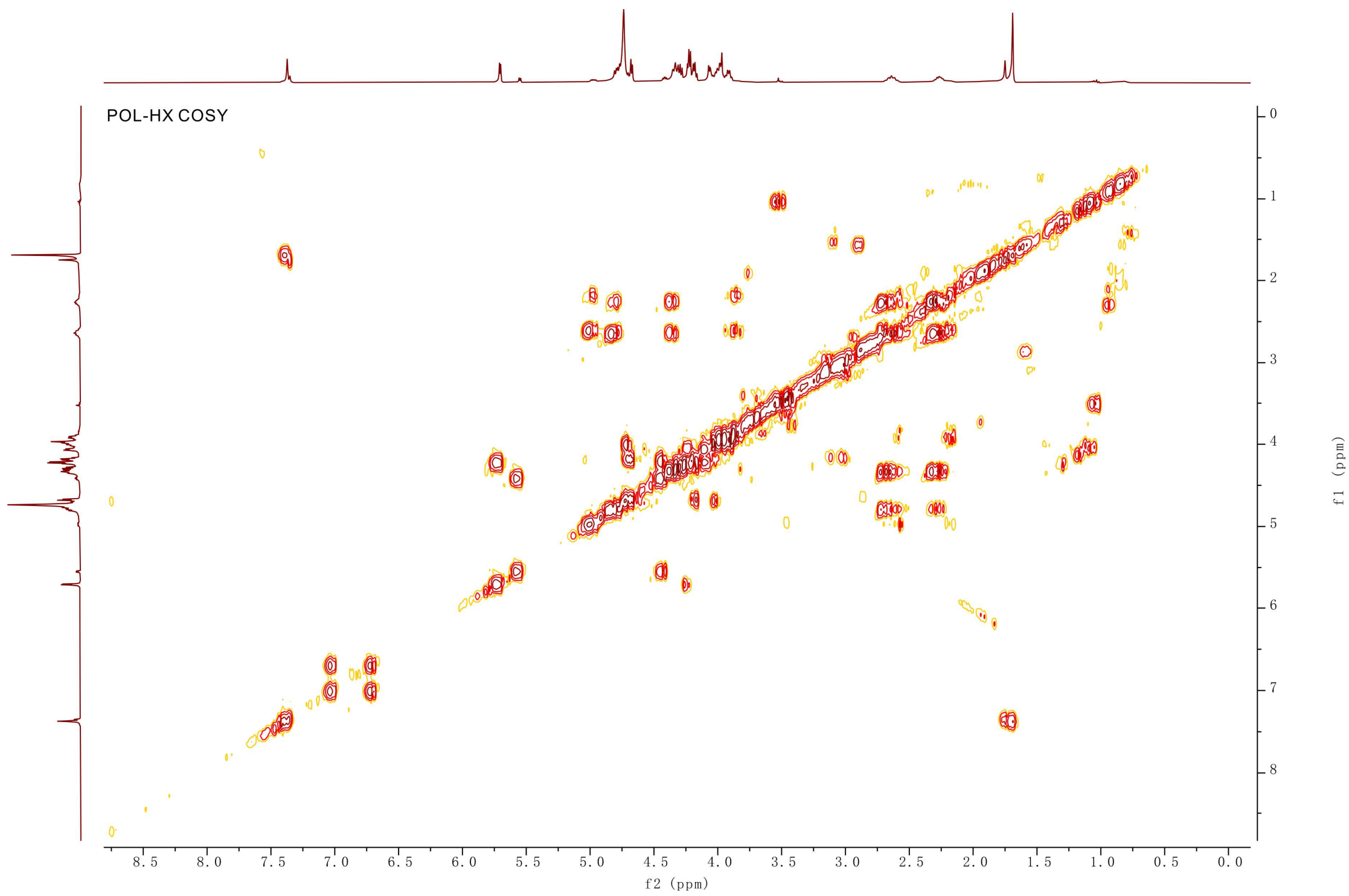

Figure S18. ^{1}H-^{1}H COSY spectrum of POL-Hx (D_2O, 400 MHz).

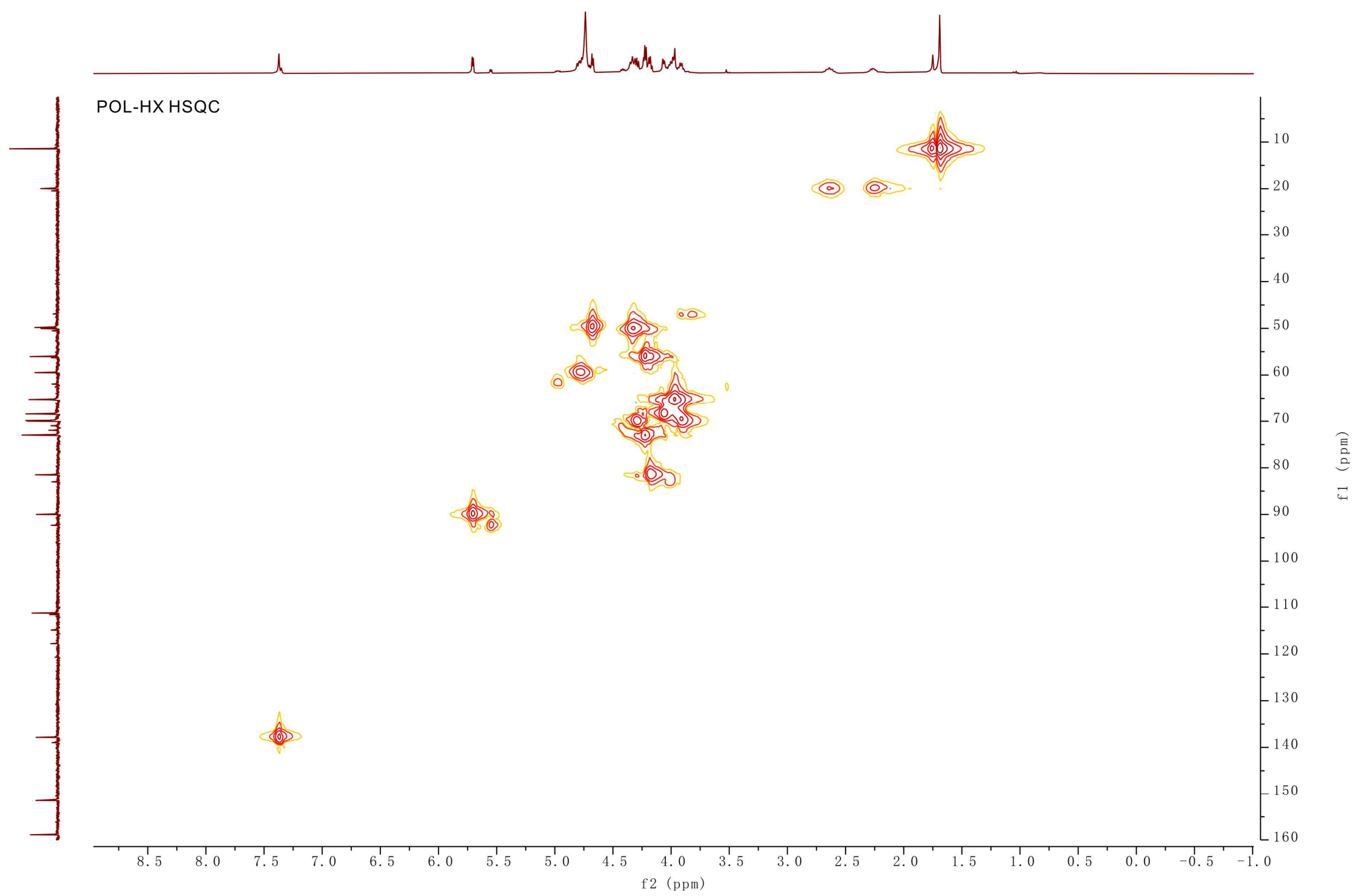

Figure S19. HSQC spectrum of POL-Hx (D_2O, 400 MHz).

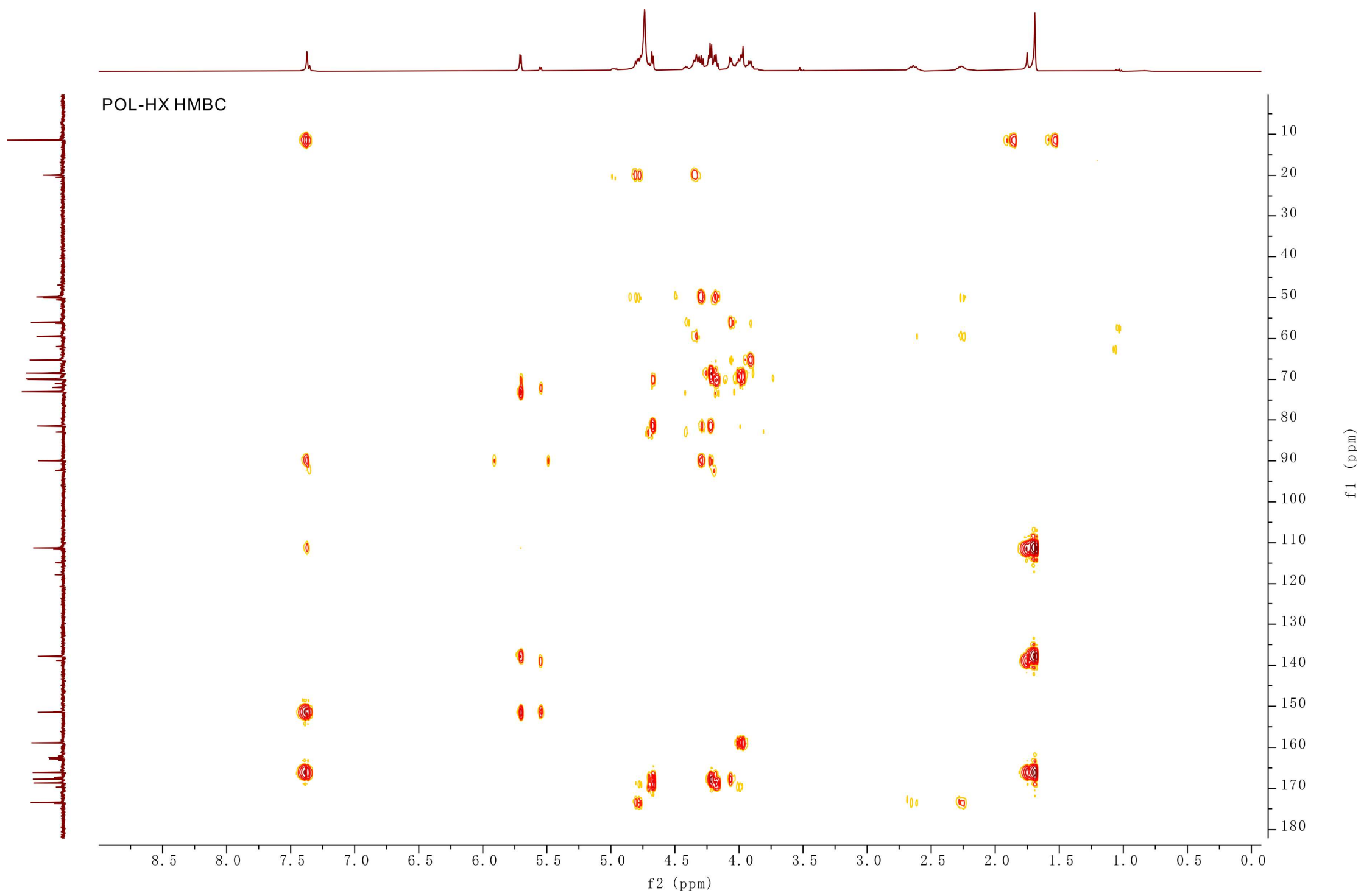

Figure S20. HMBC spectrum of POL-Hx (D_2O, 400 MHz).

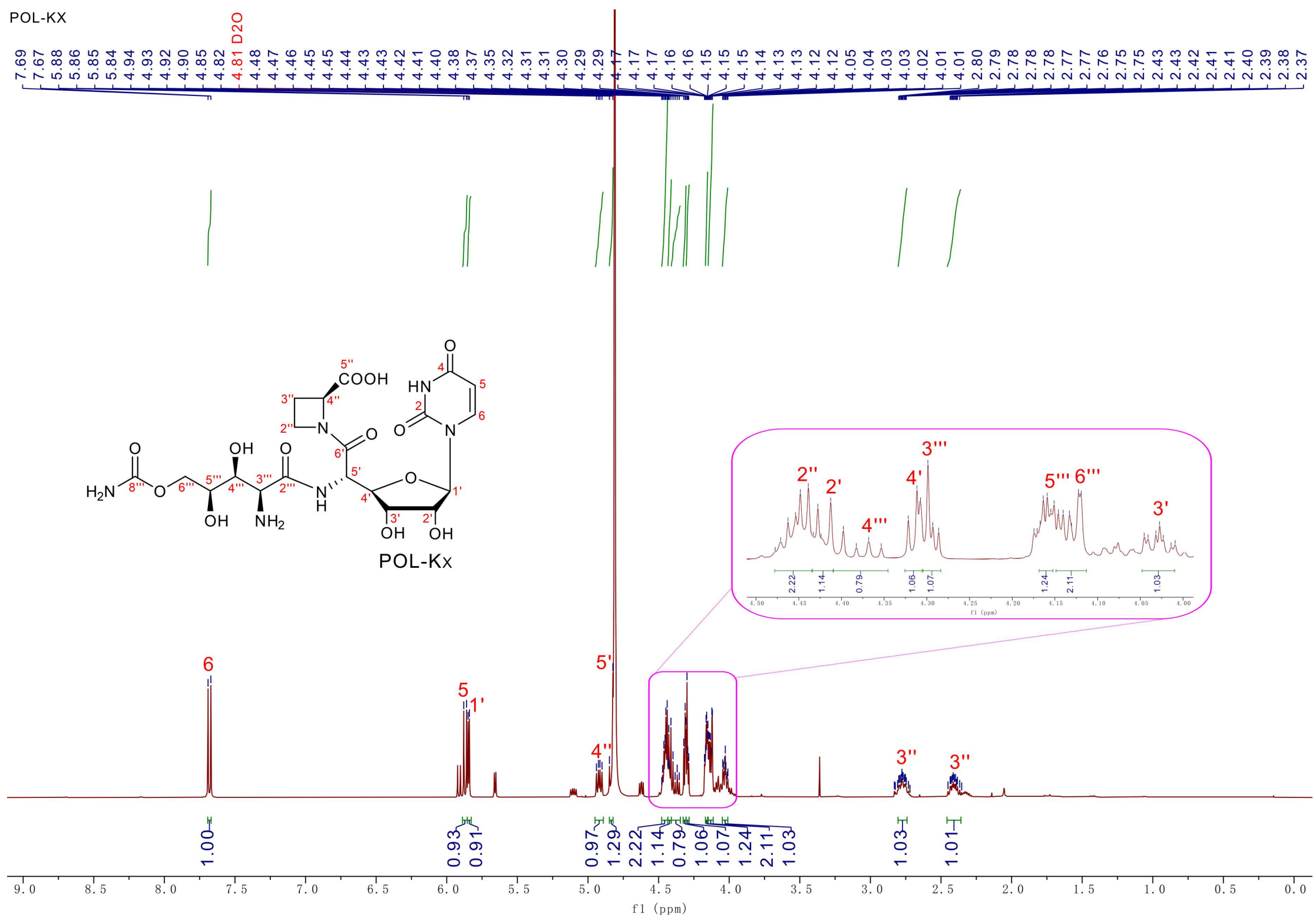

Figure S21. ^{1}H NMR spectrum of POL-Kx (D_2O, 600 MHz).

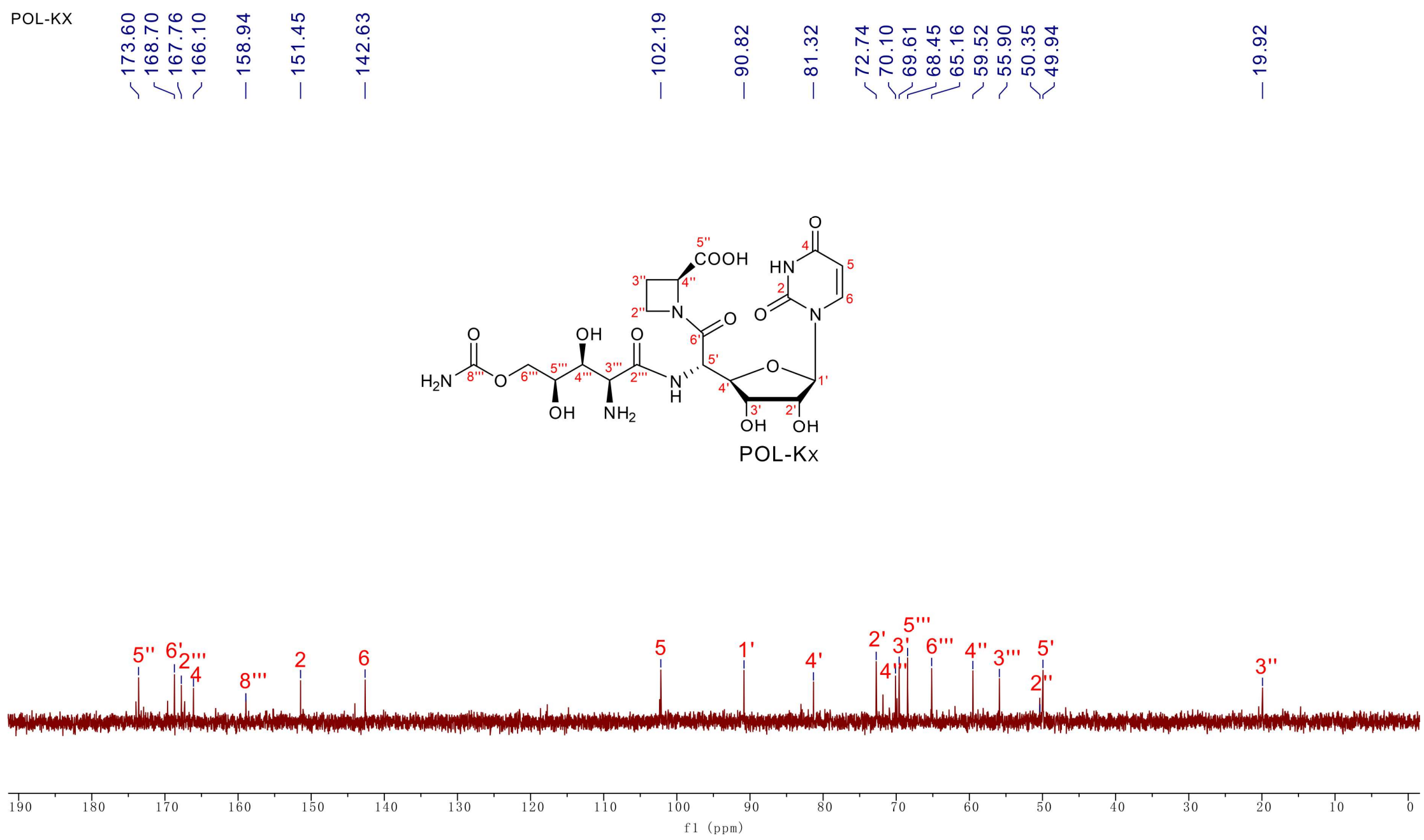

Figure S22. ^{13}C NMR spectrum of POL-Kx (D_2O, 150 MHz).

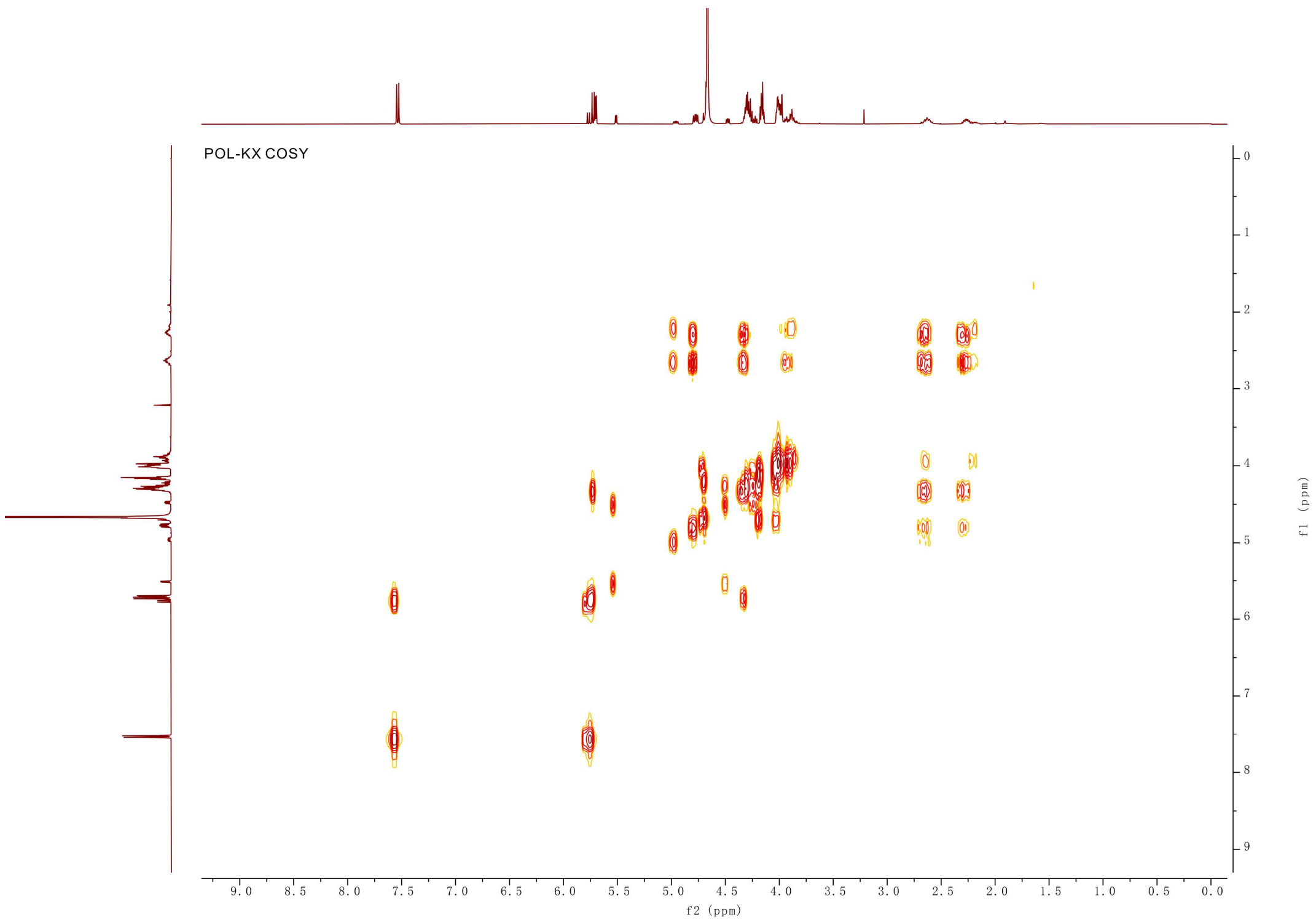

Figure S23. ^{1}H-^{1}H COSY spectrum of POL-K$_X$ (D_2O, 600 MHz).

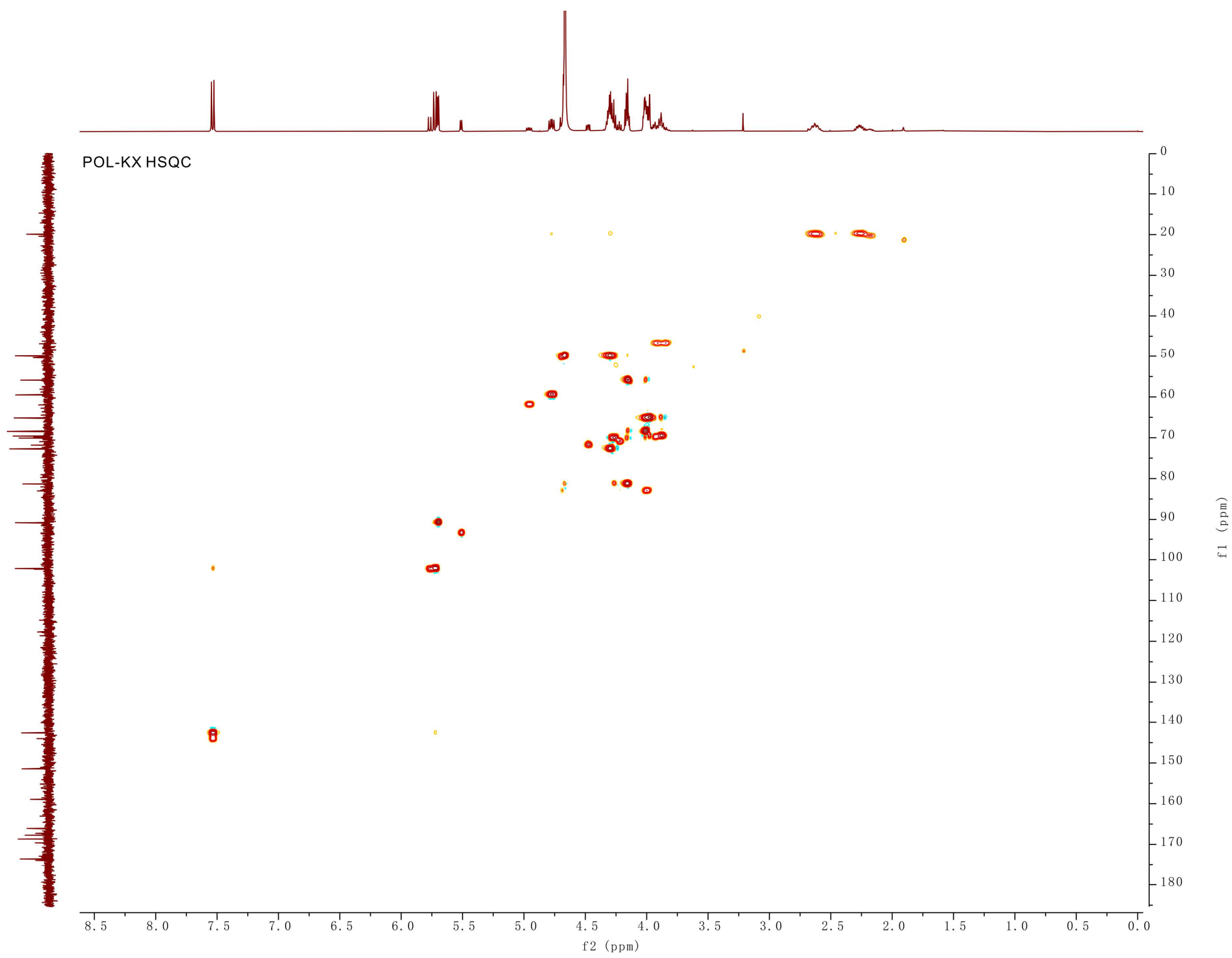

Figure S24. HSQC spectrum of POL-K$_X$ (D_2O, 600 MHz).

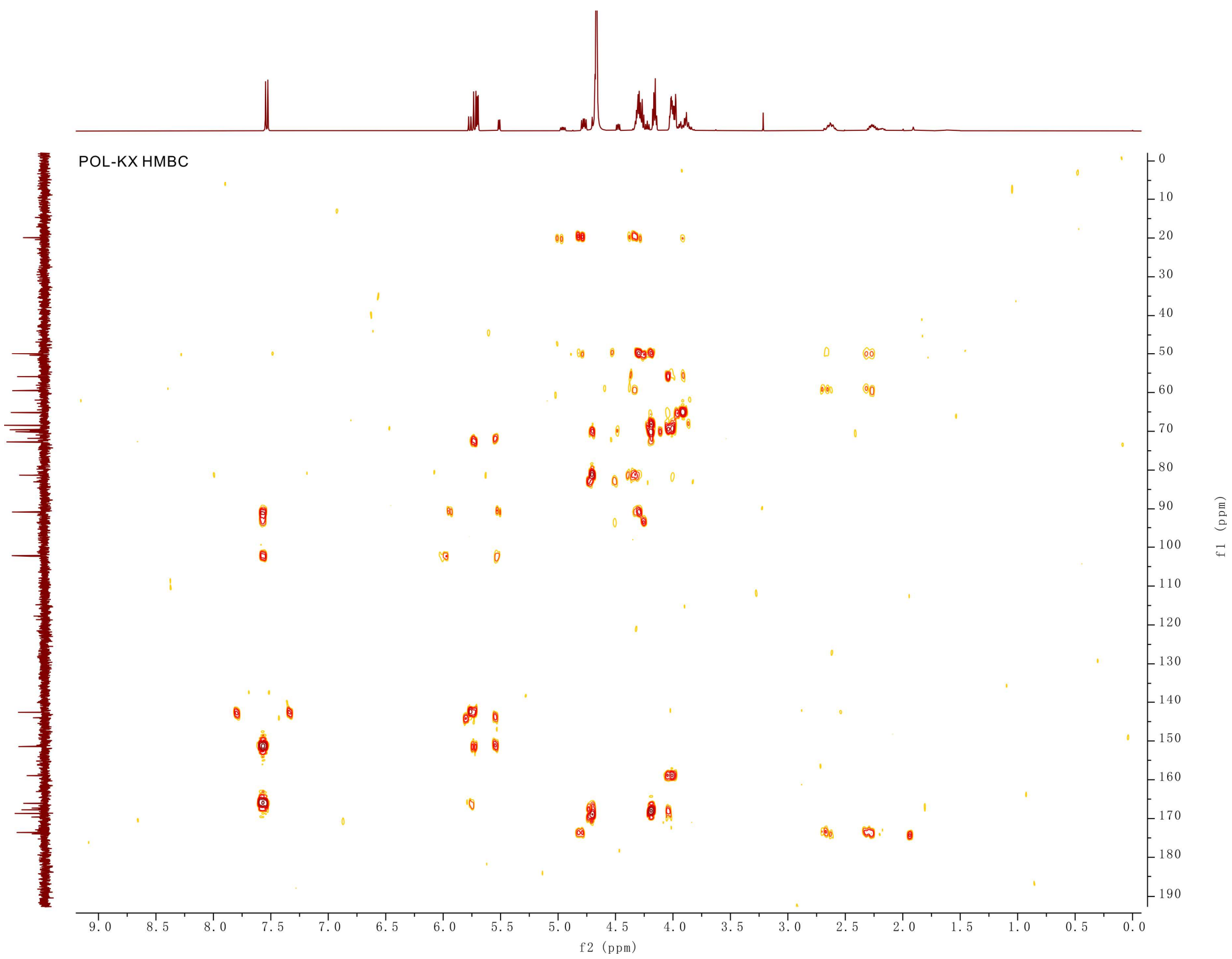

Figure S25. HMBC spectrum of POL-KX (D_2O, 600 MHz).

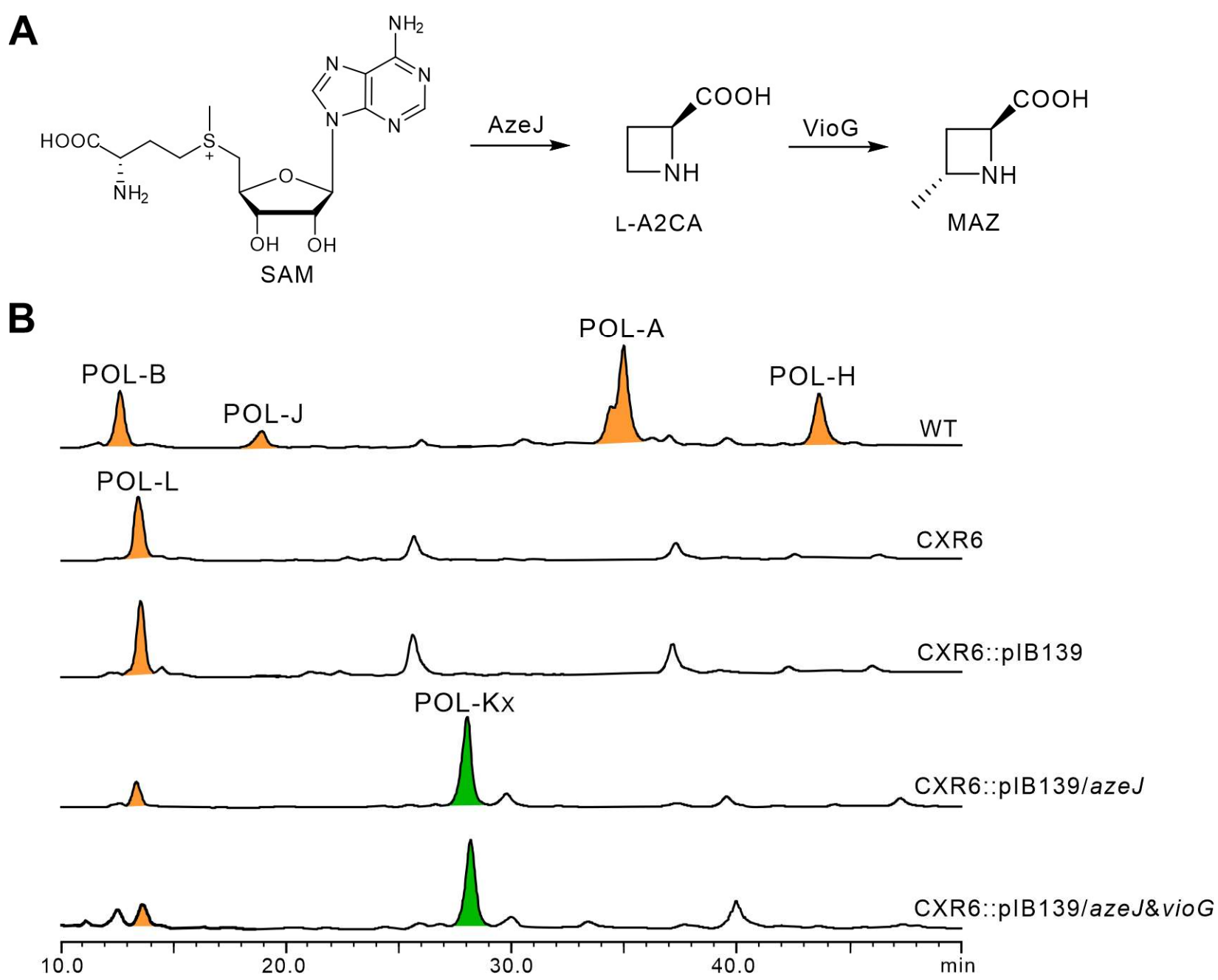

Figure S26. HPLC analysis of the target metabolites produced by the recombinant CXR6::pIB139/*azeJ&vioG*. (A) The VioG protein (Accession no. AWI62632, a SAM-dependent methyltransferase from *Myxococcus xanthus*)[11, 12], was confirmed to catalyze L-A2CA to MAZ. MAZ, (2*S*, 4*R*)-4-methylazetidine-2-carboxylic acid. (B) HPLC analysis of the target metabolites produced by the recombinant CXR6::pIB139/*azeJ&vioG*.

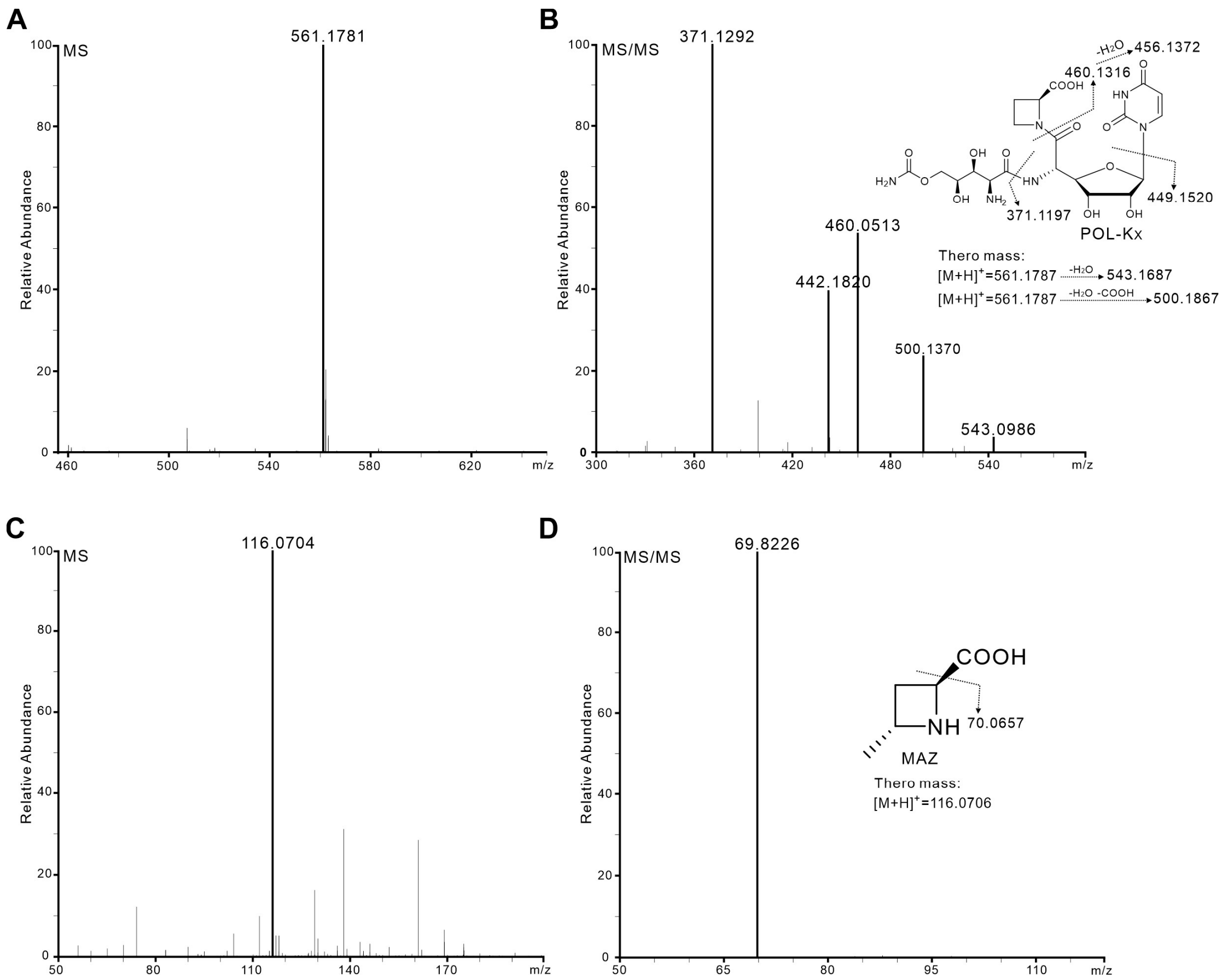

Figure S27. LC-HRMS analysis of the target metabolites produced by the recombinant CXR6::pIB139/*azeJ&vioG*. (A) LC-HRMS analysis of the target metabolite POL-K$_X$ produced by the recombinant CXR6::pIB139/*azeJ&vioG*. (B) LC-HRMS/MS analysis of the target metabolite POL-K$_X$ produced by the recombinant CXR6::pIB139/*azeJ&vioG*. The theoretical fragmentation patterns of POL-K$_X$ is included as control. (C) LC-HRMS analysis of MAZ produced by recombinant CXR6::pIB139/*azeJ&vioG*. (D) LC-HRMS/MS analysis of MAZ produced by recombinant CXR6::pIB139/*azeJ&vioG*. The theoretical fragmentation patterns of MAZ is included as a control.

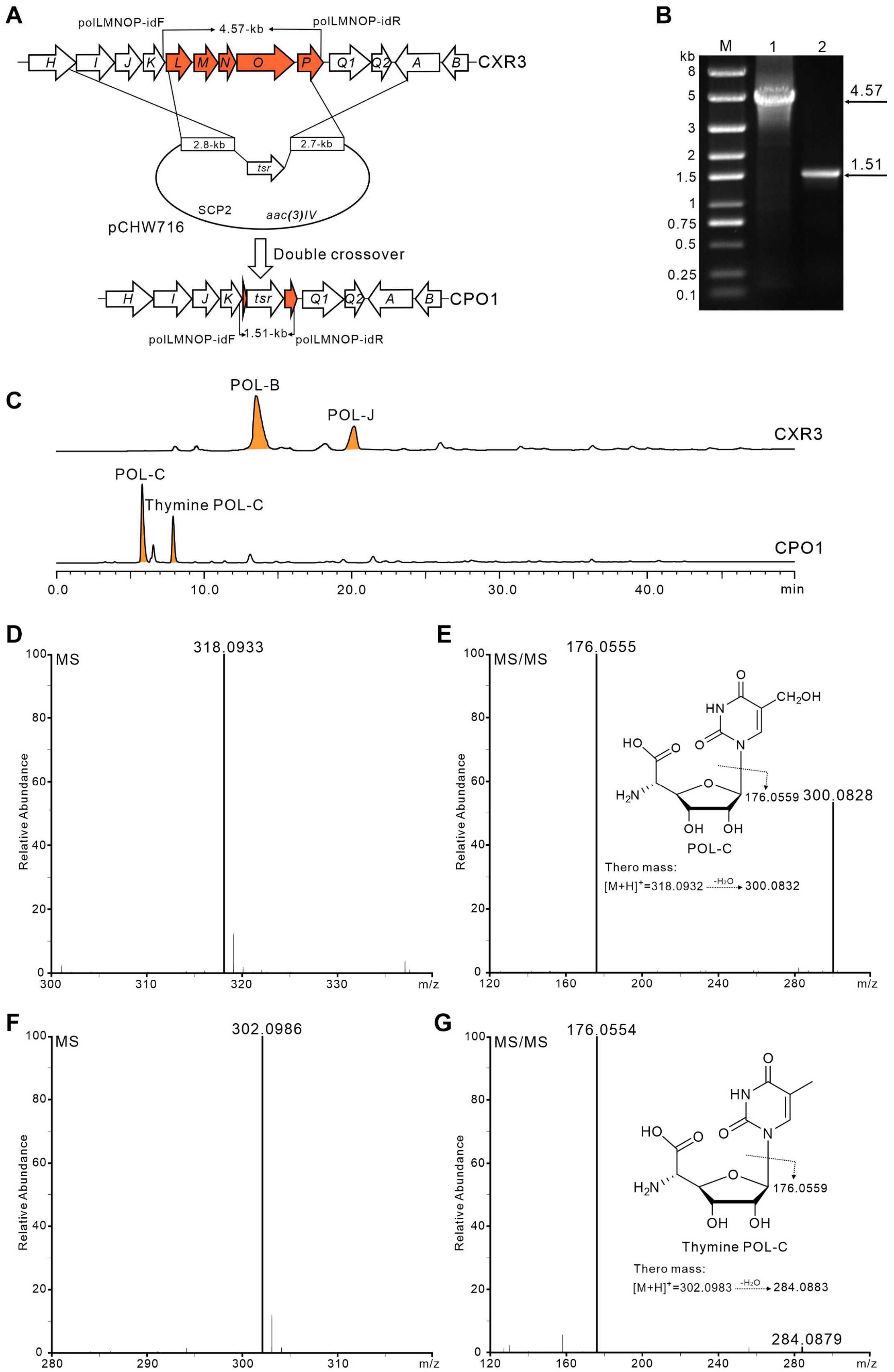

Figure S28. Construction and metabolites analysis of the strain CPO1. (A) Schematic illustration for the construction of the strain CPO1. (B) PCR identification

of the strain CPO1. M, 1 kb ladder; 1, PCR product was amplified using genomic DNA of CXR3 as template. The expected size of the PCR product was 4.57 kb; 2, PCR product using genomic DNA of CPO1 as template. The expected size of the PCR product was 1.51 kb. (C) HPLC analysis of the target metabolites separately produced by the strains CXR3 and CPO1. (D) LC-HRMS analysis of the target metabolite POL-C produced by the strain CPO1. (E) LC-HRMS/MS analysis of the target metabolite POL-C produced by the strain CPO1. The theoretical fragmentation pattern of POL-C is included as a control. (F) LC-HRMS analysis of the target metabolite thymine POL-C produced by the strain CPO1. (G) LC-HRMS/MS analysis of the target metabolite thymine POL-C produced by the strain CPO1. The theoretical fragmentation pattern of thymine POL-C is included as a control.

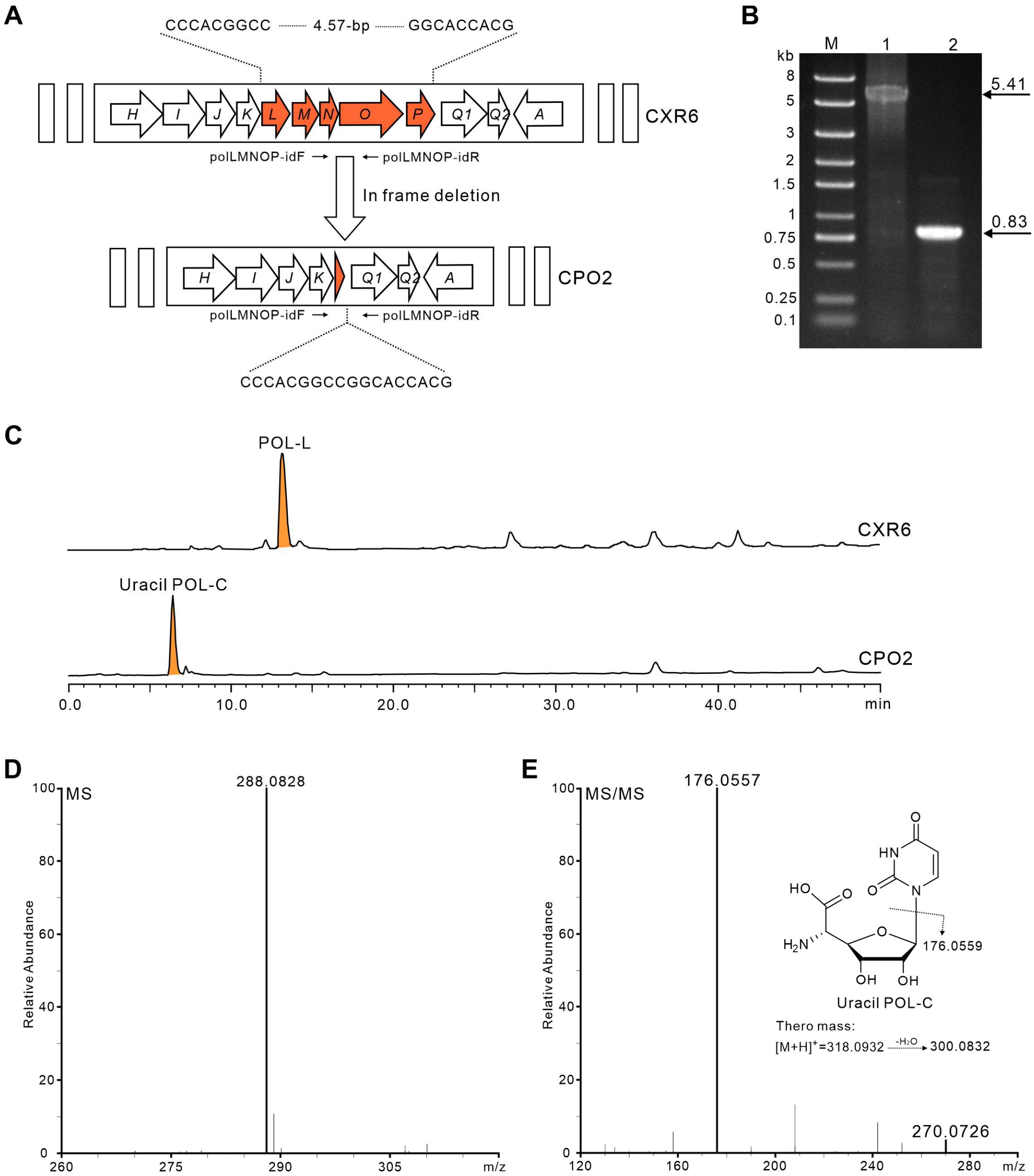

Figure S29. Construction and metabolites analysis of the strain CPO2. (A) Schematic illustration for the construction of the strain CPO2. (B) PCR identification of CPO2. M, 1 kb ladder; 1, PCR product was amplified using genomic DNA of CXR6 as template. The expected size of the PCR product was 5.41 kb; 2, PCR product was amplified using genomic DNA of CPO2 as template. The expected size of the PCR product was 0.83 kb. (C) HPLC analysis of the target metabolites separately produced by the strains CXR6 and CPO2. (D) LC-HRMS analysis of the target metabolite uracil POL-C produced by the strain CPO2. (E) LC-HRMS analysis of the target metabolite uracil POL-C produced by the strain CPO2. The theoretical fragmentation pattern of uracil POL-C is indicated as a control.

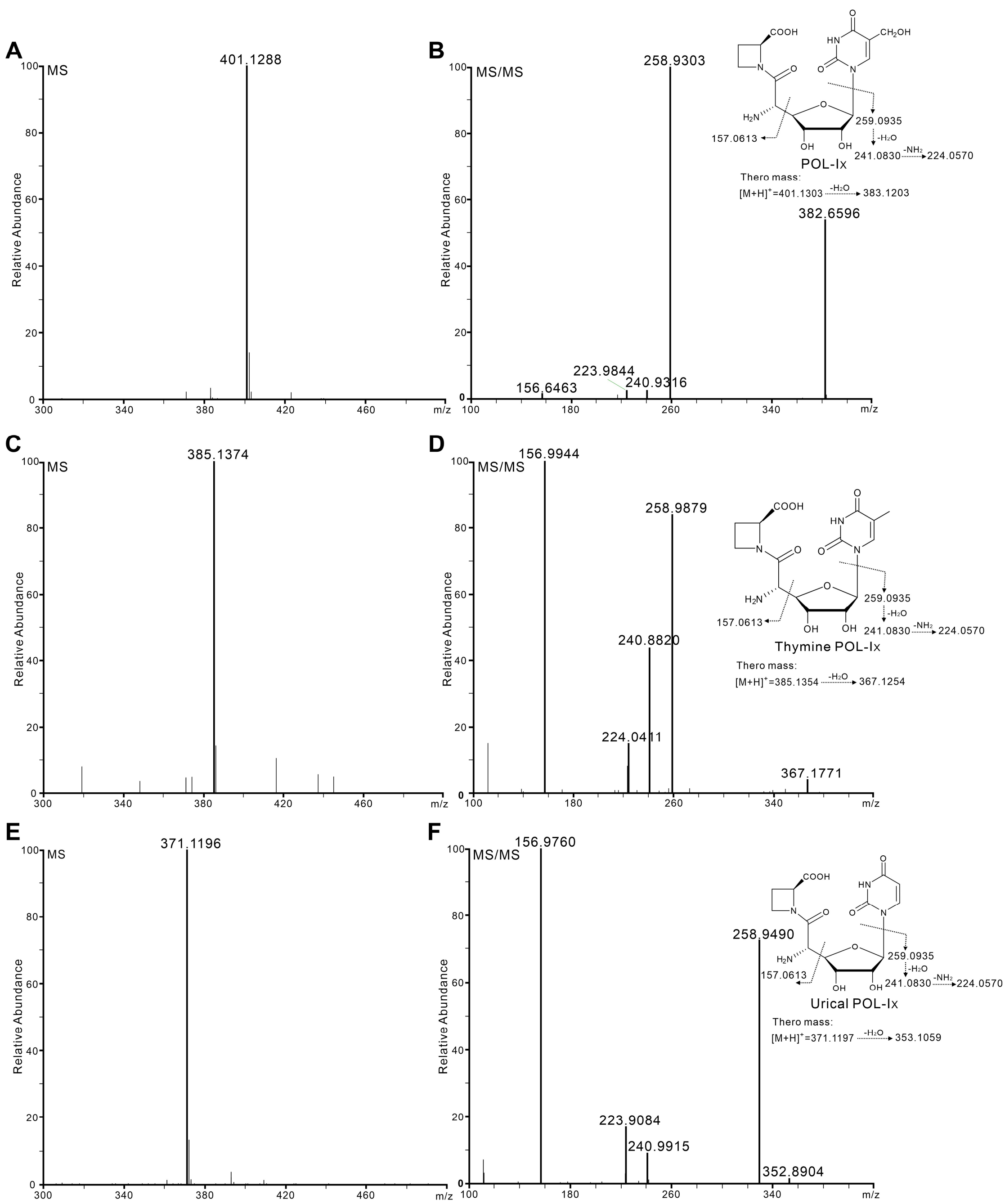

Figure S30. LC-HRMS analysis of the target metabolites individually produced by the recombinants CPO1::pIB139/*azeJ* and CPO2::pIB139/*azeJ*. (A) LC-HRMS analysis of the target metabolite POL-I$_X$ produced by the recombinant CPO1::pIB139/*azeJ*. (B) LC-HRMS/MS analysis of the target metabolite POL-I$_X$ produced by the recombinant CPO1::pIB139/*azeJ*. The theoretical fragmentation pattern of POL-I$_X$ is included as a control. (C) LC-HRMS analysis of the target metabolite thymine POL-I$_X$ produced by CPO1::pIB139/*azeJ*. (D) LC-HRMS/MS analysis of the target metabolite thymine POL-I$_X$ produced by CPO1::pIB139/*azeJ*. The

theoretical fragmentation pattern of thymine POL-I_X is included as a control. (E) LC-HRMS analysis of the target metabolite uracil POL-I_X produced by the strain CPO2::pIB139/*azeJ*. (F) LC-HRMS/MS analysis of the target metabolite uracil POL-I_X produced by the strain CPO2::pIB139/*azeJ*. The theoretical fragmentation pattern of uracil POL-I_X is also included as a control.

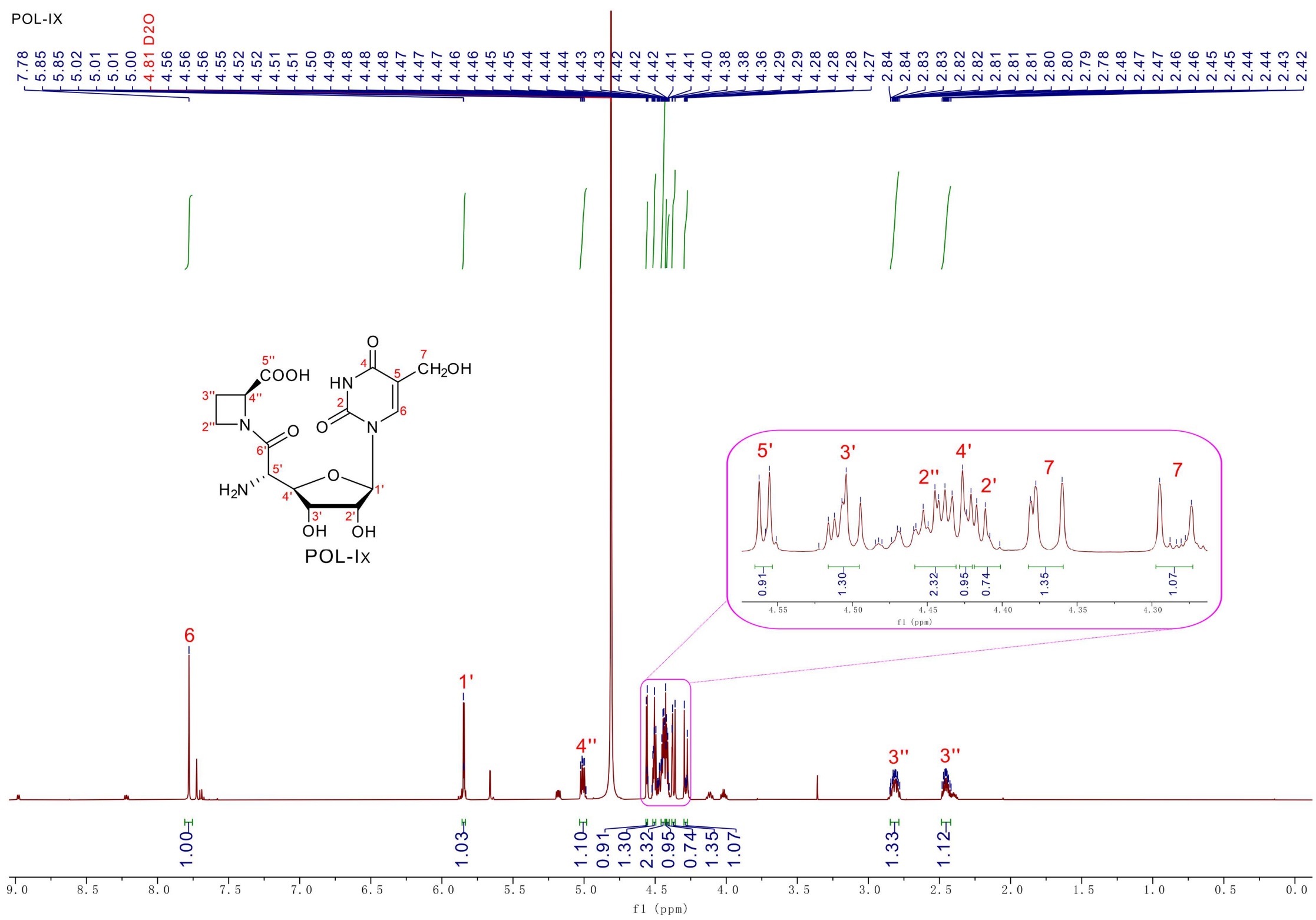

Figure S31. [1]**H NMR spectrum of POL-Ix (**D_2O**, 600 MHz).**

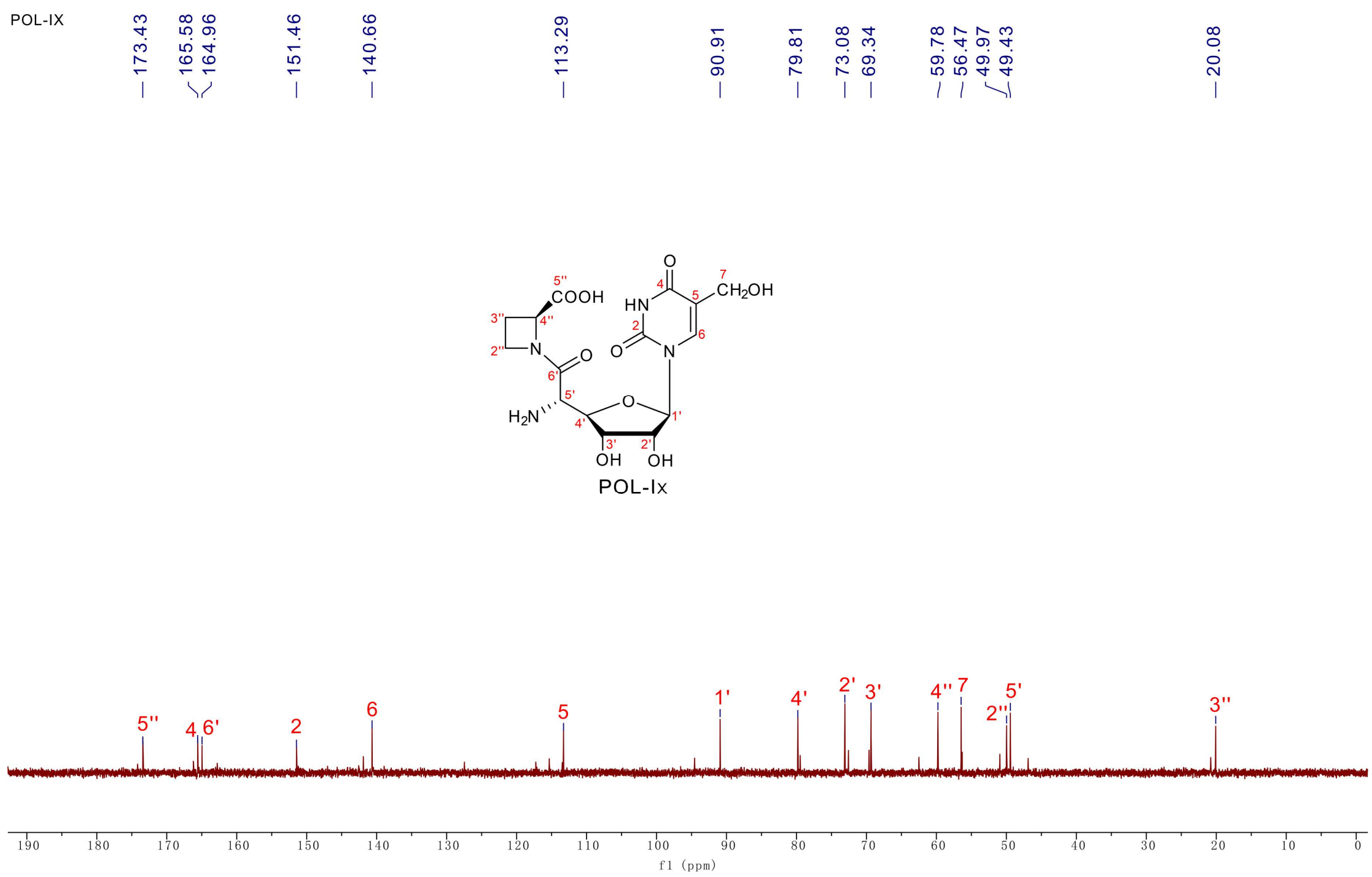

Figure S32. [13]**C NMR spectrum of POL-Ix (**D_2O**, 150 MHz).**

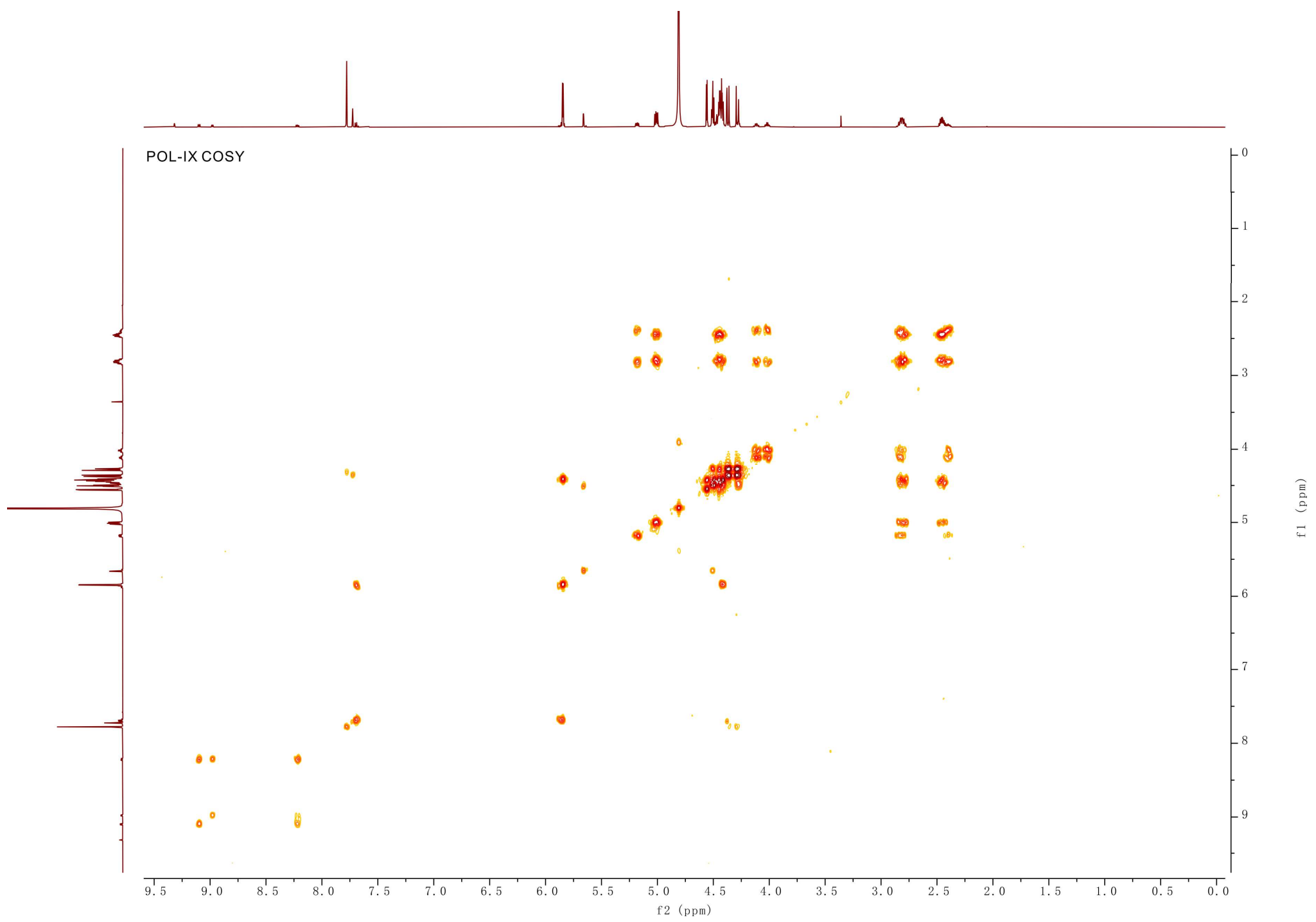

Figure S33. **^{1}H-^{1}H COSY NMR spectrum of POL-Ix (D_2O, 600 MHz).**

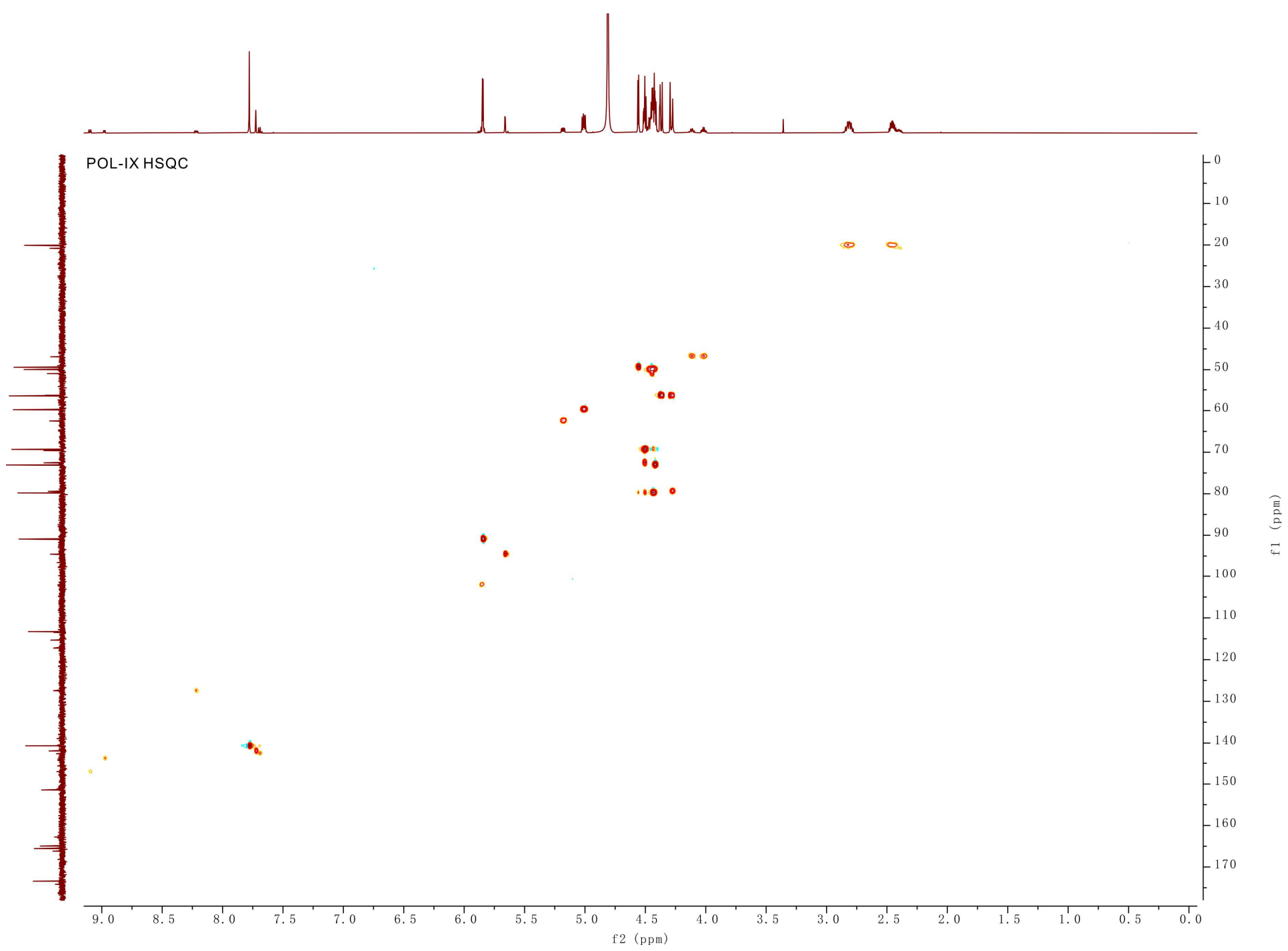

Figure S34. HSQC NMR spectrum of POL-Ix (D_2O, 600 MHz).

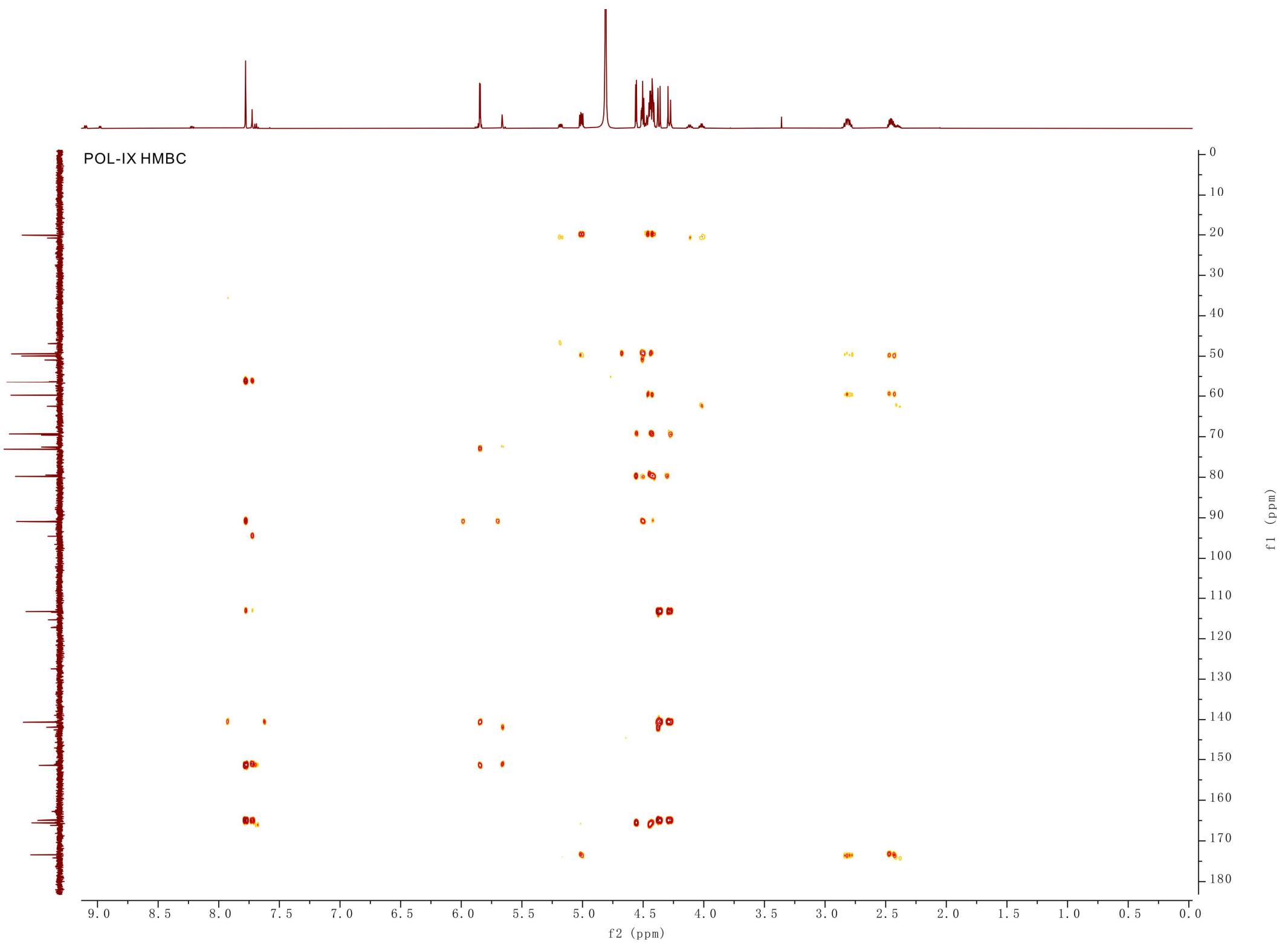

Figure S35. HMBC NMR spectrum of POL-Ix (D_2O, 600 MHz).

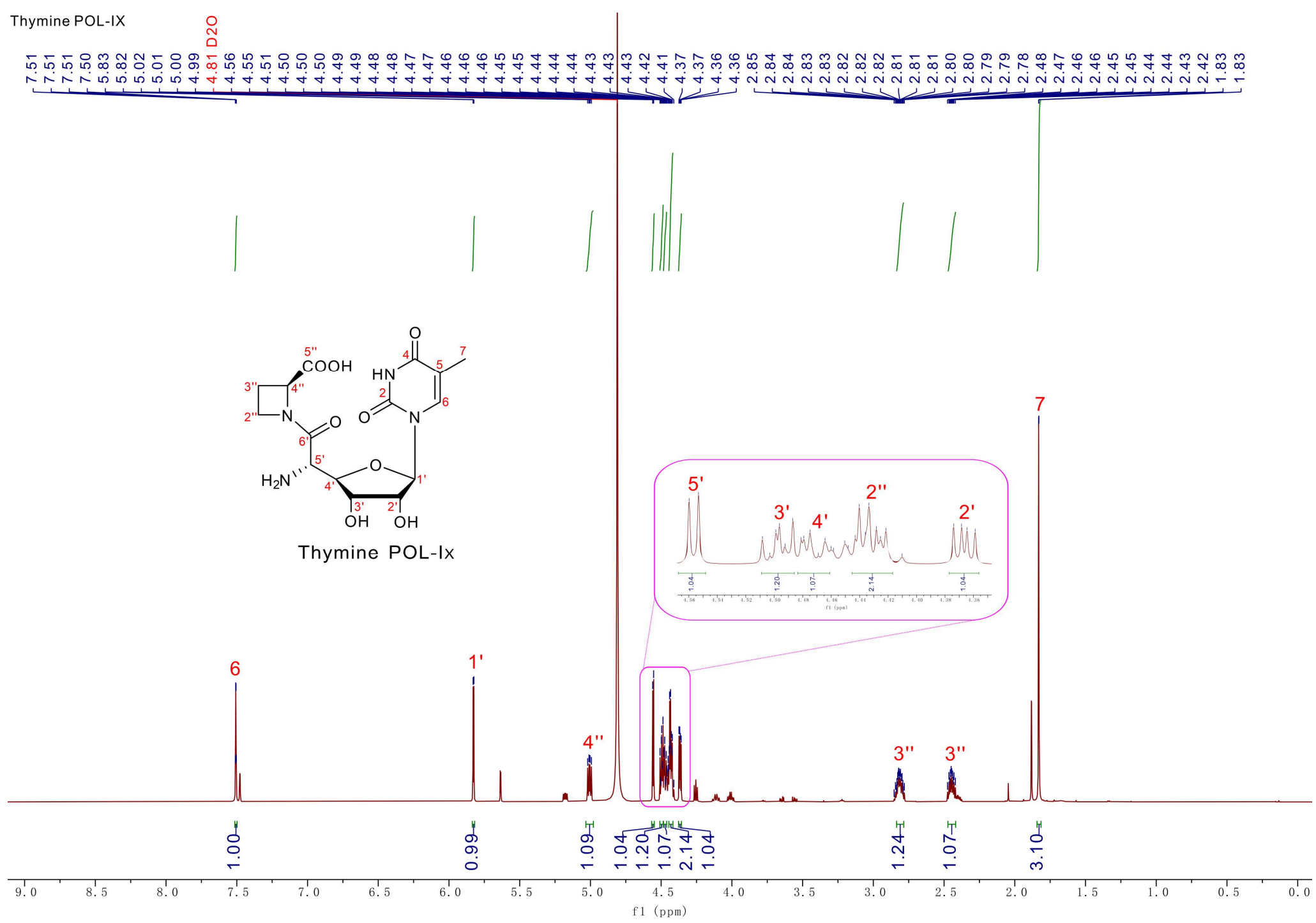

Figure S36. ^{1}H NMR spectrum of thymine POL-Ix (D_2O, 600 MHz).

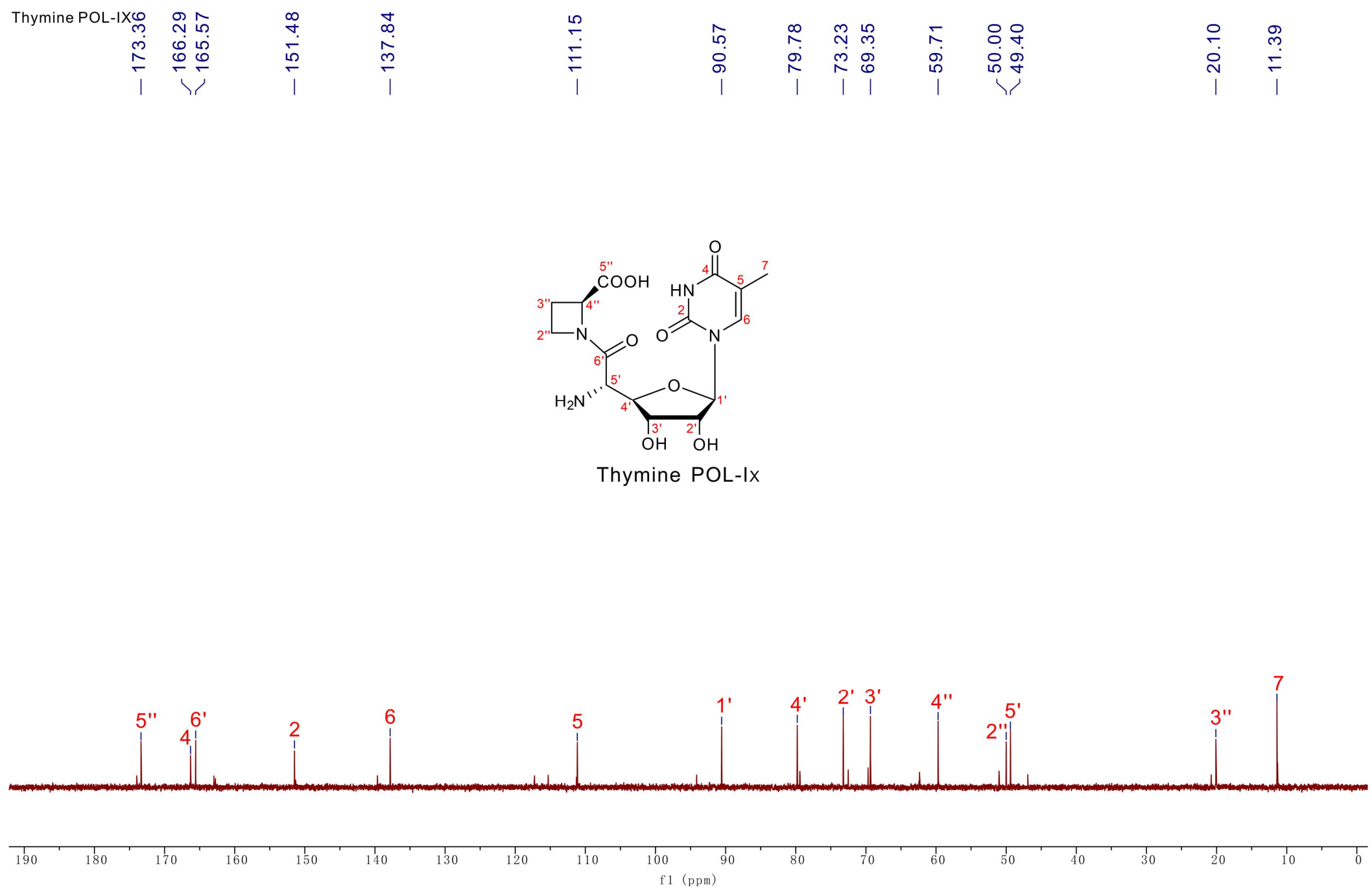

Figure S37. ^{13}**C NMR spectrum of thymine POL-I**$_X$ **(**D_2O**, 150 MHz).**

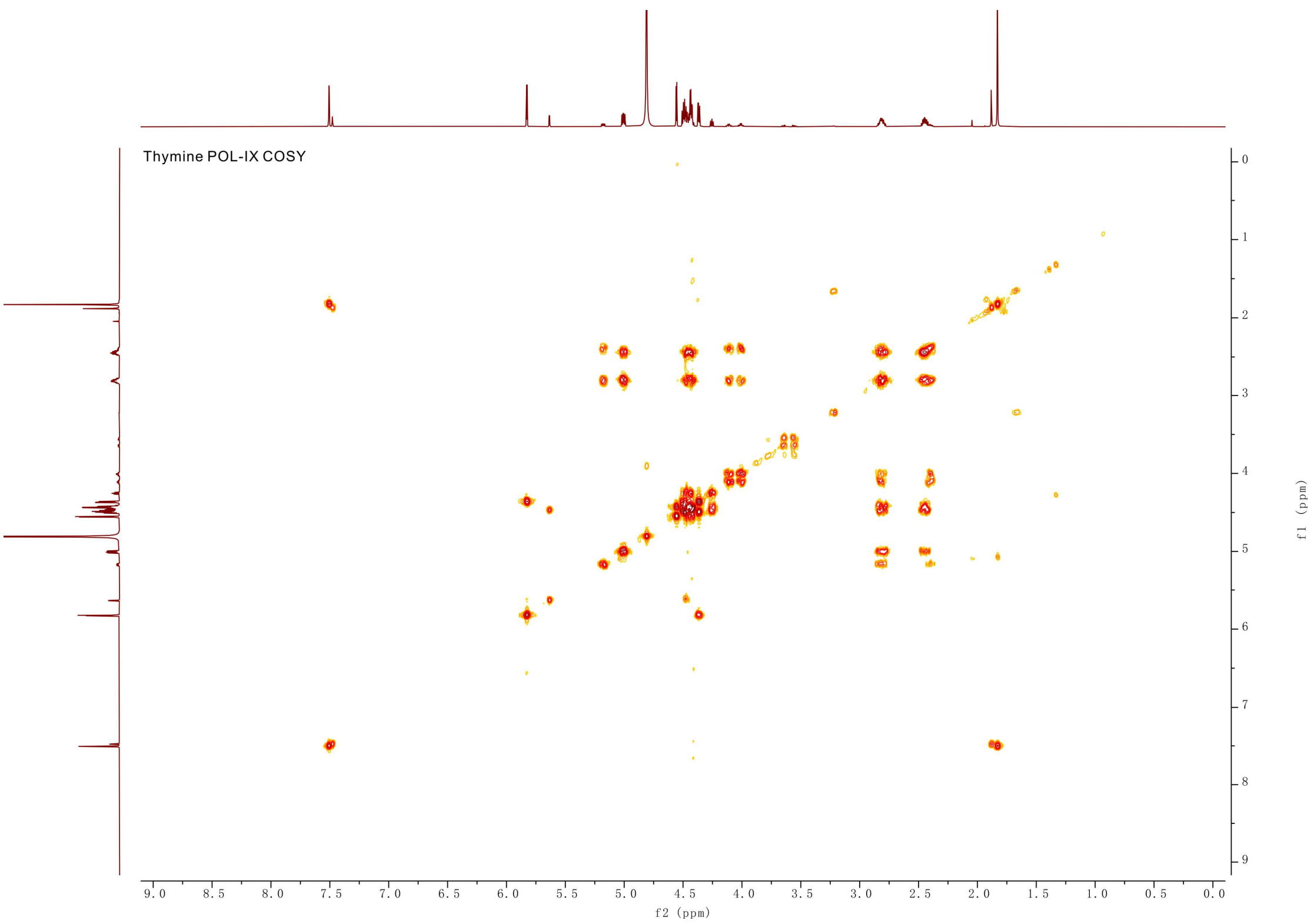

Figure S38. ^{1}**H-**^{1}**H COSY NMR spectrum of thymine POL-I**$_X$ **(**D_2O**, 600 MHz).**

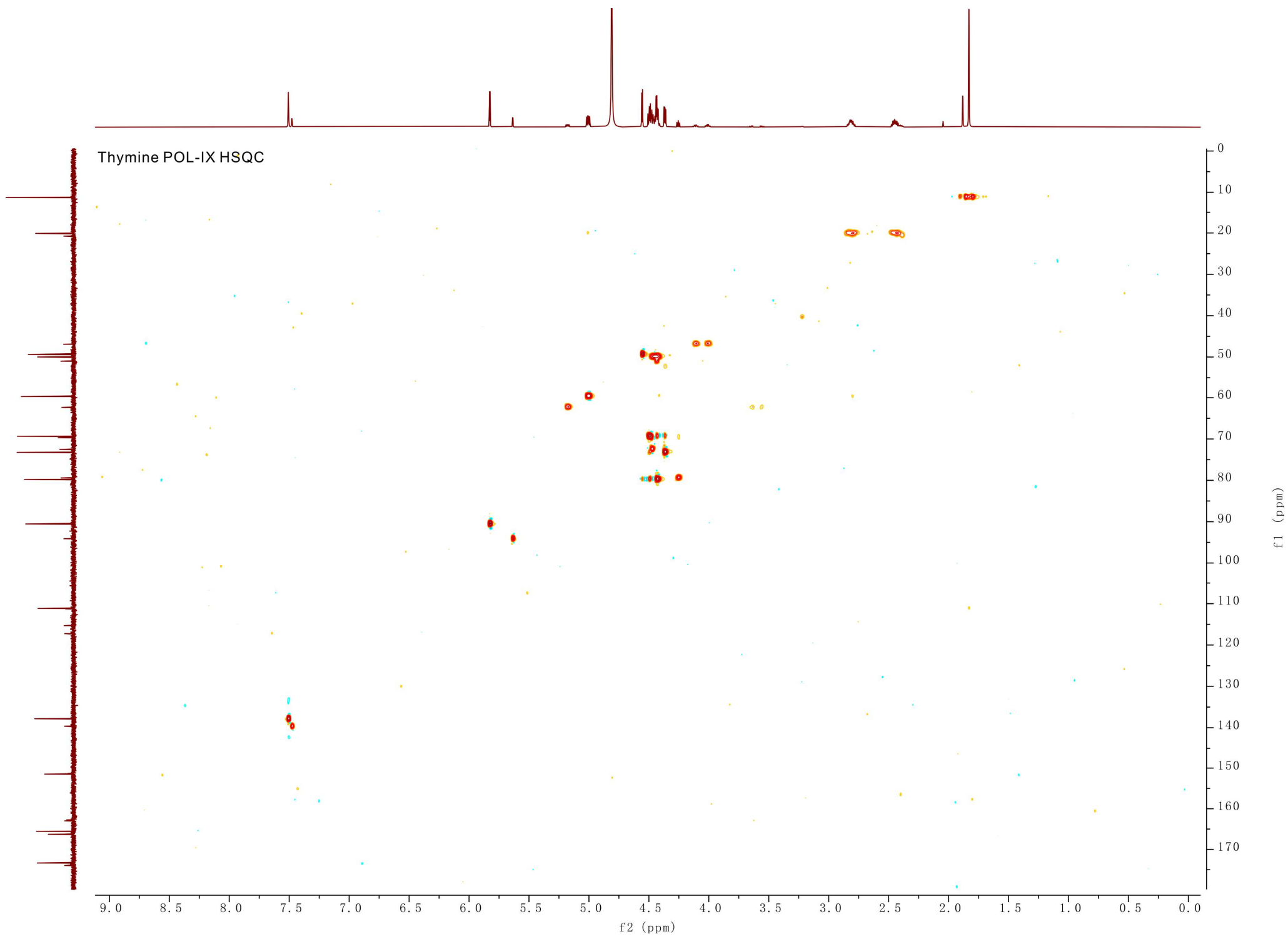

Figure S39. HSQC NMR spectrum of thymine POL-Ix (D$_2$O, 600 MHz).

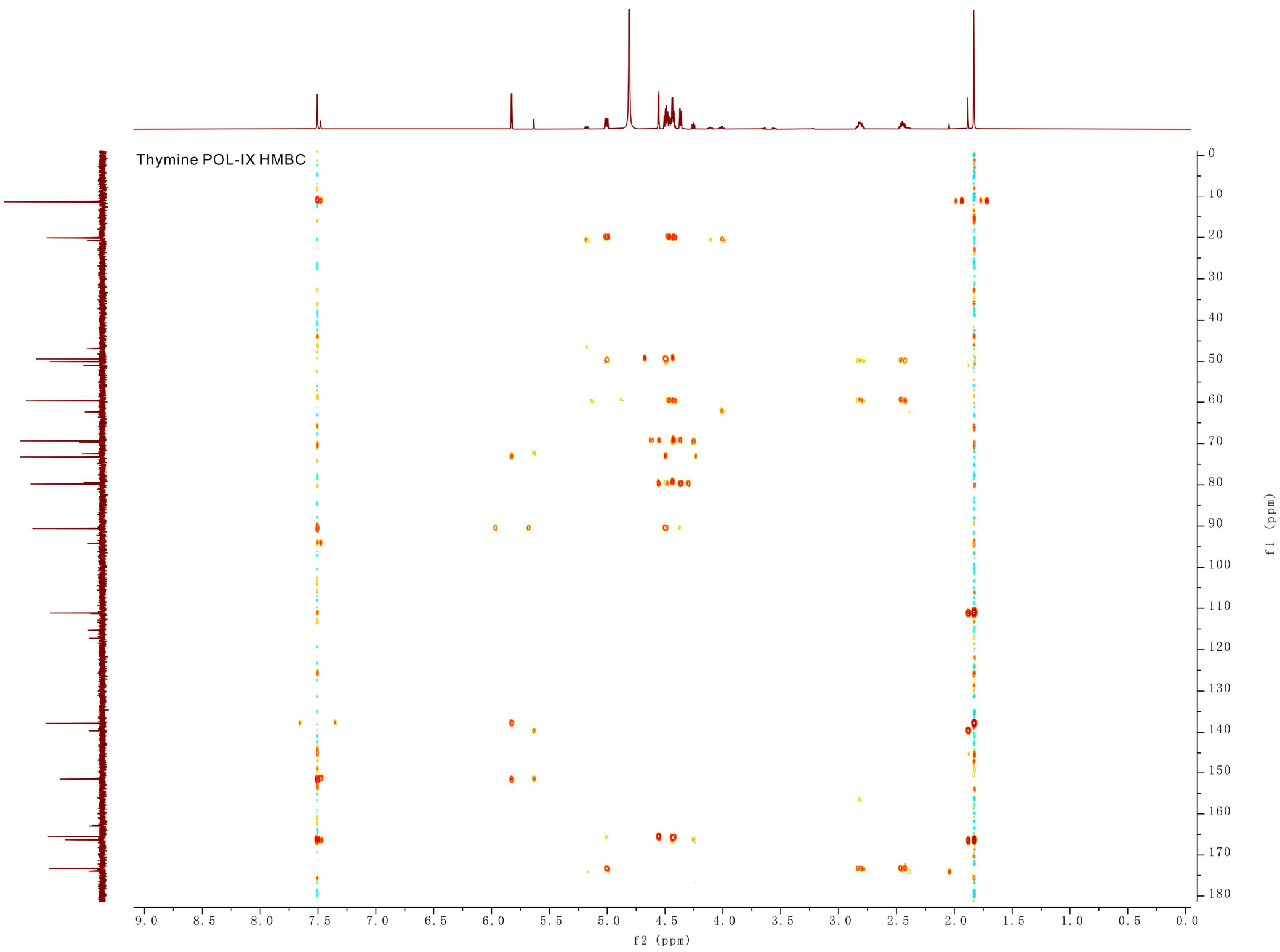

Figure S40. HMBC NMR spectrum of thymine POL-Ix (D$_2$O, 600 MHz).

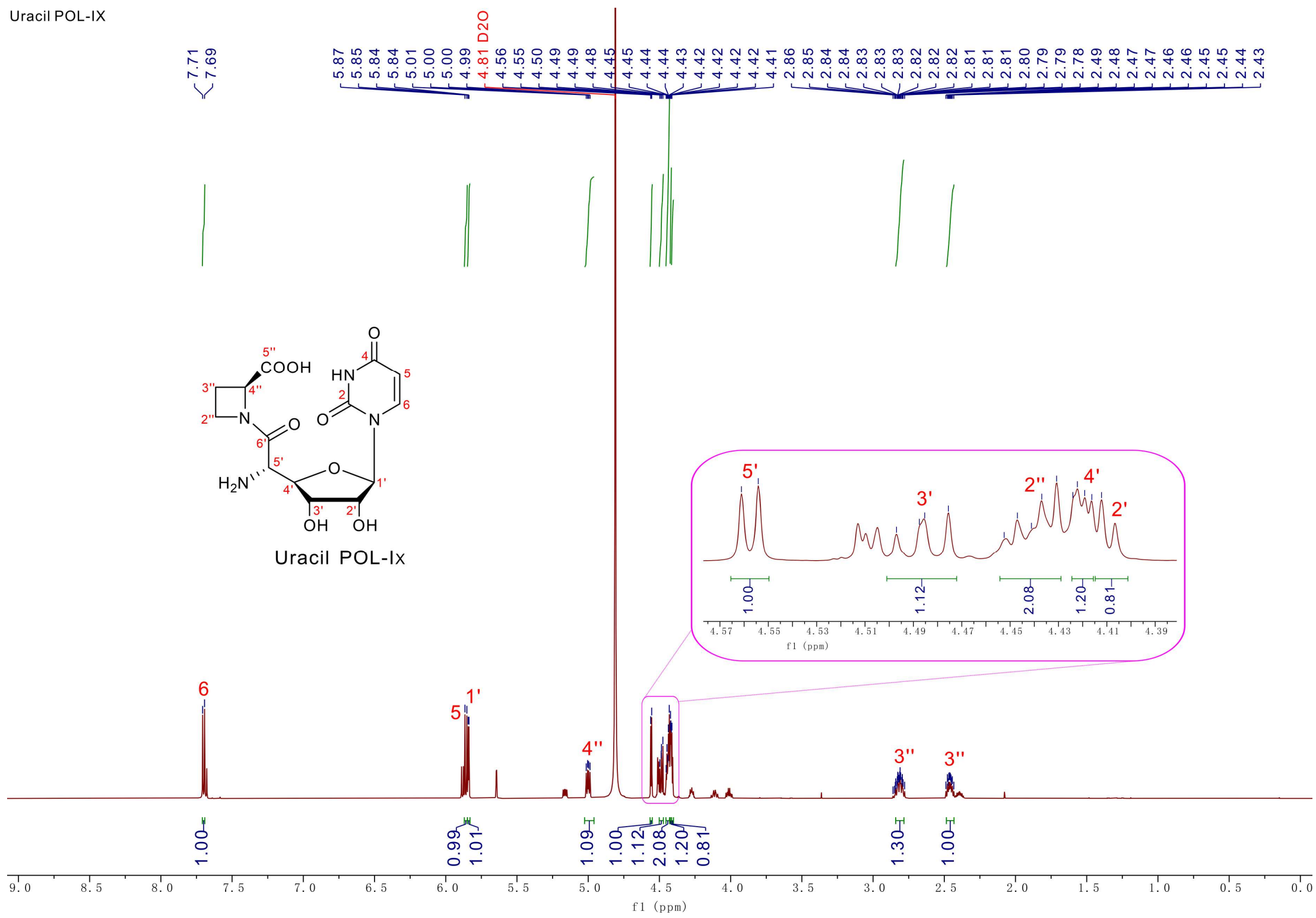

Figure S41. [1]**H NMR spectrum of uracil POL-Ix (D$_2$O, 600 MHz).**

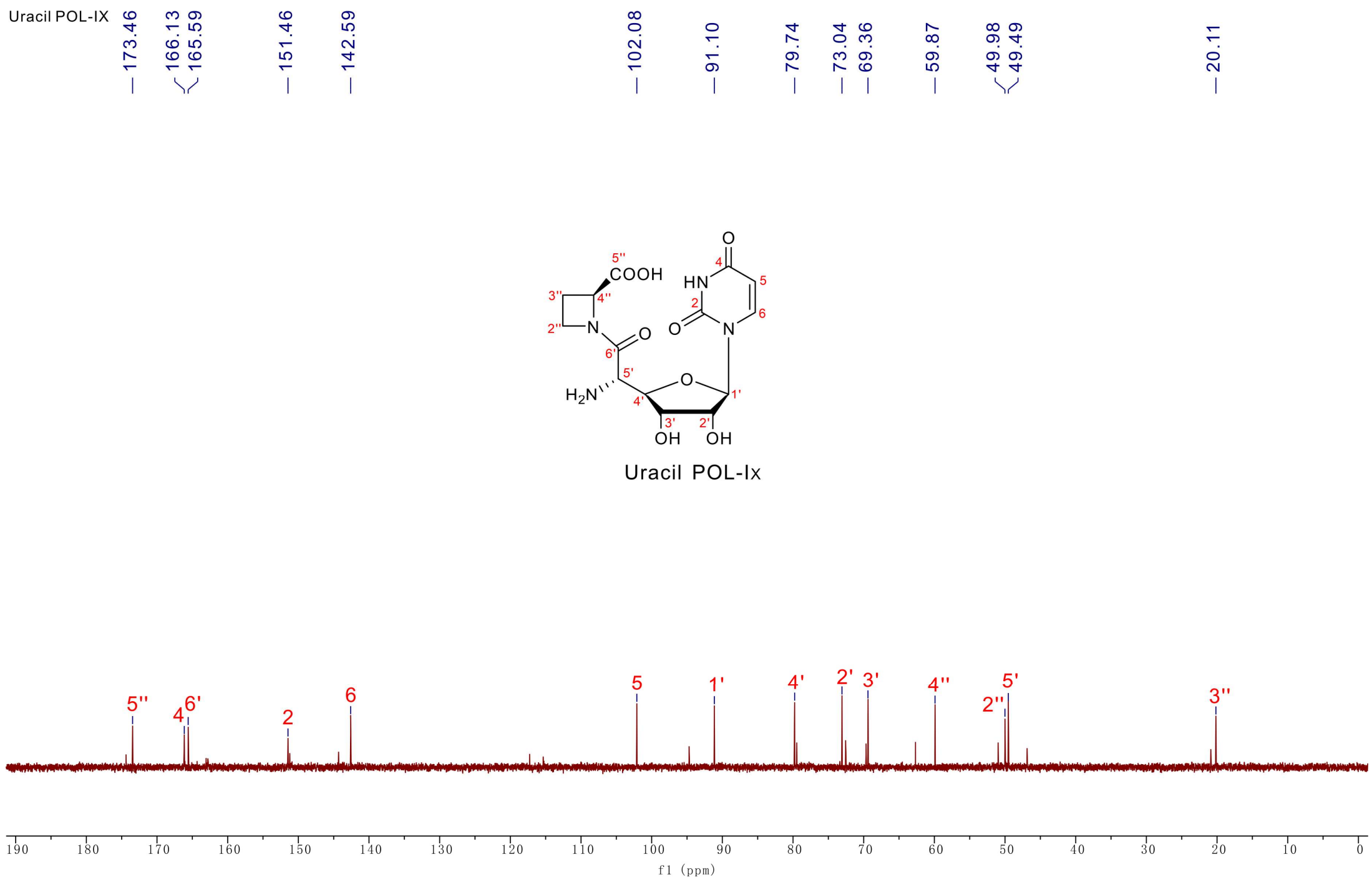

Figure S42. [13]**C NMR spectrum of uracil POL-Ix (D$_2$O, 150 MHz).**

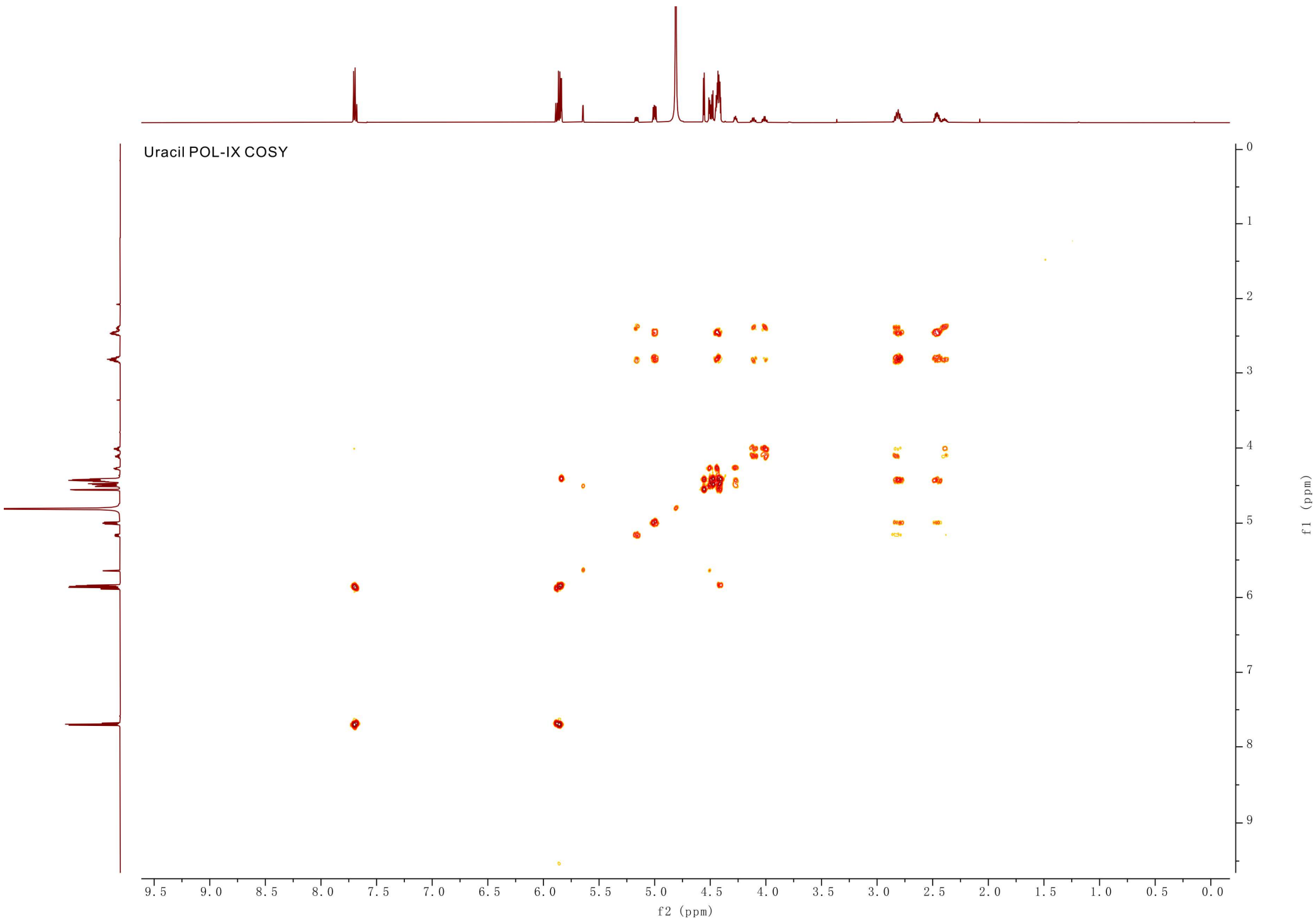

Figure S43. ^1H-^1H COSY spectrum of uracil POL-Ix (D_2O, 600 MHz).

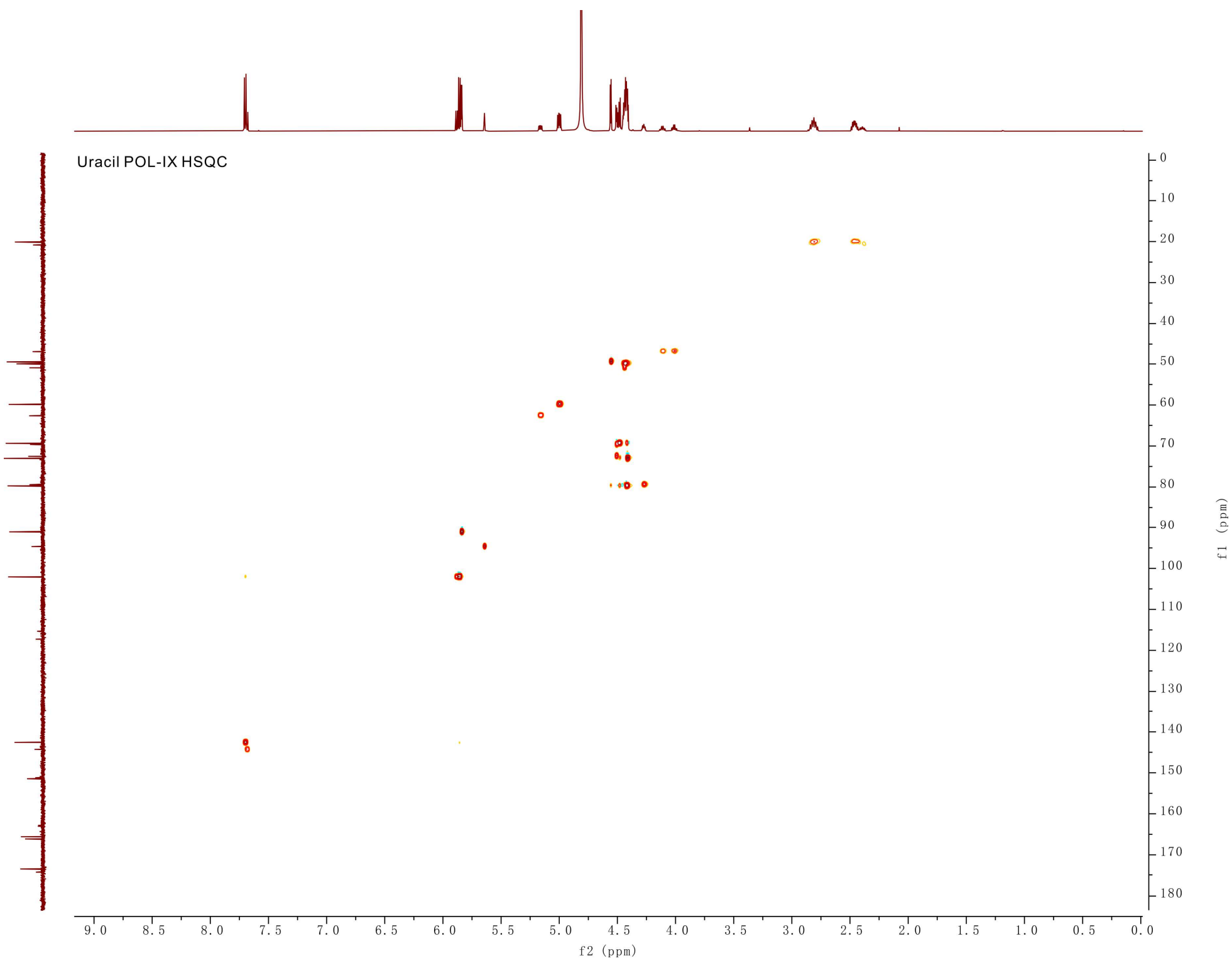

Figure S44. HSQC spectrum of uracil POL-Ix (D_2O, 600 MHz).

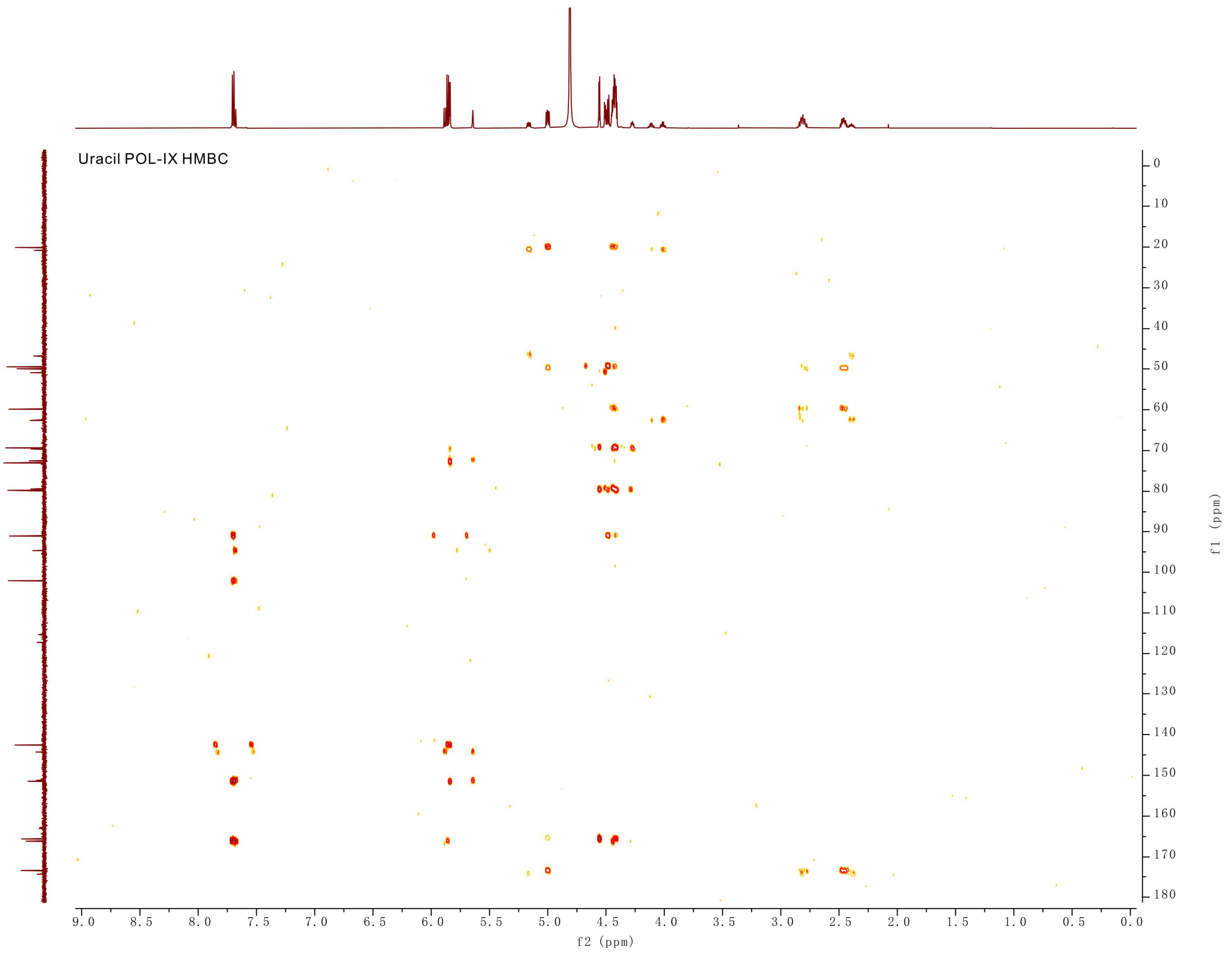

Figure S45. HMBC spectrum of uracil POL-I$_X$ (D_2O, 600 MHz).

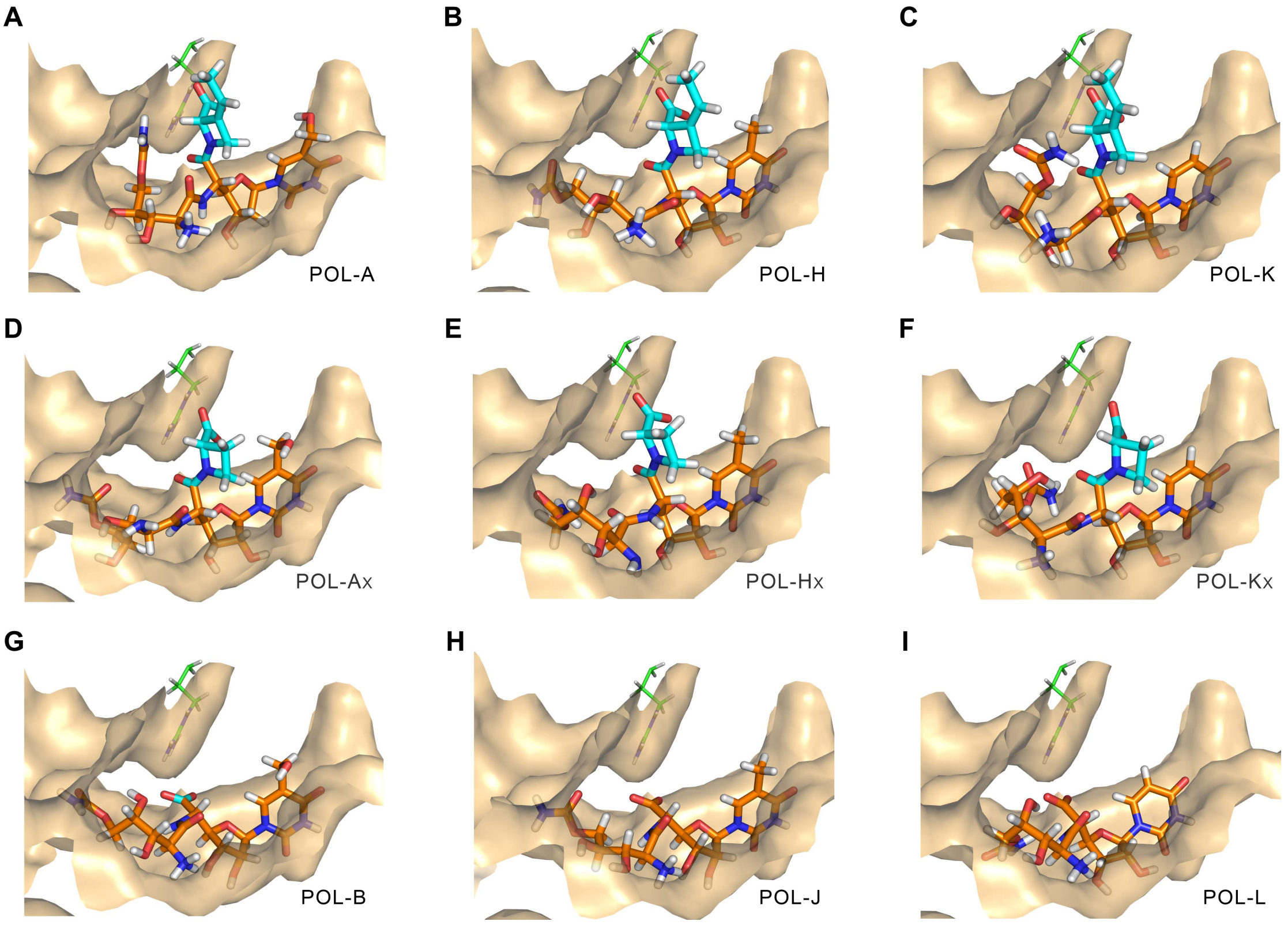

Figure S46. Molecular docking of related POL analogs with the chitin synthase *Ca*Chs2. (A) Surface representation of POL-A in *Ca*Chs2. (B) Surface representation of POL-H in *Ca*Chs2. (C) Surface representation of POL-K in *Ca*Chs2. (D) Surface representation of POL-A_X in *Ca*Chs2. (E) Surface representation of POL-H_X in *Ca*Chs2. (F) Surface representation of POL-K_X in *Ca*Chs2. (G) Surface representation of POL-B in *Ca*Chs2. (H) Surface representation of POL-J in *Ca*Chs2. (I) Surface representation of POL-L in *Ca*Chs2. *Ca*Chs2, (PDB: 7STO, from *Candida albicans*)[13].

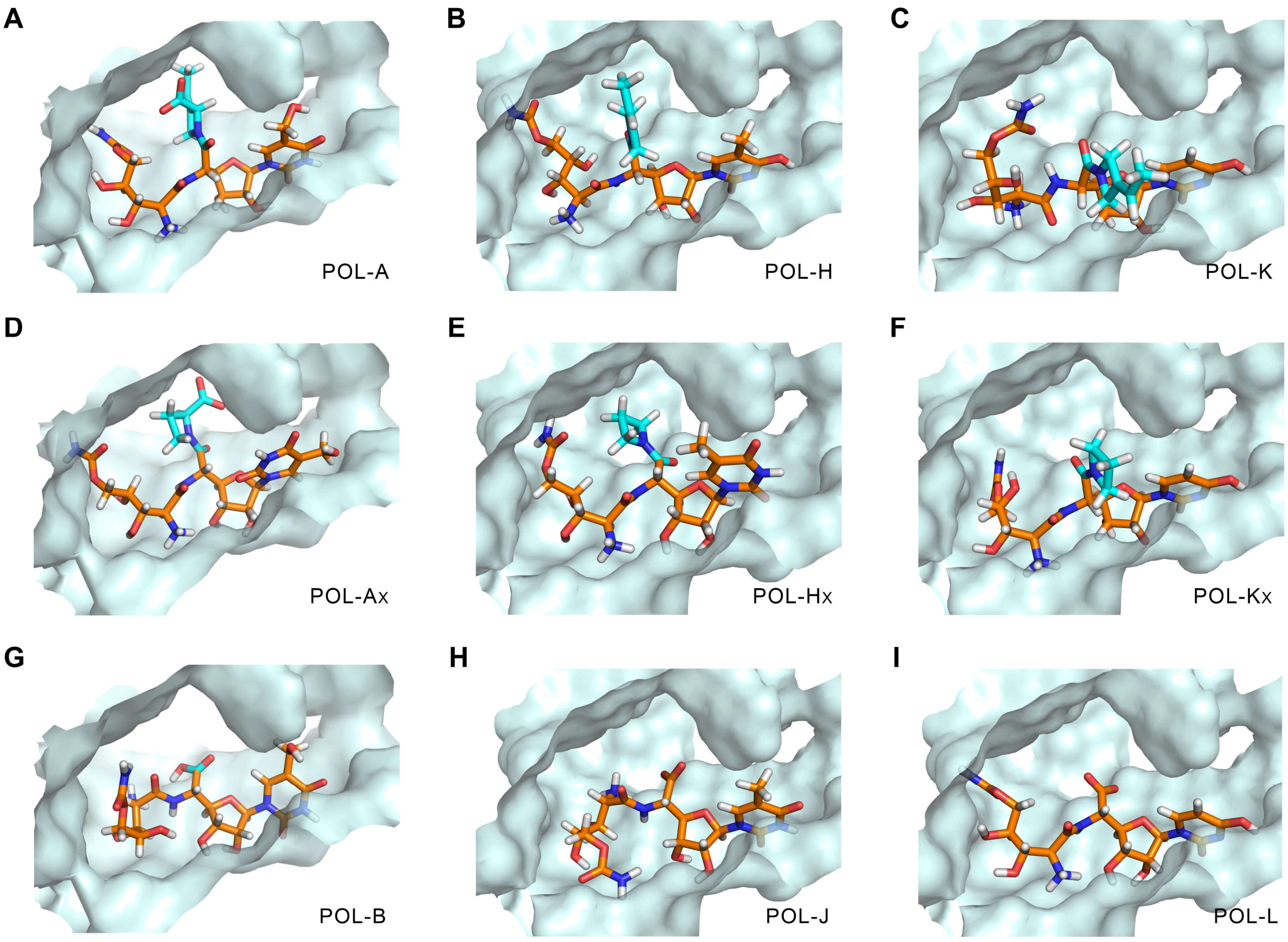

Figure S47. Molecular docking of related POL analogs with the chitin synthase *Ps*Chs1. (A) Surface representation of POL-A in *Ps*Chs1. (B) Surface representation of POL-H in *Ps*Chs1. (C) Surface representation of POL-K in *Ps*Chs1. (D) Surface representation of POL-A_X in *Ps*Chs1. (E) Surface representation of POL-H_X in *Ps*Chs1. (F) Surface representation of POL-K_X in *Ps*Chs1. (G) Surface representation of POL-B in *Ps*Chs1. (H) Surface representation of POL-J in *Ps*Chs1. (I) Surface representation of POL-L in *Ps*Chs1. *Ps*Chs1 (PDB: 7WJO, from *Phytophthora sojae*)[14].

2. Supplementary Tables

Table S1. Strains and plasmids used in this study.

Strain/Plasmid	Relevant characteristics	Source
**Strain**		
S. aureochromoges	Polyoxins producer	Lab
CXR3	*S. aureochromoges* derivative with *polF* deleted	This study
CXR6	CXR3 derivative with *polB* deleted	This study
CPO1	CXR3 derivative with *polLMNOP* deleted	This study
CPO2	CXR6 derivative with *polLMNOP* deleted	This study
CXR3::pIB139/*azeJ*	CXR3 containing pIB139/*azeJ*	This study
CXR3::pIB139	CXR3 containing pIB139	This study
CXR6::pIB139/*azeJ*	CXR6 containing pIB139/*azeJ*	This study
CXR6::pIB139/*azeJ*&*vioG*	CXR6 containing pIB139/*azeJ*&*vioG*	This study
CXR6::pIB139	CXR6 containing pIB139	This study
CPO1::pIB139/*azeJ*	CPO1 containing pIB139/*azeJ*	This study
CPO1::pIB139	CPO1 containing pIB139	This study
CPO2::pIB139/*azeJ*	CPO2 containing pIB139/*azeJ*	This study
CPO2::pIB139	CPO2 containing pIB139	This study
DH10B	F^- mcrA Δ(*mrr-hsd*RMS-*mcr*BC) ϕ80d *lacZ* Δ M15 Δ*lac*X74 *deo*R *rec*A1 *end*A1 *ara* Δ139 Δ(*ara leu*)1697 *gal*U *gal*Kλ *rps*L *nup*G	Gibco-BRL
ET12567(pUZ8002)	F dam-13::Tn9 dcm-6 hsdM hsdR recF143 zjj-202::Tn10 galK2 galT22 ara14 pacY1 xyl-5 leuB6 thi-1 pUZ8002	[15]
Trichosporon cutaneum 3071	Human pathogen	CGMCC
Candida albicans SC5314	Human pathogen	[16]
Alternaria alternate c9545	Tobacco brown spot fungus	CGMCC
Phytophthora sojae P6497	Phytopathogenic fungi of soybean	This study
Magnaporthe grisea P131	Rice blast fungus	[16]
Pellicularia sasakii JTU01	Rice sheath blight fungus	[16]
**Plasmid**		
pOJ446	*aac (3)IV,* SCP2, *rep* ^{MB1*} *attP*ϕC31 *oriT*	Lab
pIB139	*int*, *attp*, *acc(3)IV*, *PermE*	Lab

pJTU2833	pOJ446 derivative with insertion of engineered XbaI-BglII fragment including *polB*	This study
pCHW716	pOJ446 derivative with insertion of engineered XbaI-BglII fragment including *polLMNOP*	This study
pIB139/*azeJ*	pIB139 derivative carrying a NdeI-EcoRI fragment containing *azeJ*	This study
pIB139/*azeJ*＆*vioG*	pIB139 derivative carrying a NdeI-EcoRI fragment containing *azeJ* and *vioG*	This study

Table S2. Primers used in this study.

Primers	Sequence (5'--3')
polF-LF	GCTCTAGATGCTGCGACTTGTGCTTGGA
polF-LR	GAAGATCTCAGGTCAGCGACGAGGACA
polF-RF	GAAGATCTGACGCCCAGACCGGAGAG
polF-RR	TTCCGCACCTGGCTGTCC
polF-IDF	ATCGGCTTGTTGTTCAGGAGG
polF-IDR	TGTGACCGTGAACGCTTTGG
polB-LF	GCTCTAGACTGTTCGTCTGGCTGGACC
polB-LR	GAAGATCTCGGTCACCCACGAGGCGTT
polB-RF	GGAATTCGACTTCACCCTCCGCT
polB-RR	GAAGATCTCCCGAGCCGCCTTGACGA
polB-IDF	GGCATATGGAGTCACCCCGGATCG
polB-IDR	GCGGGCAGATGGACGGAT
azeJ-exF	GTCCATATGAGTCAGAACATGGAT
azeJ-exR	GGAATTCTCATGCCGCCGACGAACC
vioG-exF	GTCCATATGGCCCTGCGCGTCCTG
vioG-exR	GGAATTCTCAGGCCTTGGAGCCGGCCTG
polLMNOP-446-LF	GCCCTGCGGGGCGGCTCTAGATGCGGGATGCCTACGAAGTG
polLMNOP-446-FR	GCATGCAAGCTTGGCTGCAGATCTTCGGACGAGGTCTCCGGTGATGT
polLMNOP-446-RF	ACCGAGCTCGAATTCCCCAGATCTTCGTCCCGGTCGTCTCCAGCAT
polLMNOP-446-RR	ATGCTGTGACTGCCAAGATCTTCAGCGACAGCCAGCCCTTC
tsr-BglII-F	GAAGATCTGCAGCCAAGCTTGCATGC
tsr-BglII-R	GAAGATCTGGGGAATTCGAGCTCGGT
polLMNOP-446-IDF	ACTTCGCCCGCACCTGA
polLMNOP-446-IDR	TGCACGCCTGGACCTTG
polLMNOP-gRNA-F	CATGGTGAACGCTCATGATCACCTGGTTTTAGAG
polLMNOP-gRNA-R	CTAGCTCTAAAACCAGGTGATCATGAGCGTTCAC
polLMNOP-cas9-LF	TCGTCGAAGGCACTAGAAGGACCTGTCGGACGAGGAGATGG

polLMNOP-cas9-LR	TCATGGGCTCACCGTGGTGCCGGCCGTGGGTCGGGTGAGCAC
polLMNOP-cas9-RF	GGCACCACGGTGAGCCCATGA
polLMNOP-cas9-RR	GGTCGATCCCCGCATATAGGACCAAGACCGCCCTGCTCAC
polLMNOP-cas9-idF	GCCCGCATGACGCGGTACT
polLMNOP-cas9-idR	GCACGTTGCTGAGCTGGACG

Table S3. Validation of the mutant strains by sequencing analysis.

Strain	Determined sequence	Theoretical sequence
CXR3	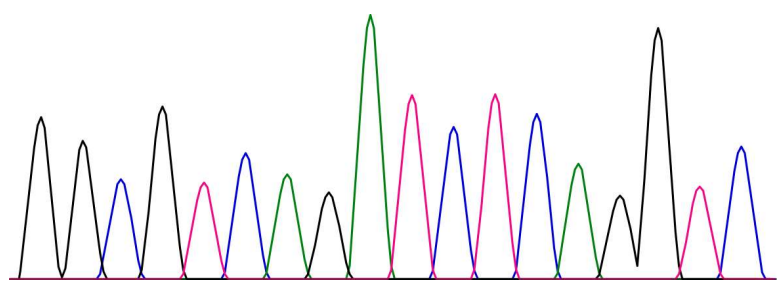 GGC GTC AGA TCT CAG GTC	GGC GTC AGA TCT CAG GTC
CPO2	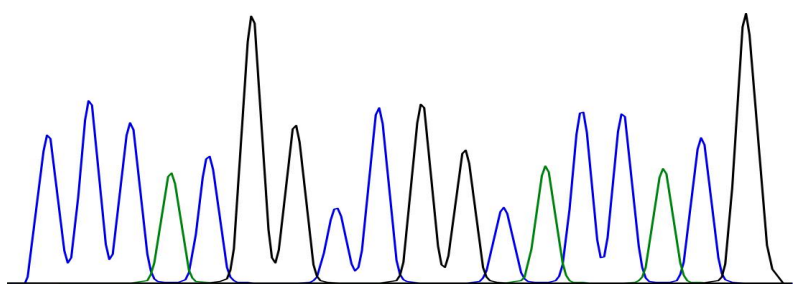 CCC ACG GCC GGC ACC ACG	CCC ACG GCC GGC ACC ACG

Table S4. NMR data of POL-Ax (D_2O, 400 MHz).

Position	^{1}H(δ, mult, J)	^{13}C(δ)
2		151.35
4		164.75
5		113.48
6	7.66 (s, 1H)	140.28
7	4.25 (s, 2H)	56.46
1′	5.78 (d, *J*=2.8 Hz, 1H)	90.27
2′	4.31 (s, 1H)	72.78
3′	3.96 (d, *J* = 6.3Hz, 1H)	69.70
4′	4.24 (s, 1H)	81.35
5′	4.77 (m, 1H)	49.76
6′		168.61
2″	4.39 (t, *J* = 7.8 Hz, 2H)	49.95
3″	2.73 (m, 1H),<br>2.32 (dq, *J*=13.4, 7.3 Hz, 1H)	19.86
4″	4.89 (m, 1H)	59.44
5″		173.56
2‴		167.74
3‴	4.26 (d, *J* = 4.6 Hz, 1H)	55.93
4‴	4.35 (s, 1H)	69.90
5‴	4.11 (m, 1H)	68.36
6‴	4.03 (d, *J* = 3.2Hz, 2H)	65.14
8‴		158.86

Table S5. NMR data of POL-H_X (D_2O, 400 MHz).

Position	^{1}H(δ, mult, J)	^{13}C(δ)
2		151.48
4		166.11
5		111.27
6	7.45 (s, 1H)	137.85
7	1.77 (d, *J*=5.1 Hz, 3H)	11.45
1′	5.78 (d, *J* = 4.0 Hz, 1H)	90.00
2′	4.30 (d, *J* = 4.6 Hz, 1H)	72.98
3′	4.01 (m, 1H)	69.86
4′	4.26 (d, *J* = 5.1 Hz, 1H)	81.49
5′	4.75 (d, *J* = 4.8 Hz, 1H)	49.82
6′		168.68
2″	4.43 (m, 2H)	50.06
3″	2.71 (td, *J*=9.3, 3.6 Hz, 1H),<br>2.37 (m, 1H)	19.97
4″	4.89 (m, 1H)	59.48
5″		173.54
2‴		167.74
3‴	4.29 (s, 1H)	56.04
4‴	4.36 (d, *J*=6.0 Hz, 1H)	69.99
5‴	4.15 (m, 1H)	68.44
6‴	4.05 (d, *J*=7.5 Hz, 2H)	65.24
8‴		158.90

Table S6. NMR data of POL-Kx (D_2O, 600 MHz).

Position	^{1}H(δ,mult,J)	^{13}C(δ)
2		151.45
4		166.10
5	5.87 (d, *J* = 8.1 Hz, 1H)	102.19
6	7.68 (d, *J* = 8.1 Hz, 1H)	142.63
1′	5.84 (d, *J* = 3.8 Hz, 1H)	90.82
2′	4.43 (m, 1H)	72.74
3′	4.03 (td, *J* = 5.4, 2.7 Hz, 1H)	69.61
4′	4.33 (m, 1H)	81.32
5′	4.84 (d, *J* = 9.7 Hz, 1H)	49.94
6′		168.70
2″	4.45 (dd, *J*=5.9, 3.6 Hz, 2H)	50.35
3″	2.77 (ddt, *J*=10.4, 5.4, 2.4 Hz, 1H),<br>2.41 (ddt, *J*=12.0, 9.1, 6.3 Hz, 1H)	19.92
4″	4.92 (dd, *J* = 9.9, 5.8 Hz, 1H)	59.52
5″		173.60
2‴		167.76
3‴	4.30 (m, 1H)	55.90
4‴	4.38 (dd, *J* = 11.8, 6.0 Hz, 1H)	70.10
5‴	4.16 (q, *J*=1.6 Hz, 1H)	68.45
6‴	4.15 (m, 2H)	65.16
8‴		158.90

Table S7. NMR data of POL-Ix (D_2O, 600 MHz).

Position	^{1}H(δ, mult, J)	^{13}C(δ)
2		151.46
4		165.58
5		113.29
6	7.78 (s, 1H)	140.66
7	4.30 (m, 1H)	56.47
1′	5.85 (s, 1H)	90.917
2′	4.40 (m, 1H)	73.08
3′	4.38 (m, 1H)	69.34
4′	4.42 (d, *J*=3.4 Hz, 1H)	79.81
5′	4.56 (d, *J* = 4.2 Hz, 1H)	49.43
6′		164.96
2″	4.46 (m, 2H)	49.97
3″	2.85 (m, 1H), 2.45 (ddt, *J*=11.8, 9.1, 6.0, 1H)	19.96
4″	5.01 (dd, *J* = 10.0, 5.9 Hz, 1H)	59.78
5″		173.43

Table S8. NMR data of thymine POL-I_X (D_2O, 600 MHz).

Position	^{1}H(δ, mult, J)	^{13}C(δ)
2		151.48
4		166.29
5		111.15
6	7.51 (t, *J*=1.3 Hz, 1H)	137.84
7	1.83 (d, *J*=1.2 Hz, 3H)	11.39
1′	5.83 (d, *J* = 3.3 Hz, 1H)	90.57
2′	4.37 (dd, *J* = 5.7, 3.4 Hz, 1H)	73.23
3′	4.51(m, 1H)	69.35
4′	4.48 (m, 1H)	79.78
5′	4.56 (d, *J* = 3.9 Hz, 1H)	49.40
6′		165.57
2″	4.45 (m, 2H)	50.00
3″	2.84 (m, 1H), 2.47 (m, 1H)	20.10
4″	5.01 (dd, *J* = 10.0, 5.8 Hz, 1H)	59.71
5″		173.36

Table S9. NMR data of uracil POL-I_X (D_2O, 600 MHz).

Position	^{1}H(δ, mult, J)	^{13}C(δ)
2		151.46
4		166.13
5	5.86 (d, *J* = 8.1Hz, 1H)	102.08
6	7.70 (d, *J* = 8.1 Hz, 1H)	142.59
1′	5.84 (d, *J* = 3.4 Hz, 1H)	91.10
2′	4.41 (s, 1H)	73.04
3′	4.49 (dd, *J*=7.1, 5.8 Hz, 1H)	69.36
4′	4.42 (dd, *J* = 3.3, 1.4 Hz, 1H)	79.74
5′	4.56 (d, *J*=4.2 Hz, 1H)	49.49
6′		165.59
2″	4.44 (dd, *J*=9.5, 3.5 Hz, 2H)	49.98
3″	2.84(m, 1H),<br>2.46 (ddd, *J*=11.7, 5.8, 2.7 Hz, 1H)	20.11
4″	5.00 (dd, *J* = 10.0, 5.9 Hz, 1H)	59.87
5″		173.46

Table S10. Codon-optimized sequence of *azeJ*.

Gene name	Codon-optimized sequence (5′-3′)
azeJ	ATGTCCCAGAACATGGACCTGACCATCGGCACCCACCGCGAGTCC<br>GCCTTCTACGAGCTGGAGTTCGGCCCGCGCACCATCATGACCCTG<br>GCCAACTTCCCGGACGACGTCCTGCCGCTGCTGCAGATGGAGTCC<br>CTGATGACCTTCGAGGCCATGGCCTACCTCCGCTGCGACGCCCTG<br>GTCGAGCTCGGCTGCTACGACGGCCGCGCCCTGGAGATCGCCCGC<br>CTGCTGAACGCCCGCTACCTCGGCGTCGACCTGGACCAGCGCGCC<br>ATCGAGACCCTGCGCACCCGCATCGAGCGCGAGGGCATGTCCGAC<br>CGCGCCGACACCGTCGTCGACGACATCCTGAACCACACCCGCCGC<br>GGCGCCTCCGTCGGCTCCCGCGCCCTGTACCTGCTGCCGTTCAAC<br>CTGCTGGGCAACTTCCGCGAGCCGAAGCGCCTGCTGGACTCCCTG<br>GCCGAGCGCTCCGTCGCCGCCGTCGTCTCCGTGTTCGGCGACTCG<br>GCCGAGGCCACCCGCGTCCGCCAGTCCTACTACCGCCGCTGCGGC<br>GTCCAGGGCCTGGAGCTGCACACCCGCGACGACGGCACCGTCTT<br>CACCGGCTCCGACGGCTTCTACTCCCGCTCCTACTCCCGCGCCTGC<br>CTGCACGCCCTGCTCGCCGAGTGCGGCCTGACCGTCGTCCGCTCC<br>GCCTCCAACCTGTTCGCCCACTGCGTCACCGTCCTGCCGGAGGGC<br>GCCGACCAGGGCTTCGGCTCCTCCGCCGCCTGA

Table S11. Quantitative docking scores.

Ligand	-CDOCKER_ ENERGY (7STO)	-CDOCKER_ INTERACTION _ENERGY (7STO)	-CDOCKER_ ENERGY (7WJO)	-CDOCKER_ INTERACTIO N_ENERGY (7WJO)
POL-A	6.25	94.87	1.88	87.13
POL-A_X	32.45	79.30	31.57	65.66
POL-H	8.24	93.64	-17.81	72.10
POL-H_X	36.62	75.15	26.71	67.44
POL-K	15.01	89.24	-14.42	64.10
POL-K_X	58.40	83.21	36.74	75.04
POL-B	63.21	88.64	65.74	71.15
POL-J	67.34	91.77	46.77	66.57
POL-L	76.02	84.00	47.77	68.65

3. Supplementary References

[1] Chen W, Huang T, He X, Meng Q, You D, Bai L, et al. Characterization of the polyoxin biosynthetic gene cluster from *Streptomyces cacaoi* and engineered production of polyoxin H. J Biol Chem 2009;284(16):10627-38.

[2] Chen W, Li Y, Li J, Wu L, Li Y, Wang R, et al. An unusual UMP C-5 methylase in nucleoside antibiotic polyoxin biosynthesis. Protein Cell 2016;7(9):673-83.

[3] Draelos MM, Thanapipatsiri A, Sucipto H, Yokoyama K. Cryptic phosphorylation in nucleoside natural product biosynthesis. Nat Chem Biol 2021;17:213-21.

[4] He N, Wu P, Gao Y, Xu B, Zhu X, Xu G, et al. Construction of an octosyl acid backbone catalyzed by a radical *S*-adenosylmethionine enzyme and a phosphatase in the biosynthesis of high-carbon sugar nucleoside antibiotics. Chem Sci 2017;8(1):444-51.

[5] Lilla EA, Yokoyama K. Carbon extension in peptidylnucleoside biosynthesis by radical SAM enzymes. Nat Chem Biol 2016;12(11):905-7.

[6] Qi J, Wan D, Ma H, Liu Y, Gong R, Qu X, et al. Deciphering carbamoylpolyoxamic acid biosynthesis reveals unusual acetylation cycle associated with tandem reduction and sequential hydroxylation. Cell Chem Biol 2016;23(8):935-44.

[7] Gong R, Qu Y, Liu J, Zhang X, Zhou L, Tian Z, et al. A two-metalloenzyme cascade constructs the azetidine-containing pharmacophore. Nat Chem 2026;18:482-91.

[8] Gong R, Qi J, Wu P, Cai Y, Ma H, Liu Y, et al. An ATP-dependent ligase with substrate flexibility involved in assembly of the peptidyl nucleoside antibiotic polyoxin. Appl Environ Microbiol 2018;84(13):e00501-18.

[9] Hong Z, Bolard A, Giraud C, Prevost S, Genta-Jouve G, Deregnaucourt C, et al. Azetidine-containing alkaloids produced by a quorum-sensing regulated nonribosomal peptide synthetase pathway in *Pseudomonas aeruginosa*. Angew Chem Int Ed Engl 2019;58(10):3178-82.

[10] Klaubert TJ, Gellner J, Bernard C, Effert J, Lombard C, Kaila VRI, et al. Molecular basis for azetidine-2-carboxylic acid biosynthesis. Nat Commun 2025;16(1):1348.

[11] Yan F, Auerbach D, Chai Y, Keller L, Tu Q, Hüttel S, et al. Biosynthesis and heterologous production of vioprolides: rational biosynthetic engineering and unprecedented 4-methylazetidinecarboxylic acid formation. Angew Chem Int Ed Engl 2018;57(28):8754-9.

[12] Yan F, Müller R. Class I methyltransferase VioH catalyzes unusual *S*-adenosyl-l-methionine cyclization leading to 4-methylazetidinecarboxylic acid formation during vioprolide biosynthesis. ACS Chem Biol 2018;14(1):99-105.

[13] Ren Z, Chhetri A, Guan Z, Suo Y, Yokoyama K, Lee SY. Structural basis for inhibition and regulation of a chitin synthase from *Candida albicans*. Nat Struct Mol Biol 2022;29(7):653-64.

[14] Chen W, Cao P, Liu Y, Yu A, Wang D, Chen L, et al. Structural basis for directional chitin biosynthesis. Nature 2022;610(7931):402-8.

[15] Bertolt G, Greg LC, Kay F, Tobias K, Keith FC. PCR-targeted *Streptomyces* gene replacement identifies a protein domain needed for biosynthesis of the sesquiterpene soil odor geosmin. Proc Natl Acad Sci USA 2003;100(4):1541-6.

[16] Qi J, Liu J, Wan D, Cai YS, Wang Y, Li S, et al. Metabolic engineering of an industrial polyoxin producer for the targeted overproduction of designer nucleoside antibiotics. Biotechnol Bioeng 2015;112(9):1865-71.
